# The life-cycle of *Toxoplasma gondii* reviewed using animations

**DOI:** 10.1186/s13071-020-04445-z

**Published:** 2020-11-23

**Authors:** Márcia Attias, Dirceu E. Teixeira, Marlene Benchimol, Rossiane C. Vommaro, Paulo Henrique Crepaldi, Wanderley De Souza

**Affiliations:** 1 Instituto de Biofísica Carlos Chagas Filho, Universidade Federal do Rio de Janeiro, Rio de Janeiro, Brazil; 2 Centro Nacional de Biologia Estrutural e Bioimagem, Universidade Federal do Rio de Janeiro, Rio de Janeiro, Brazil; 3 Instituto Nacional de Educação de Surdos, Rio de Janeiro, Brazil; 4 Universidade do Grande Rio, Rio de Janeiro, Brazil

**Keywords:** Apicomplexa, Parasitology, Parasite, Toxoplasmosis, Protozoology, Cell biology, Life-cycle

## Abstract

*Toxoplasma gondii* is a protozoan parasite that is the causative agent of toxoplasmosis, an infection with high prevalence worldwide. Most of the infected individuals are either asymptomatic or have mild symptoms, but *T. gondii* can cause severe neurologic damage and even death of the fetus when acquired during pregnancy. It is also a serious condition in immunodeficient patients. The life-cycle of *T. gondii* is complex, with more than one infective form and several transmission pathways. In two animated videos, we describe the main aspects of this cycle, raising questions about poorly or unknown issues of *T. gondii* biology. Original plates, based on electron microscope observations, are also available for teachers, students and researchers. The main goal of this review is to provide a source of learning on the fundamental aspects of *T. gondii* biology to students and teachers contributing for better knowledge and control on this important parasite, and unique cell model. In addition, drawings and videos point to still unclear aspects of *T. gondii* lytic cycle that may stimulate further studies.
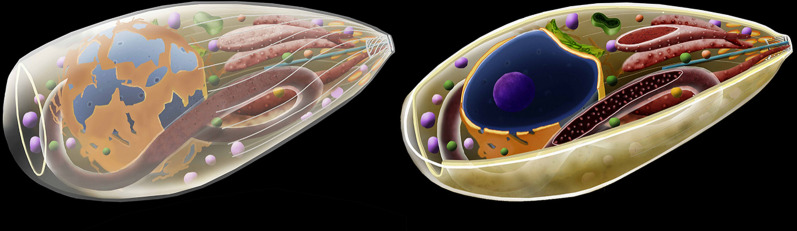

## Background

*Toxoplasma gondii* is the causative agent of toxoplasmosis that is a zoonosis of significant medical and veterinary importance and is transmitted by several pathways. Marked advances regarding the control of several infectious diseases caused by parasitic protozoa have taken place in the last decades, especially those that spend part of their life-cycle inside host cells. Nevertheless, the epidemiological control and development of new chemotherapeutic agents with low toxicity and high specificity continue to constitute great challenges. Some of these diseases are restricted to specific areas of the world, as in the case of Chagas disease. Others, like toxoplasmosis, are widely distributed throughout the world [1]. Indeed, *T. gondii*, a member of the phylum Apicomplexa, developed the ability to infect almost any cell type of mammals and birds [2, 3]. In the USA, it was estimated that 11% of the population aged six years and older have been infected with *T. gondii*. In several countries throughout the world, it has been shown that more than 60% of the people have been infected with *T. gondii* [4]. In some geographical areas (e.g. Brazil), up to 60% of the population is seropositive for *T. gondii* antigens [5]. The environmental conditions and dietary habits can impact infection rates. For example, the ingestion of raw or undercooked meat is associated with *T. gondii* transmission, and pig and sheep meat are more prone to contain tissue cysts than cattle [6–8]. Contamination by oocysts excreted with cat feces does not necessarily involve contact with the cat itself. Pet cats are less subject to becoming contaminated (and produce oocysts) than cats living on the street or rural areas [1, 5, 9, 10].

The genus *Toxoplasma* contains only one species, *T. gondii*, that can be grouped into genotypes (types I, II and III, XII and the haplotypes X and A). Some are restricted to wild animals.

It is always important to review and disseminate the current knowledge on *T. gondii* and other parasites not only to college, undergraduate and high school students, but also to the general population, as part of an effort to eradicate, or at least control or prevent the disease burden of toxoplasmosis, particularly among women at reproductive age, since they are the main group at risk.

We have previously developed educational material to teach fundamental biological aspects on Chagas disease and leishmaniasis caused, respectively, by *Trypanosoma cruzi* and *Leishmania* spp., with emphasis on dynamic processes and three-dimensional views [11, 12].

## Available material

Here we present a review and innovative multimedia materials showing basic aspects of the life-cycle of the protozoan *T. gondii* and its morphology, based on electron microscopy observations of the various developmental stages of *T. gondii*. In addition to the PowerPoint slide show that is available at (Additional file 23: Slideshow), we produced videos that can be visualized in Additional files 21 and 22: Videos SV1 and SV2 or in https://pesquisa.biof.ufrj.br/biologia-celular-parasitologia/luchm/ . The 3D models and animations were based on our group’s information obtained over the last 30 years using video-microscopy and scanning and transmission electron microscopy. They show various aspects of the structural organization of the different developmental stages of *T. gondii* [13–23]. Our analysis also considered the contributions of several other groups that have provided relevant contributions to the field [24–26]. All animations and images were produced using software such as 3D Max (Autodesk Inc., San Rafael, CA. USA; 94903), AfterEffects, Photoshop and Illustrator (all three from Adobe; www.adobe.com).

The development of these videos raised questions that led to speculations on the dynamics of some still poorly characterized biological processes in an attempt to stimulate further research to confirm or invalidate some hypotheses.

## Biological cycle (transmission pathways)

**Fig. 1:**
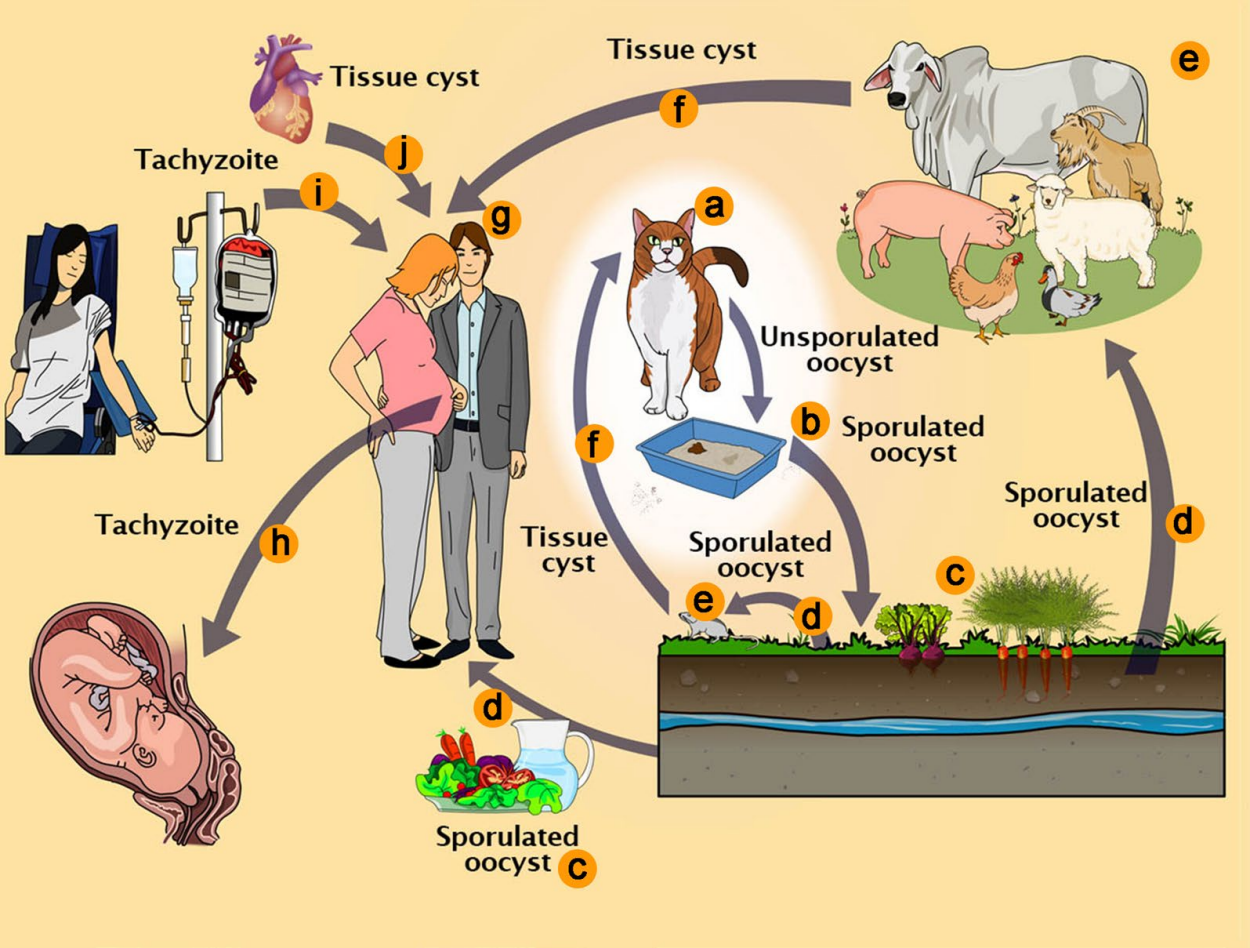
*Toxoplasma gondii* pathways of transmission. **a** Feline definitive host (cat). **b** Unsporulated oocysts in cat feces. **c** Food contaminated with sporulated oocysts. **d** Oocysts may be ingested by intermediate hosts *via* water or raw vegetables. **e** Intermediate hosts (e.g. cattle, sheep, poultry and swine). **f** Ingestion of tissue cysts in uncooked meat. **g** Intermediate hosts (humans). **h** Tachyzoites transmitted through the placenta to the foetus. **i** Transmission by blood transfusion and organ transplant (**j**)

It is well known that *T. gondii* infects hosts that include terrestrial and aquatic mammals and birds. These animals are considered intermediate hosts because only asexual stages occur in them (Fig. 1). The sexual stages are seen only in the members of the family Felidae, including the domestic cat [3, 27, 28]. Therefore, they are considered to be definitive hosts. However, it has been recently reported that inhibition of murine-delta-6-desaturase activity in the mouse intestine and supplementation of the diet of mice with linoleic acid allowed *T. gondii* sexual development in the intestinal cells of mice [29].

**Fig. 2:**
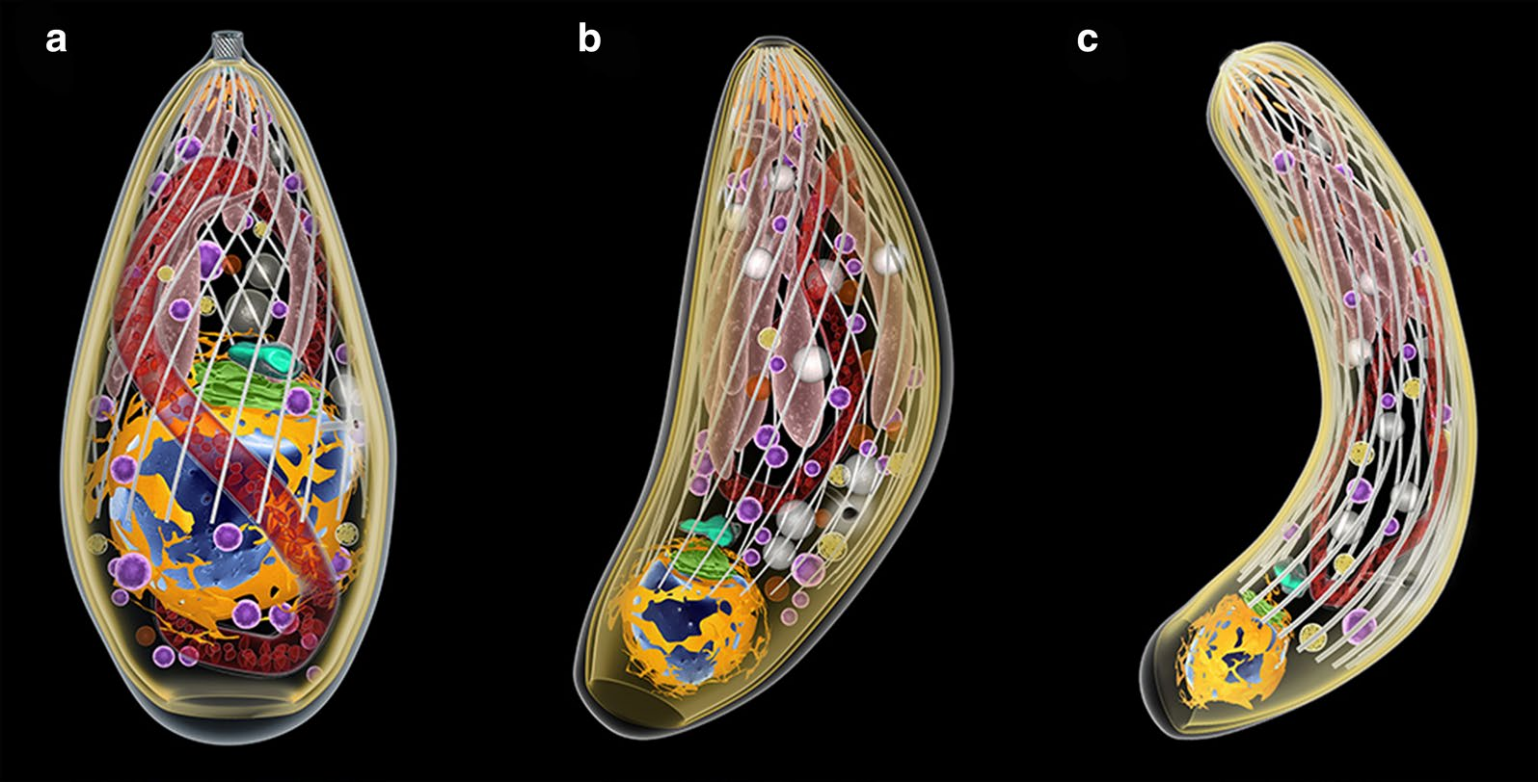
The three infective stages of *T. gondii*. Tachyzoite (**a**), bradyzoite (**b**), and sporozoite (**c**). The nucleus (blue) is surrounded by the rough endoplasmic reticulum (yellow). Above it, The Golgi complex (green) and the apicoplast (blue-green). The single mitochondrion spreads through the cytosol (red). Dense granules (magenta) and amylopectin granules (white) are dispersed in the cytosol. The apical complex is composed by the cylindrical conoid. Below, the secretory organelles: micronemes (orange) and rhoptries (pink). The cell body is limited by three membrane units (the pellicle) and below it a set of subpelicular microtubules

During the life-cycle of *T. gondii*, three developmental stages can infect cells (Fig. 2): (i) tachyzoite (a form of rapid multiplication that is characteristically found in the acute infections); (ii) bradyzoite (a form of slow multiplication that is characteristic of the chronic infection and that originates the tissue cysts; and (iii) sporozoite, which is produced only in the definitive host during the sexual reproduction and released in the oocysts *via* felid feces (Figs. 1, 2).

The intermediate hosts can be infected *via* different pathways including (i) ingestion of water, vegetables and fruits contaminated with viable oocysts, sporulated after previous elimination in the feces of cats (Fig. 1d); (ii) intake of uncooked or undercooked meat containing viable tissue cysts (Fig. 1f); (iii) congenital transmission from the mother through the placenta (Fig. 1h); (iv) blood transfusion (Fig. 1i); (v) organ transplantation, where the organs may contain cysts or tachyzoites (Fig.1j). Definitive hosts, i.e. felines, can be infected by carnivorism (both mammals and birds), or ingestion of sporulated oocysts. Oocysts can also survive in oysters and mussels retaining its infectivity [30–32]. Ingestion of non-pasteurized milk or milk products is a potential source of transmission, although not common [8, 33].

### Morphology and ultrastructure of the different forms of *T. gondii*

#### Infective forms (tachyzoites, bradyzoites and sporozoites)

**Fig. 3:**
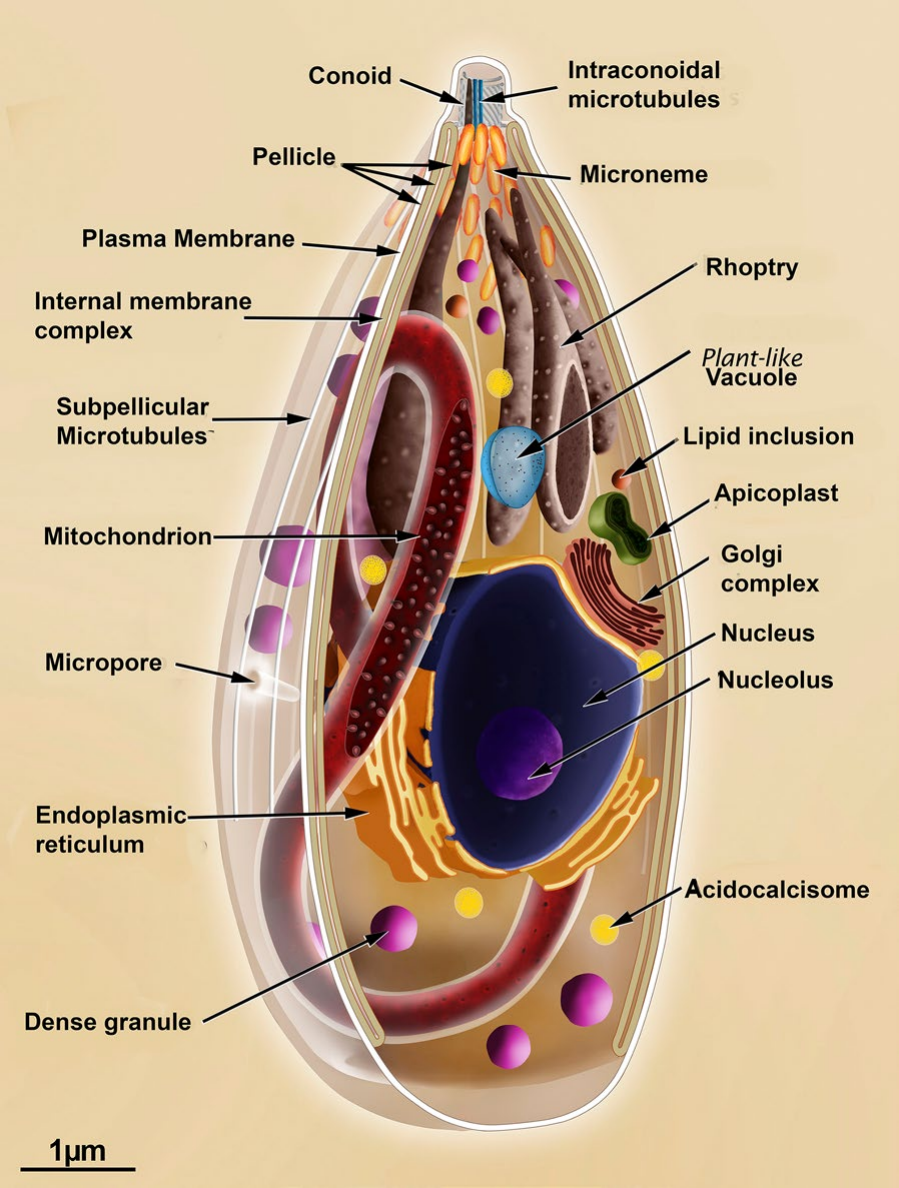
Longitudinal section view of the tachyzoite form of *Toxoplasma gondii* indicating the main structures and organelles

Scanning and transmission electron microscopy have been used to obtain a large number of images of the various developmental stages of *T. gondii*. The three infective forms (i.e. tachyzoite, bradyzoite and sporozoite) present the same primary organization, displaying an elongated shape and a typical apical complex where structures and organelles such as the conoid, micronemes, and rhoptries are found (Fig. 2). Most of the available information is from tachyzoites (Fig. 3, Additional file 1: Figures S1, Additional file 2: Figures S2, Additional file 3: Figure S3) studied in more detail [15, 20, 34–42].

A complex of membranes known as the pellicle delimits the whole protozoan body. It is formed by an external plasma membrane and, below it, two closely apposed membranes that form the inner membrane complex. This inner complex is absent from the most apical region where the conoid is located and in the most posterior part of the cell [43].

The three infective stages present an explicit specialization of the anterior region where the apical complex is localized. It is used to initiate the process of infection of host cells. This complex is formed by the conoid and two sets of secretory organelles: the micronemes and the rhoptries [15, 44].

The protozoan contains several structures that form its cytoskeleton. Below the inner membrane complex, there is a layer of microtubules. Twenty-two microtubules that originate at the polar ring and project towards the posterior region of the cell, reaching about two thirds of the cell body length (Additional file 4: Figure S4). Besides, there is a network of filamentous structures with a mean diameter of 8–10 nm and are very labile and located immediately below the inner membrane complex [45]. They are composed of proteins of the articulin group called alveolins [46–48] and extend throughout the cell ending in a circular structure located at the posterior region, known as the basal complex, where proteins such as the membrane occupation and recognition nexus exist (MORN-1) (Additional file 5: Figure S5) [35, 49].

One characteristic feature of the *T. gondii* cytoskeleton is the conoid that appears as a hollow cylinder inserted inside the polar ring from which the 22 sub-pellicular microtubules emerge. It has a diameter of 400 nm and a length of 250 nm and appears in two states. In a resting state, it appears under the polar ring. When activated by an influx of Ca^++^, it extrudes towards the anterior region of the cells (Additional file 2: Figure S2 and Additional file 4: Figure S4) [16, 50]. This motion occurs during the process of host cell invasion. In the resting state, the conoid is positioned immediately below the plasma membrane, under the posterior ring, from where the sub-pellicular microtubules emerge. Two microtubules are situated inside the conoid, and two more apical rings are present in its most apical portion. The anterior ring has a diameter of 160 nm, and the second measures 200 nm. Several proteins have been shown to exist in these complex structures, such as the calcium binding proteins Centrin 2, TgCAM1, TgCAM2 [51] and TgMyoH myosin [52].

Another characteristic feature of the infective stages of *T. gondii* is the presence of the apical secretory organelles. Micronemes are the most abundant ones (Fig. 3, Additional file 1: Figure S1, Additional file 2: Figure S2, Additional file 3: Figure S3, Additional file 6: Figure S6). They appear as rod-like structures that are 250 nm long and 50 nm wide. They concentrate around the polar ring below the membrane system and seem to fuse with the region where only the plasma membrane exists [15, 53]. They are the first ones to secrete their protein content, which is essential for the protozoan motion and its association with the membrane of the host cell [41]. The micronemal proteins are named MICs [54]. They include proteins with perforin-like properties, adhesins, and serine proteases (subtilisins) [39, 55]. Some of the micronemal proteins are involved in the assembly (together with rhoptry proteins) of the moving junction [41, 56, 57]. Morphological analysis has shown that the number of micronemes is higher in sporozoites, lower in bradyzoites, and intermediate in tachyzoites [3, 42]. Their secretion can be induced by treatments that increase the intracellular calcium concentration [50, 58, 59].

Rhoptries comprise the second group of apical secretory organelles. They are larger than the micronemes, are club-shaped with two well-defined regions (Fig. 3, Additional file 1: Figures S1, Additional file 2: Figure S2, Additional file 3: Figure S3, Additional file 6: Figure S6). The most basal one is wider and gives a spongy appearance, containing proteins involved in the subversion of host cell functions, known as ROPs. The anterior portion, or the neck, concentrates proteins associated with host cell invasion and are known as RONs. They present an acidic pH. Rhoptry secretion plays an essential role in the constitution of the moving junction for *T. gondii* invasion and formation of the parasitophorous vacuole (PV) membrane [60, 61].

The third group of secretory organelles are the dense granules (Additional file 6: Figure S6). They are usually spherical with a mean diameter of 0.2 μm and are distributed throughout the protozoan body. They also contain a large number of proteins secreted at the lateral and posterior portion of the protozoan when it is localized within the PV. The dense granule proteins are known as GRAs and are involved in the assembly of a network of tubules and filamentous structures with the PV. Their number is higher in sporozoites [14, 15, 34, 62].

The nucleus of the protozoan is localized in the middle portion of the cell body. It is roughly spherical, with a discrete concavity at its upper side (i.e., the one that faces the apical complex) where the Golgi complex accommodates. During division, the nucleus assumes a horseshoe shape, maintaining the integrity of its membrane [63, 64].

Above the nucleus, the apicoplast is localized at a lateral position (Fig. 3, Additional file 2: Figure S2, Additional file 3: Figure S3 and Additional file 7: Figure S7). The apicoplast is elongated and delimited by four membranes. It is an organelle of endosymbiotic origin and is closely associated with the Golgi complex and the endoplasmic reticulum. The apicoplast contains an extra chromosomal 35 kb DNA and the whole machinery that allows the synthesis of some proteins. It plays essential roles in biochemical processes such as type II fatty acid synthesis and isoprenoid and Hemi group synthesis and is the first organelle to divide during the protozoan cell cycle [64–66].

*Toxoplasma gondii* presents profiles of the rough and smooth endoplasmic reticulum distributed throughout the cell. The Golgi complex is closely apposed to the concave (anterior) portion of the nucleus, displaying 4 to 6 lamellae. It divides at an early phase of the endodyogeny, and each complex is incorporated into one of the new cells formed [64, 67].

*Toxoplasma gondii* presents a single and ramified mitochondrion that can reach about 10 µm long distributed throughout the cell [68, 69] (Fig. 3).

*Toxoplasma gondii* also contains about ten acidocalcisomes, which are acidic organelles that store calcium and are involved in intracellular homeostasis and osmoregulation (Fig. 3). The diameters of these organelles vary from 40 to 150 nm and are dispersed in the cytoplasm [70].

Other cytoplasmic structures of *T. gondii* include lipid bodies, which seem to be more abundant in sporozoites (Additional file 8: Figure S8), and amylopectin granules (Additional file 9: Figure S9, Additional file 10: Figure S10, Additional file 11: Figure S11), which are rarely seen in tachyzoites, but characteristically found in bradyzoites (Additional file 9: Figure S9) and sporozoites. They appear as spherical structures that contain reserve polysaccharides [3].

Morphologically, bradyzoites and sporozoites differ from tachyzoites in several aspects, such as the position of the nucleus, that is more posterior in them; the number of micronemes, dense granules and amylopectin granules, as well as the aspect of the rhoptries (Additional files 8 and 9: Figures S8 and S9) [42].

#### The life-cycle of T. gondii in the definitive host: schizonts, gametes and gametogenesis

**Fig. 4:**
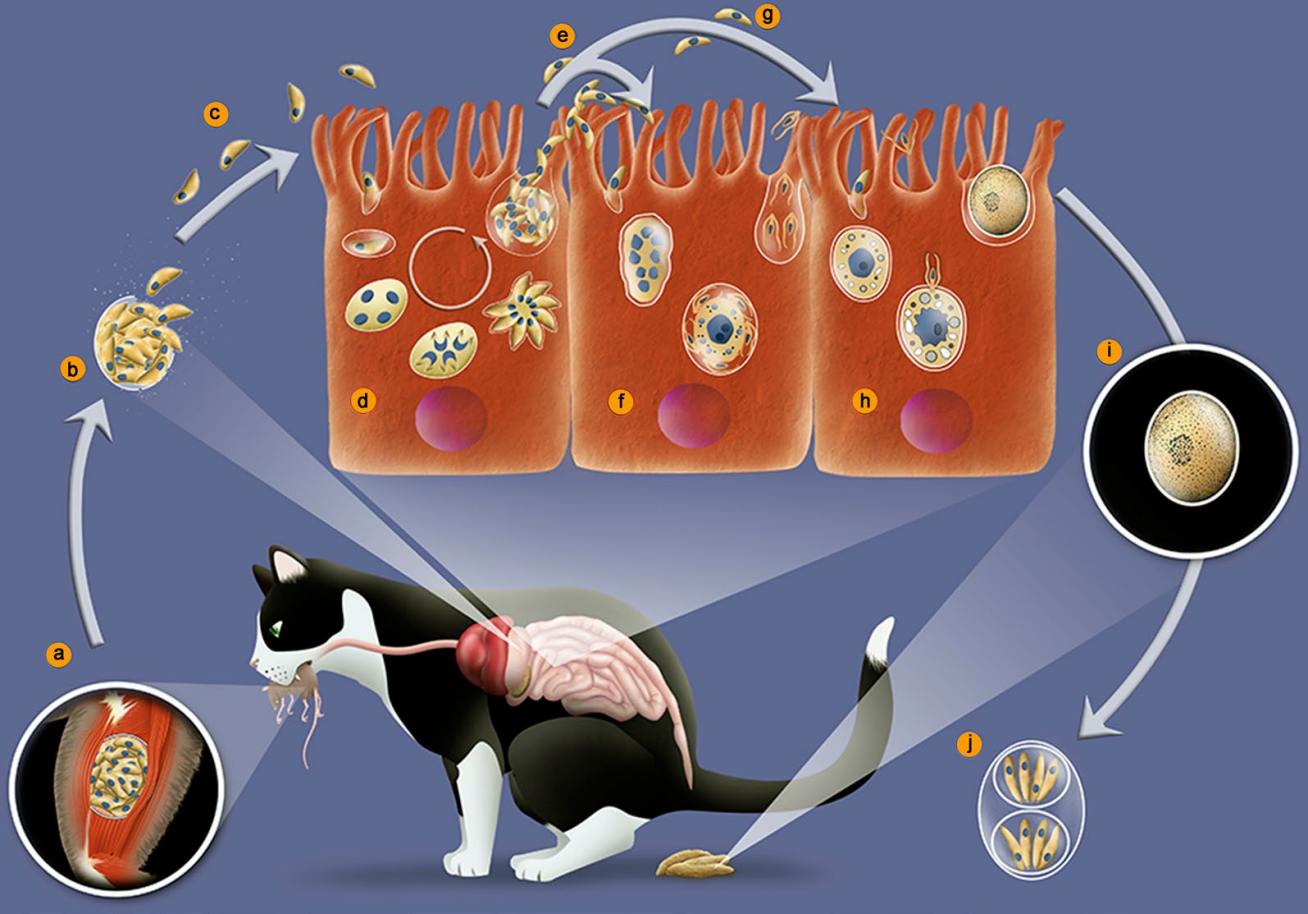
Life-cycle of *Toxoplasma gondii* in cat. **a** Ingestion of prey containing tissue cysts. **b** The cyst wall is digested in the stomach and intestines, liberating bradyzoites. **c** Bradyzoites invade epitelial cells of the intestine. **d** In the enterocytes bradyzoites divide by schizogony giving rise to merozoites. **e** Merozoites differentiate into microgamonts, or macrogametes (**f**). **g** Fertilization gives rise to an unsporulated oocyst excreted with cat feces (**h**). **i** Sporulation occurs and generates two sporocysts with four sporozoites each (**j**)

As previously mentioned, the sexual cycle of *T. gondii* only takes place in members of the Felidae family. Most of the studies on this part of the cycle have been carried out in cats, especially in young kittens [27, 71, 72]. Due to the difficulties in maintaining and sacrifice cats in the laboratory, there are relatively few papers dealing with the cycle of *T. gondii* in this host. The primary process of infection of a feline is by ingesting prey containing tissue cysts, or oocysts containing sporozoites excreted by another feline (Fig. 1). Following ingestion, the wall of the cysts is disrupted in the stomach, probably due to its low pH and the action of proteolytic enzymes, releasing bradyzoites or sporozoites, respectively. In either situation, intestinal epithelial cells will be the first cells to be invaded and will turn into schizonts, stage of asexual reproduction that can be identified by the presence of several nuclei (Fig. 4).

During schizogony, successive nuclear divisions precede the individualization of each cell. After consecutive rounds of nuclear division, the formation of the inner membrane complex for the individualization of each merozoite can be followed in parallel with the apical complex’s appearance, including micronemes and rhoptries, all characteristic structures of a typical Apicomplexa. These cytoplasmic structures organize around each nucleus, giving rise to the daughter cells, called merozoites inside the host cell. Rupture of these enterocytes releases many merozoites, which are also able to infect new enterocytes and divide again by schizogony. Each schizogonic cycle gives rise several merozoites that will be released to readily invade new enterocytes, exponentially enhancing the number of parasites. Five different types of schizonts of *T. gondii* were described in feline intestinal epithelial cells before gametogony begins [73].

**Fig. 5:**
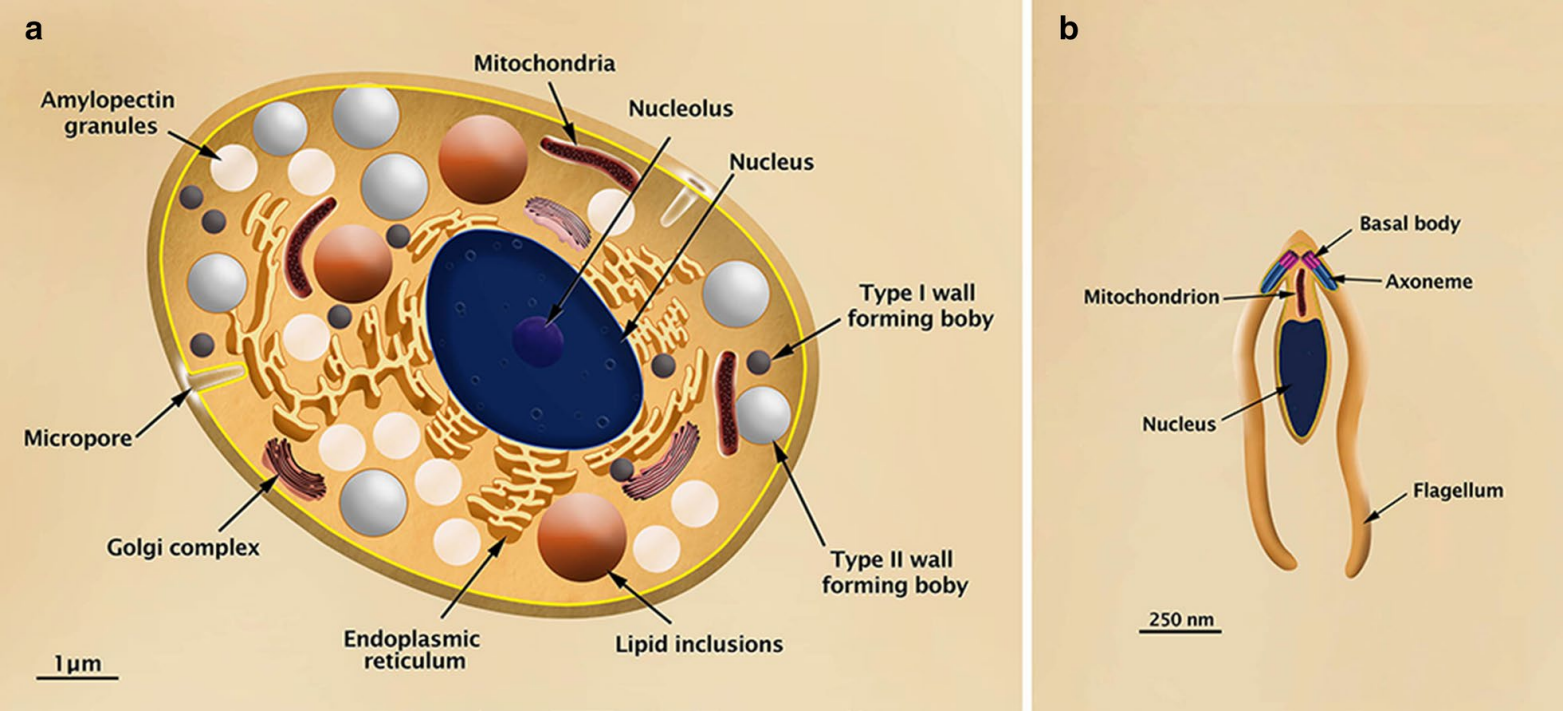
**a** Illustration of the macrogamete of *Toxoplasma gondii* in transversal section showing the main internal structures. **b** Illustration of the microgamete of *Toxoplasma gondii* in longitudinal section

Three to fifteen days after the feline primary infection, the schizonts and merozoites are found mainly in the ileum portion of the intestine, and some begin to differentiate into gametes (Fig. 4). Macrogametes are generated from a single merozoite and display an oval shape (8 µm long and 6 µm wide), a single nucleus, endoplasmic reticulum, mitochondria, lipid bodies, amylopectin inclusions, and wall-forming bodies type I (diameter of 0.35 µm and electron dense) and II (width of 11.2 µm, less dense and in smaller number) (Fig. 5a). Microgametes are formed by a schizogonic division and are elongated (6 µm long and 2 µm wide); they display a condensed nuclear chromatin, two basal bodies, respective flagella that are localized in the anterior region, and a mitochondrion that is restricted to the base of the flagella (Fig. 5b) [10, 62].

The fusion of a microgamete with a macrogamete produces the immature oocyst, which is released into the intestinal lumen of the definitive host. The immature oocyst displays an elliptical shape (13 µm long × 11 µm wide) and contains in its interior a single sporoblast. Once the oocysts are liberated with the feces of the cat, the aerated environment triggers maturation, i.e. sporulation. Upon sporulation, the oocyst divides into two sporocysts (6–8 µm each). A thin and electron dense layer and an inner electron lucent layer as well as two external membranes form the oocyst wall. After the subsequent oocyst maturation, each sporocyst contains four sporozoites. The sporulated oocysts are highly resistant to environmental conditions and remain viable either in water or in dry conditions for several months [3, 62, 74] (Additional files 11: Figures S11, Additional file 12; Figure S12, Additional file 13: Figure S13).

#### The life-cycle of T. gondii in the intermediate host

Intermediate hosts can be infected through several pathways (Fig. 1). Both tissue cyst and oocysts walls are removed by digestive enzymes, liberating, respectively, bradyzoites or sporozoites that inside the new host, move by a unique mechanism of gliding [24, 56]. Micronemal proteins are the first to be secreted and are essential for protozoan motility by gliding and initial adhesion to the host cell surface. Gliding motility results from a complex assembly of proteins anchored to an actin myosin motor localized between the plasma membrane and the inner pellicle. This so-called glideosome involves microneme proteins (AMA1 and MIC2) inserted in the plasma membrane of the tachyzoite that recognize and attach to receptors of the host cell’s plasma membrane. The intracellular domain of AM1 and MIC 2 are connected to an aldolase, that is linked to filamentous actin that is pushed by a TgMYO myosin motor and several GAP proteins (GAP40, GAP45, GAP50 and GAPM) that build a connection between the three membranes of the pellicle and the alveolin network [57]. This complex assembly pushes the tachyzoite forward towards a new host cell.

**Fig. 6:**
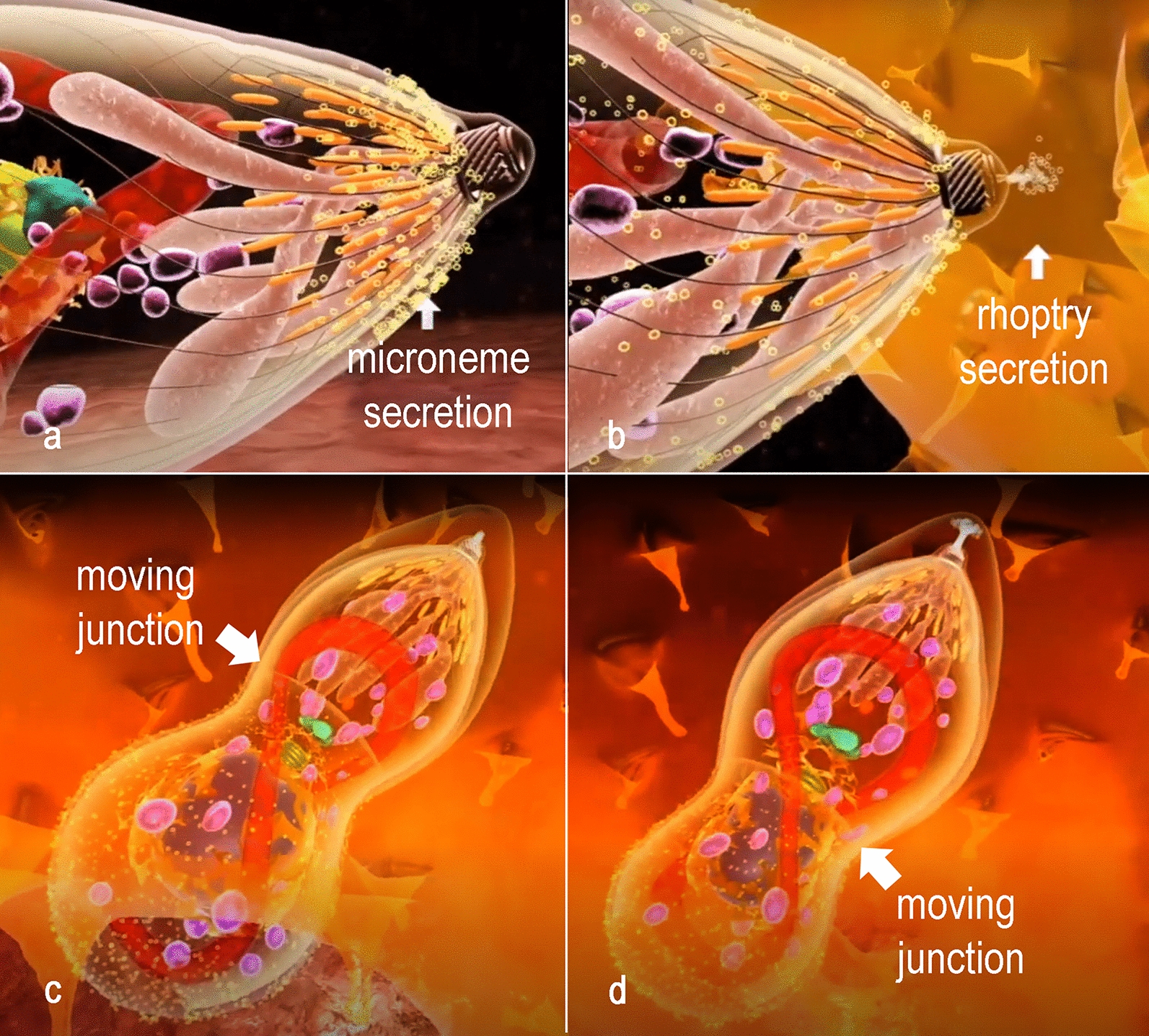
Sequential events of invasion of a host cell. **a** Microneme secretion and conoid extrusion are part of the gliding machinery. **b** The parasite attaches to the host cell membrane and secretion of proteins of the rhoptries neck induces host cell deformation. **c**, **d** A moving junction is established between the host cell and the parasite membrane at the point of invasion. Progressively the parasite invades the host cell protected in the PV, from which host cell proteins are excluded, as well as proteins of the gliding machinery

The interaction of *T. gondii* with a cell from the host aims, ultimately, its internalization. In *T. gondii*, this process can occur in any nucleated cell, particularly macrophages, epithelial cells, muscle cells, and neurons. Initially, *T. gondii* attaches with the surface of the potential host cell and in sequence reorients the apical side inducing, by secretion of proteins localized in the apical organelles, i.e. micronemes and rhoptries, the process of internalization (Fig. 6, Additional file 14: Figure S14 and Additional file 15: Figure S15). For invasion, the tachyzoite assembles a moving junction with the plasma membrane of the host cell (Fig. 6, Additional file 15: Figure S15). This moving junction forms a ring around the tachyzoite at the point of entry into the host cell. It results in the attachment of the micronemal protein AMA1 inserted in the surface of the tachyzoite to the rhoptry protein RON 2 that is secreted, inserted and exposed at the host cell membrane.

**Fig. 7:**
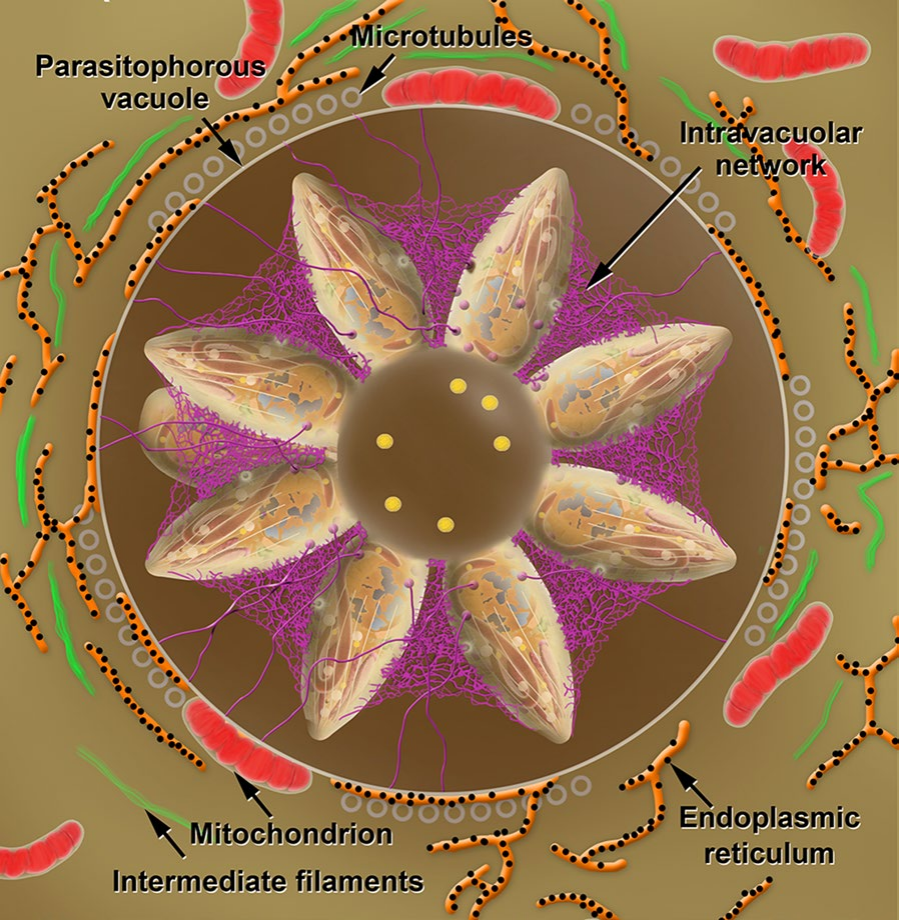
Schematic view of a PV in cross section showing tachyzoites linked to the residual body. Inside the residual body, acidocalcisomes (yellow) are accumulated. An intravacuolar network (magenta) stabilizes the rosette of parasites. Profiles of the endoplasmic reticulum (orange) with ribosomes (black) adhered, microtubules (grey circles), intermediate filaments (green) and mitochondria (red) of the host cell and assemble around the PV

Besides RON 2, the rhoptries also secrete RON 4, RON 5 and RON 8, which connect to RON2 in the cytoplasmic side of the host cell that associate with actin filaments. The tachyzoite squeezes itself along this moving junction, assuming an hourglass shape constricted at the point of contact (Fig. 6c, d). The process of parasite internalization is complex, and structures of the protozoan cytoskeleton (i.e. the conoid) play a role as it moves up and down during the internalization process. In a further step, proteins in the basal portion of the rhoptry are secreted and implement modifications in the host cell behavior and formation of the membrane of the parasitophorous vacuole where the protozoan will survive and multiply (i.e. the PV). Besides, changes will take place in the host cell cytosol. Among these modifications, we point out the inhibition of fusion of host cell lysosomes with the PV membrane. On the other hand, the distribution of other organelles including the mitochondria and the endoplasmic reticulum tend to concentrate around the PV (Fig. 7). In contrast, the PV itself settles near the nucleus [75–77].

**Fig. 8:**
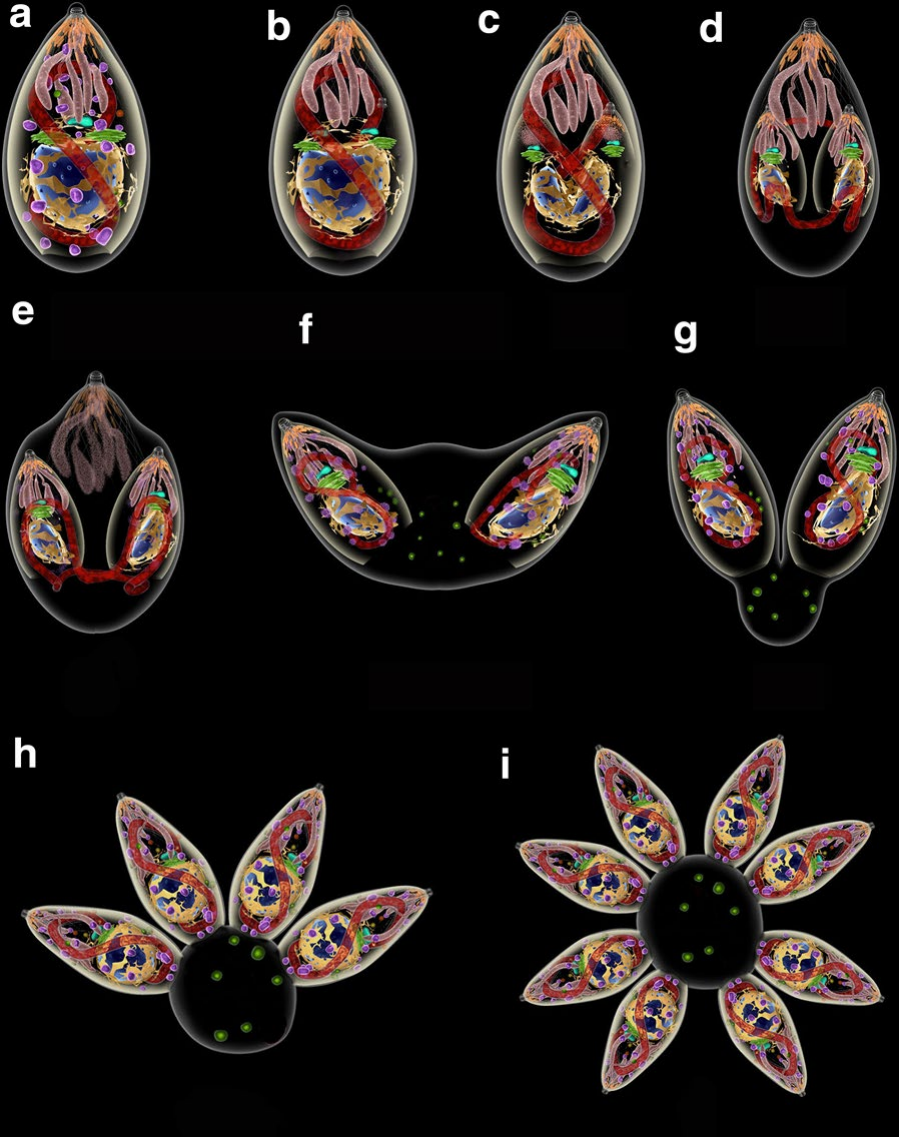
Sequence of events of division by endodiogeny. **a**, **b** The Golgi complex and the apicoplast are the first organelles to divide. **c** The nucleus assumes a horse-shoe shape. Two new apical complexes start to form. **d** The inner pellicle grows and embraces the structures of the daughter cells, including the nucleus. **e** The mitochondrion is the last organelle to be separated between the daughter cells. The apical complex of the mother cell is still maintained at this point. **f** The two daughter cells emerge and the outer membrane of the mother cell is incorporated. The apical complex of the mother cell disappears. **g** The two daughter cells remain linked to the residual body where acidocalcisomes (green) start to accumulate. **h** The process is repeated until a rosette of parasites is formed (**i**)

Inside the PV, secretion of proteins localized in the rhoptries and in dense granules occur, inducing modification of the PV membrane and the assembly of a network of tubular and filamentous structures within the PV (Fig. 7, Additional file 16: Figure S16). The tachyzoite starts a process of division by endodyogeny, where two daughter cells are assembled inside the mother cell. Successive divisions begin to create an organization of tachyzoites known as rosettes (Figs. 7, 8).

After successive division cycles, some stimuli (e.g. a Ca^++^ influx) induce the egress of the tachyzoites and rupture the membrane of the host cell, liberating these new tachyzoites that can infect new host cells in the extracellular space (Additional file 17: Figure S17) [50].

Within a few days of infection, tachyzoites localized within a PV gradually begin to change their metabolism, slowing the division rate and converting into bradyzoites (Additional file 18: Figure S18). Cysts are more numerous in muscle cells and neurons than in other cell types. Under certain conditions including immunodepression, the bradyzoites can reconvert into tachyzoites.

Bradyzoites reside in tissue cysts. The size of each cyst varies according to age, parasite strain and nature of the host cell. Small cysts have a diameter of around 5 µm, while old cysts can reach 60 μm, and may contain about 2000 bradyzoites (Additional file 19: Figure S19 and Additional file 20: Figure S20).

**Fig. 9:**
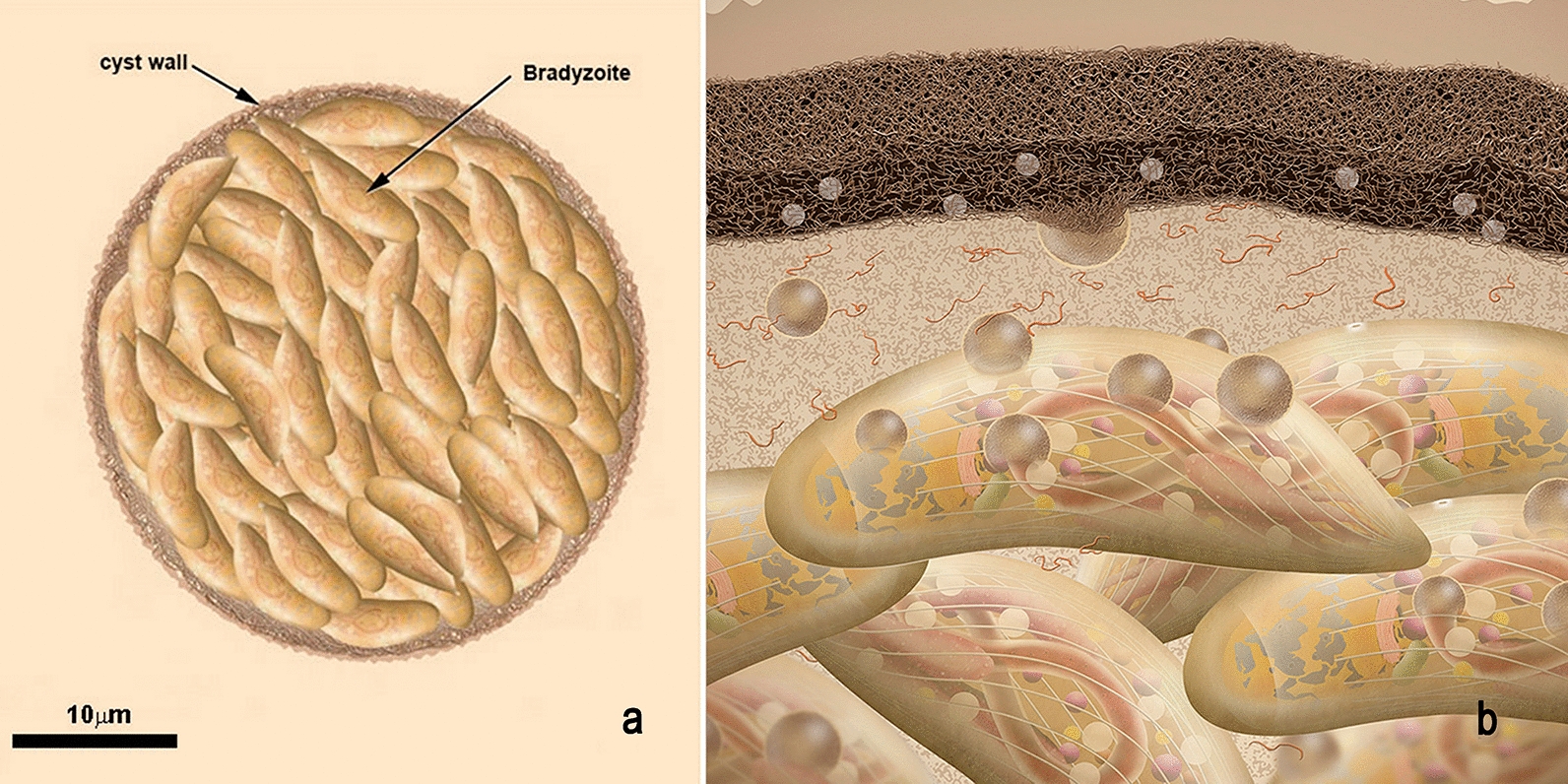
Scheme of a tissue cyst of *Toxoplasma gondii*. **a** The cyst wall is thick and filamentous. Each cyst may contain hundreds of bradyzoites. **b** A zoom view of the tissue cyst. The cyst is surrounded by a membrane and below it a thick cyst wall is deposited. The components of the cyst wall, as well of the cyst matrix, are secreted by the bradyzoites

The bradyzoites also secrete organelle contents into the PV matrix, which gradually lead to the assembly of a cyst wall in association with the PV membrane and an intracystic network, as shown in Fig. 9a, b. The cyst wall is usually thin (< 0.5 µm) and formed by a limiting membrane, which is adjacent to a slightly electron dense matrix, and an inner layer where small vesicles and tubules are seen.

## Animations

### Part I. The cat as definitive host

A sequence of two animations was produced describing the life-cycle of *T. gondii* in cat and man are included as Additional file 21: Video S1 and Additional files 22: Video S2, respectively. That was not a simple task, since there are many *T. gondii* transmission paths between hosts, but also contaminated environment. We have made an option to start with a short introductory text establishing *T. gondii*’s medical importance. The action begins with a stray cat preying on a rat that was infected. In the digestive system of the cat, the bradyzoites are liberated from tissue cysts. Bradyzoites can invade any nucleated cell, just like tachyzoites and sporozoites. Still, enterocytes are probably the most accessible when infection occurs by carnivorism and are henceforth the preferential target cells for invasion. Within hours of ingesting tissue cysts, a series of schizogonic cycles will start in feline enterocytes, producing merozoites. Five morphologically distinct types of schizonts were described [3, 62]. However, the number of schizogonic cycles that will occur before gametogenesis starts is not known. So, for clarity, only one schizogonic cycle is represented in the animation. Another challenge was the representation of the gametogony, both micro and macrogametogenesis, raised many questions and doubts that are still unanswered. A large number of microgametes are produced from a single merozoite. These are flagellated; hence, they are believed to be able to swim to actively find the macrogamete for fertilization. The macrogamete is produced from the differentiation of a single merozoite and is much larger than the microgamete and also immotile. The animation of the process of fertilization event raised several questions. In principle, the microgametes released from a host cell would swim to find a host cell bearing a macrogamete to fertilize. However, how can the microgamete identify an enterocyte containing a macrogamete? Do trial and error make it? More than one PV is frequently found in a single host cell. Therefore, can fertilization occur from micro and macrogametes that originate in the same host cell? These hypotheses are discussed in the animation. Following fertilization, it is known that a considerable number of immature oocysts are released in the lumen of the gut and will further be eliminated with the feces of the cat. Again, we faced another unanswered question: since oocysts (as well as macrogamonts) are immotile and relatively large (10–12 μm), how are they expelled from the host cell? A reasonable hypothesis is that immature oocysts are excreted as the enterocytes are discarded by apoptosis as part of the normally intense renovation of the gut epithelium. The first part of the animation ends at this point, with cat feces containing oocysts being excreted and contaminating water and a vegetable garden.

### Part II. Animation of the interaction with the intermediate host

The second part of the animation starts by describing the sporulation process in the open air. Sporulated oocysts, containing two sporocysts with four sporozoites each, are highly infective when ingested with water of raw food (e.g. lettuce). Here, to avoid confusion and follow a straight forward path to be clearly understood by the audience, the contamination of a new host by the ingestion of tissue cysts in uncooked meat is only mentioned, since it has been shown in the first part of the animation. The breakdown of the oocyst and sporocyst walls during digestion is shown, followed by the invasion of host cells of the gut by the sporozoites. This point is also cloudy, with unclear issues to be defined. The sporozoites are believed to quickly convert to tachyzoites that will spread the parasites to other cells and organs.

A close-up view of the tachyzoite gliding in the extracellular space allows the internal organization of the protozoan to be observed, emphasizing the apical complex and secretion of micronemal proteins involved in gliding. Macrophages can act as efficient Trojan horses, transporting tacgyzoites, and for that reason, this is the model cell used in the animation. In the process of invasion, the secretion of micronemes, the motion of the conoid, the secretion from rhoptries, and the constriction characteristic of the moving junction between the plasma membranes of the tachyzoite and the host cell are emphasized. After invasion, the tachyzoites begin to secrete the dense granules that will contribute to the enlargement of the membrane of the PV and generate a membranous network of tubules to keep the parasites stable inside the PV. The cycles of endodyogeny and the daughter cells’ attachment to the residual body result in the rosette assembly of tachyzoites [78]. Inside the residual body, acidocalcisomes, which are represented by green spheres, accumulate. Upon egress, tachyzoites detach from the residual body and actively move towards the extracellular space, rupturing the host cell. Next, in the final sequence of the animation, the tachyzoites reach the central nervous system where conversion to bradyzoites occurs and tissue cysts develop. It is well known that tissue cysts are also formed in other tissues, as muscle and in the retina. Here this event is represented in a brain cell by a change in the color of materials secreted by the parasites that turn from purple (tachyzoites) to green (bradyzoites). These materials accumulate inside the vacuole and form the cyst wall. Bradyzoites are not organized as rosettes inside the cyst.

At this point there is a link with the first part of the animation, where the brain of the mouse contains tissue cysts, completing the cycle.

## Conclusions

We tried to review the main aspects and establish a simplified sequence of events of a highly complex process: the life-cycle of *T. gondii*. We believe that, besides this text, the videos included in Additional files 21 and 22: Videos SV1 and SV2 and the PowerPoint^®^ presentation of Additional file 23: Slideshow are excellent educational tools to be used by teachers to discuss the several possible *T. gondii* transmission paths and basic information on the biology of the parasite. All these, resources are tools that may be used by teachers both in traditional lectures and also to support discussions after the exhibition of the movies. Access of online media for teaching and learning is a reality of our time and, hopefully, this material will be beneficial for self-learning, or education, either in remote or presential mode.

## Supplementary information

**Additional file 1: Figure S1.** Tachyzoite.

**Additional file 2: Figure S2.** Tachyzoite 3D model.

**Additional file 3: Figure S3.** Tachyzoite. 3D model.

**Additional file 4: Figure S4.***T. gondii* cytoskeleton: Conoid (black arrow), polar ring (white arrow), subpellicular microtubules (arrowheads).

**Additional file 5: Figure S5.** Subpelicular network and microtubules. [47].

**Additional file 6: Figure S6.** Tachyzoite. *Abbreviations*: c, conoid; R, rhoptry; A, acidocalcisome; m, microneme; DG, dense granule.

**Additional file 7: Figure S7.** Four Membranes of apicoplast (arrows). Inset, relative position of the apicoplast (A) to the nucleus (N) and Golgi complex (GC). (Image courtesy Dr. Erica Martins Duarte).

**Additional file 8: Figure S8.** Scheme of sporozoite.

**Additional file 9: Figure S9.** Scheme of bradyzoite.

**Additional file 10: Figure S10**. Bradyzoite. Amylopectin granules (arrow).

**Additional file 11: Figure S11.** Sporocyst suture of curved plates (arrowheads).

**Additional file 12: Figure S12.** 3D scheme of a sporulated oocyst containing two sporocysts with 4 sporozoites each.

**Additional file 13: Figure S13.** Section view of a sporulated oocyst.

**Additional file 14: Figure S14.** Tachyzoite (purple) adhered to a lymphocyte (beige) [17].

**Additional file 15: Figure S15.** Tachyzoite (purple) invading a macrophage (beige).

**Additional file 16: Figure S16.** Parasitophorous vacuole: rosette of tachyzoites (purple), filamentous network (pink). Host cell (beige)

**Additional file 17: Figure S17.** Sequence of intracellular cycle. **a** Adhesion, secretion of ropthries. **b** Moving junction: *T. gondii* assumes an hourglass shape. **c** Secretion of dense granules inside the parasitophorous vacuole. **d** Division, formation of the intravacuolar network, accumulation of acidocalcisomes (green) in the residual body. **e** Rosette of parasites. **f** Individualization and egress of parasites.

**Additional file 18: Figure S18.** Cystogenesis. **a** Invasion. **b** establishment of parasitophorous vacuole. **c** Division. **d** Bradyzoite secretion. **e**, **f** Cyst wall thickens, bradyzoites continue to divide. **g** The cyst inside the host cell.

**Additional file 19: Figure S19.** Tissue cyst in the brain of a mouse. Bradyzoites (purple) surrounded by a thick cyst wall (yellow). Blood vessel (red).

**Additional file 20: Figure S20.** Tissue cyst. A cystwall (arrowhead). Amylopectin granules (asterisk), granular matrix (black star).

**Additional file****21****: Video S1.** Part 1- Life cycle of *T. gondii* in the feline host.

**Additional file****22****: Video S2.** Part 2- Life cycle of *T. gondii* in the human intermediate host.

**Additional file 23.** Slide show of *T. gondii* biological cycle, developmental stages and main organelles.

## Data Availability

Not applicable.

## Abbreviations

GRA: Dense granule protein
PV: Parasitophorous vacuole
