# Supplementary figures and images for: The life-cycle of Toxoplasma gondii reviewed using animations
Source: Parasit Vectors. 2020 Nov 23;13:588. doi: 10.1186/s13071-020-04445-z (PMC7686686; doi:10.1186/s13071-020-04445-z)

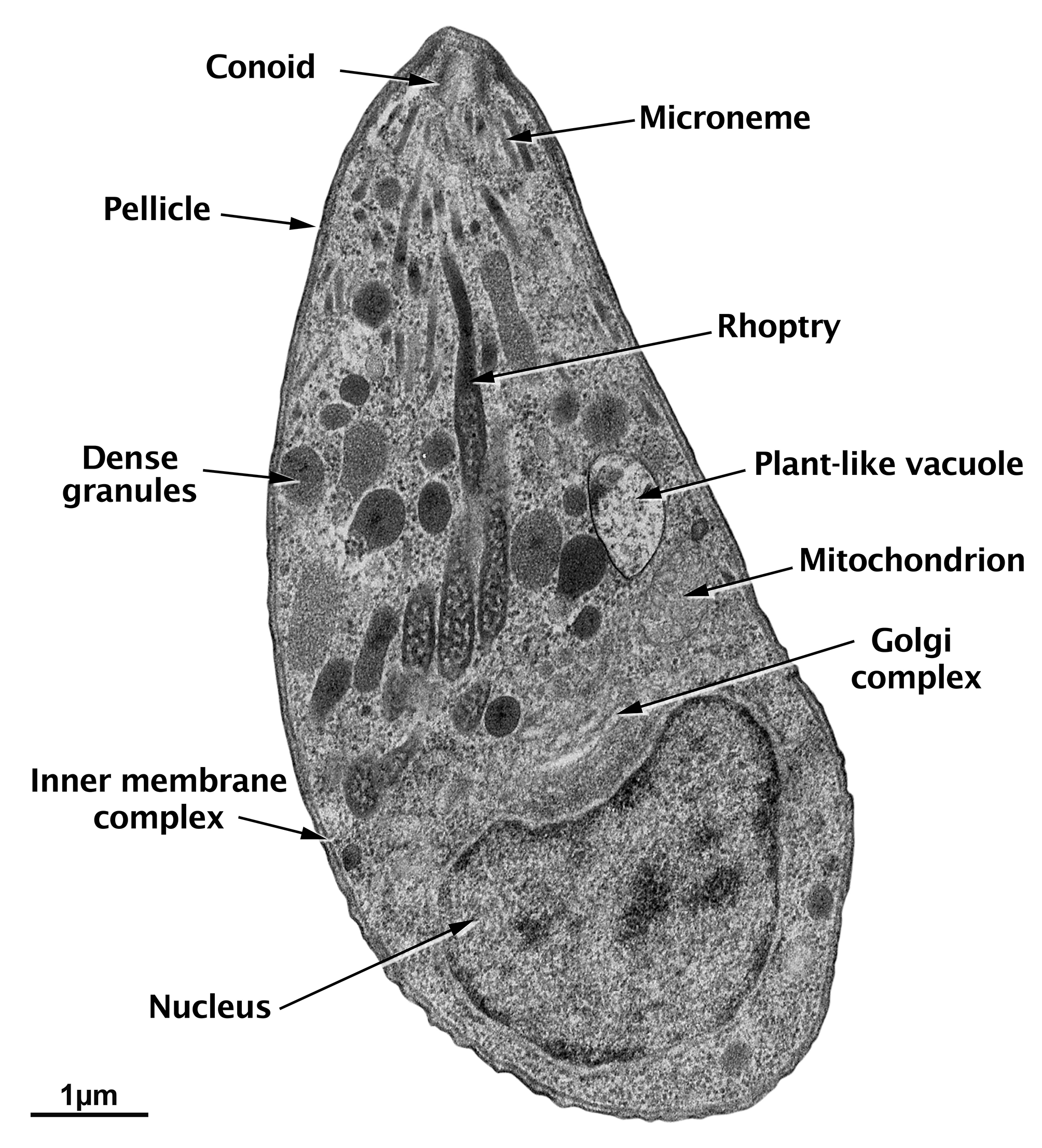

Supplement: Supplementary file 1 — Additional file 1: Figure S1. Tachyzoite. [file 13071_2020_4445_MOESM1_ESM.tif]

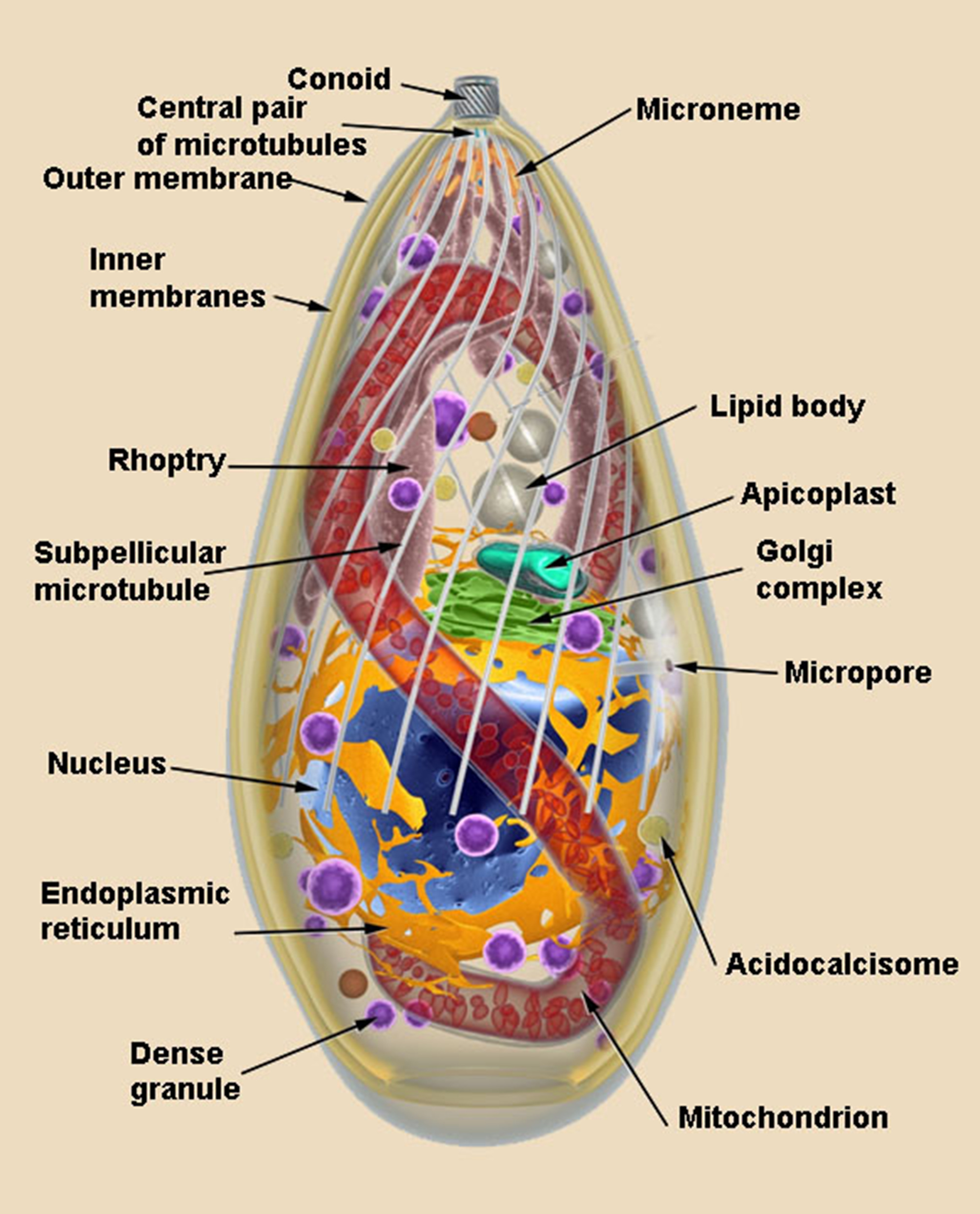

Supplement: Supplementary file 2 — Additional file 2: Figure S2. Tachyzoite 3D model. [file 13071_2020_4445_MOESM2_ESM.tif]

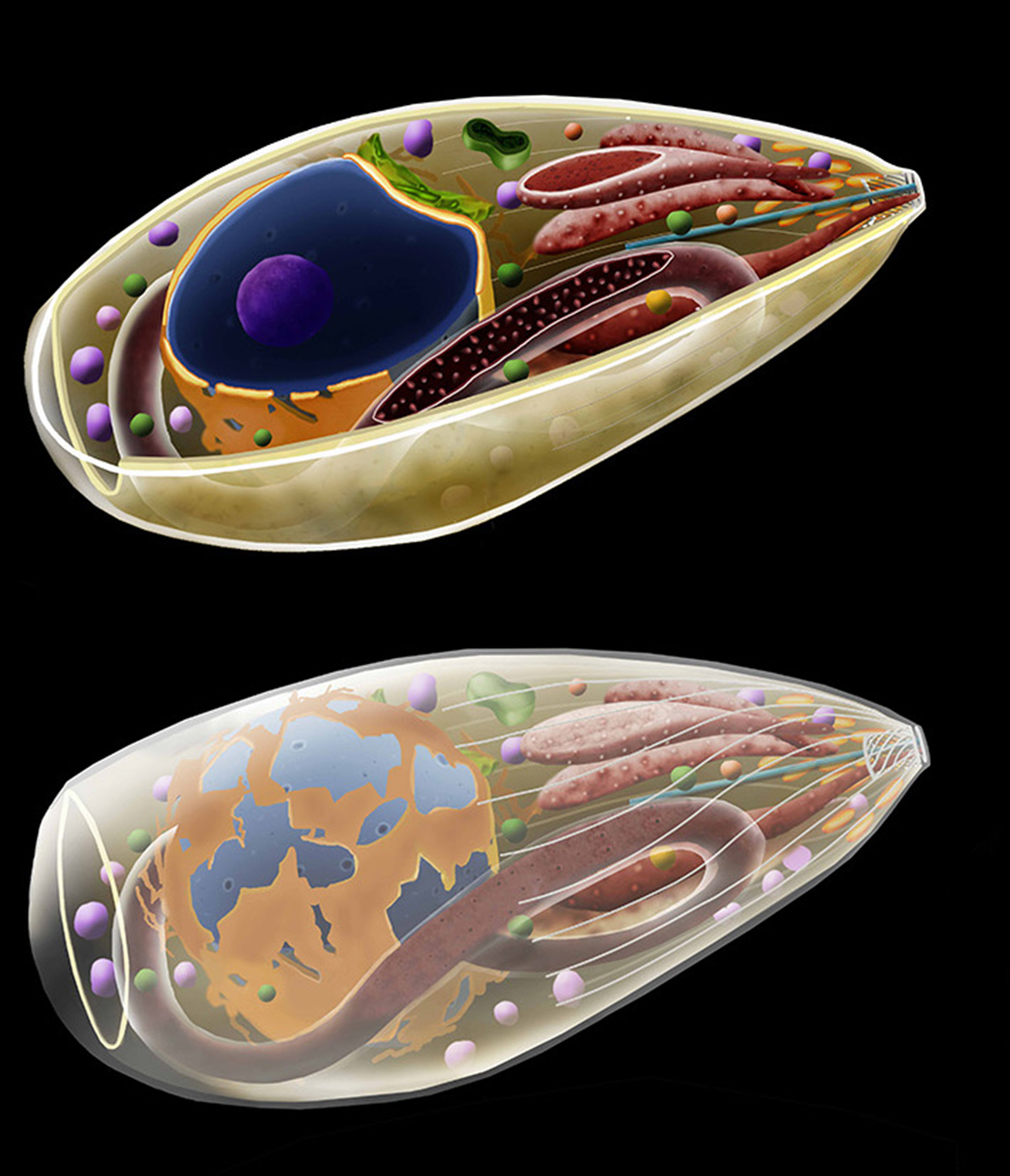

Supplement: Supplementary file 3 — Additional file 3: Figure S3. Tachyzoite. 3D model. [file 13071_2020_4445_MOESM3_ESM.tif]

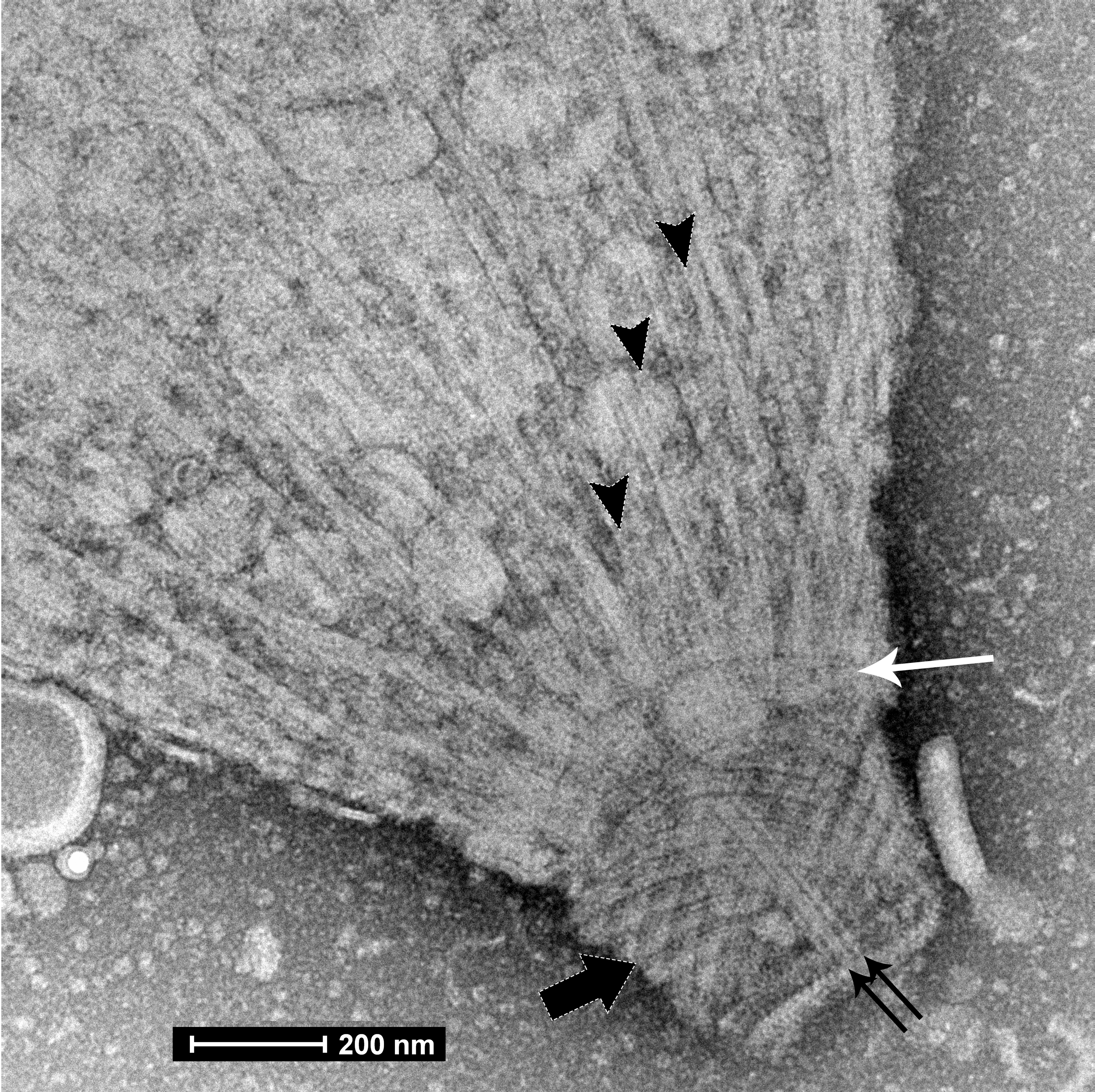

Supplement: Supplementary file 4 — Additional file 4: Figure S4.T. gondii cytoskeleton: Conoid (black arrow), polar ring (white arrow), subpellicular microtubules (arrowheads). [file 13071_2020_4445_MOESM4_ESM.tif]

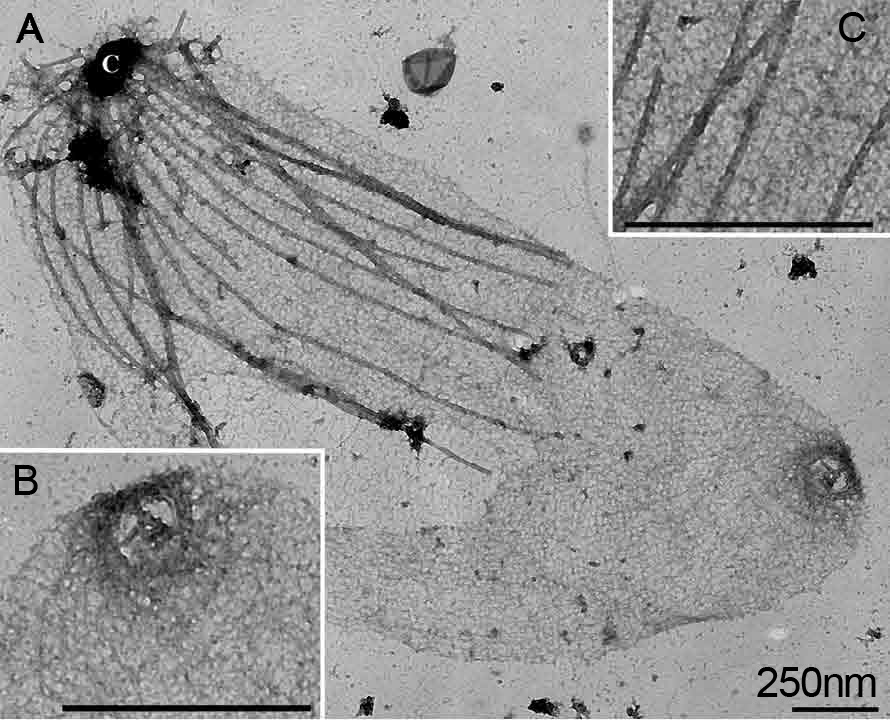

Supplement: Supplementary file 5 — Additional file 5: Figure S5. Subpelicular network and microtubules. [47]. [file 13071_2020_4445_MOESM5_ESM.tif]

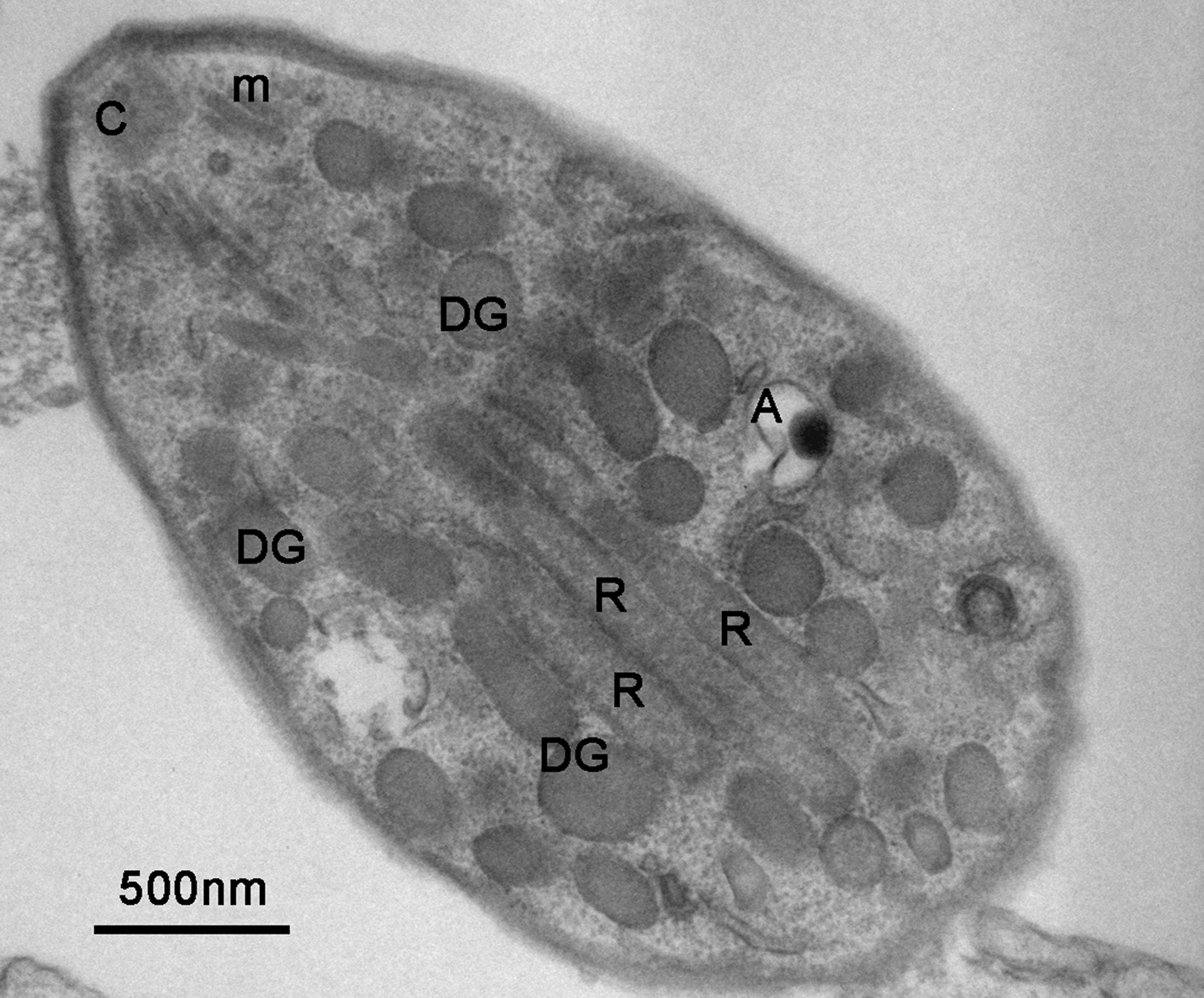

Supplement: Supplementary file 6 — Additional file 6: Figure S6. Tachyzoite. Abbreviations: c, conoid; R, rhoptry; A, acidocalcisome; m, microneme; DG, dense granule. [file 13071_2020_4445_MOESM6_ESM.tif]

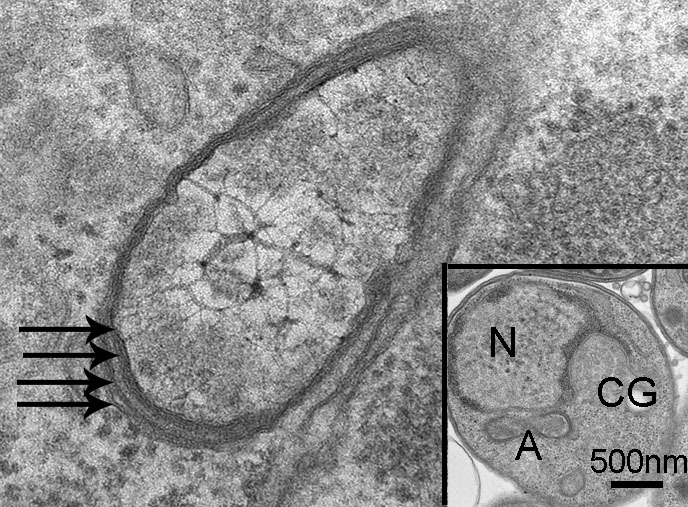

Supplement: Supplementary file 7 — Additional file 7: Figure S7. Four Membranes of apicoplast (arrows). Inset, relative position of the apicoplast (A) to the nucleus (N) and Golgi complex (GC). (Image courtesy Dr. Erica Martins Duarte). [file 13071_2020_4445_MOESM7_ESM.tif]

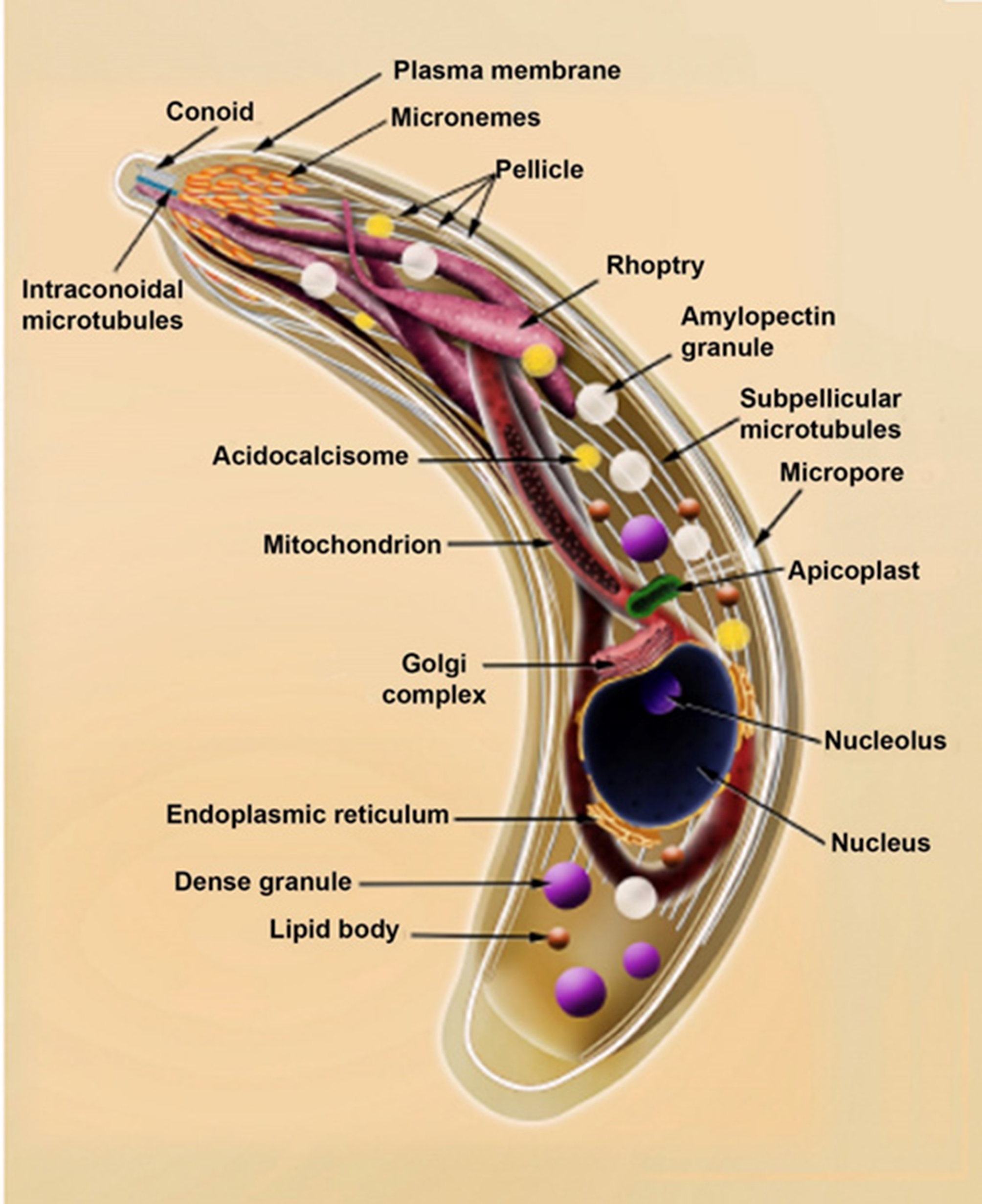

Supplement: Supplementary file 8 — Additional file 8: Figure S8. Scheme of sporozoite. [file 13071_2020_4445_MOESM8_ESM.tif]

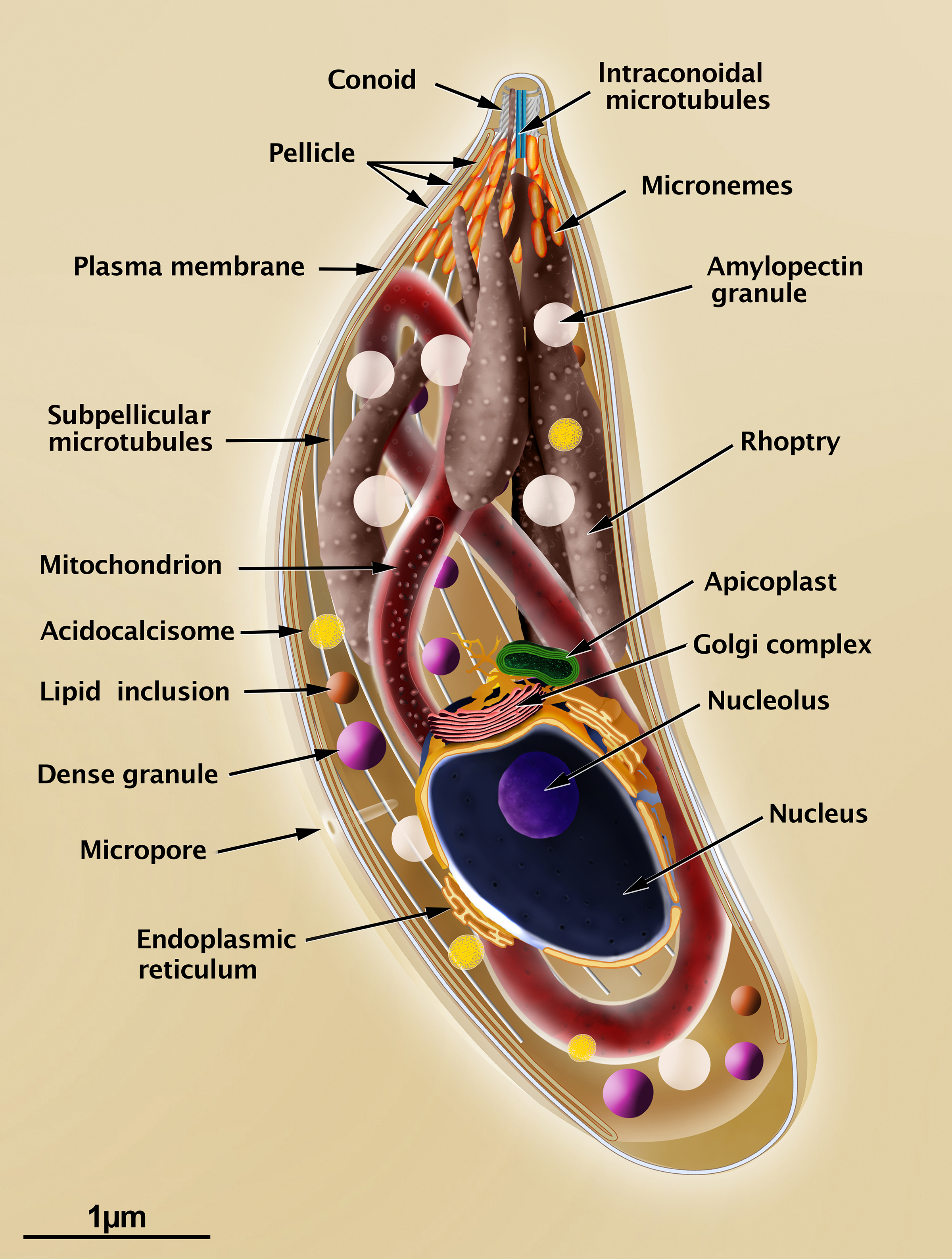

Supplement: Supplementary file 9 — Additional file 9: Figure S9. Scheme of bradyzoite. [file 13071_2020_4445_MOESM9_ESM.tif]

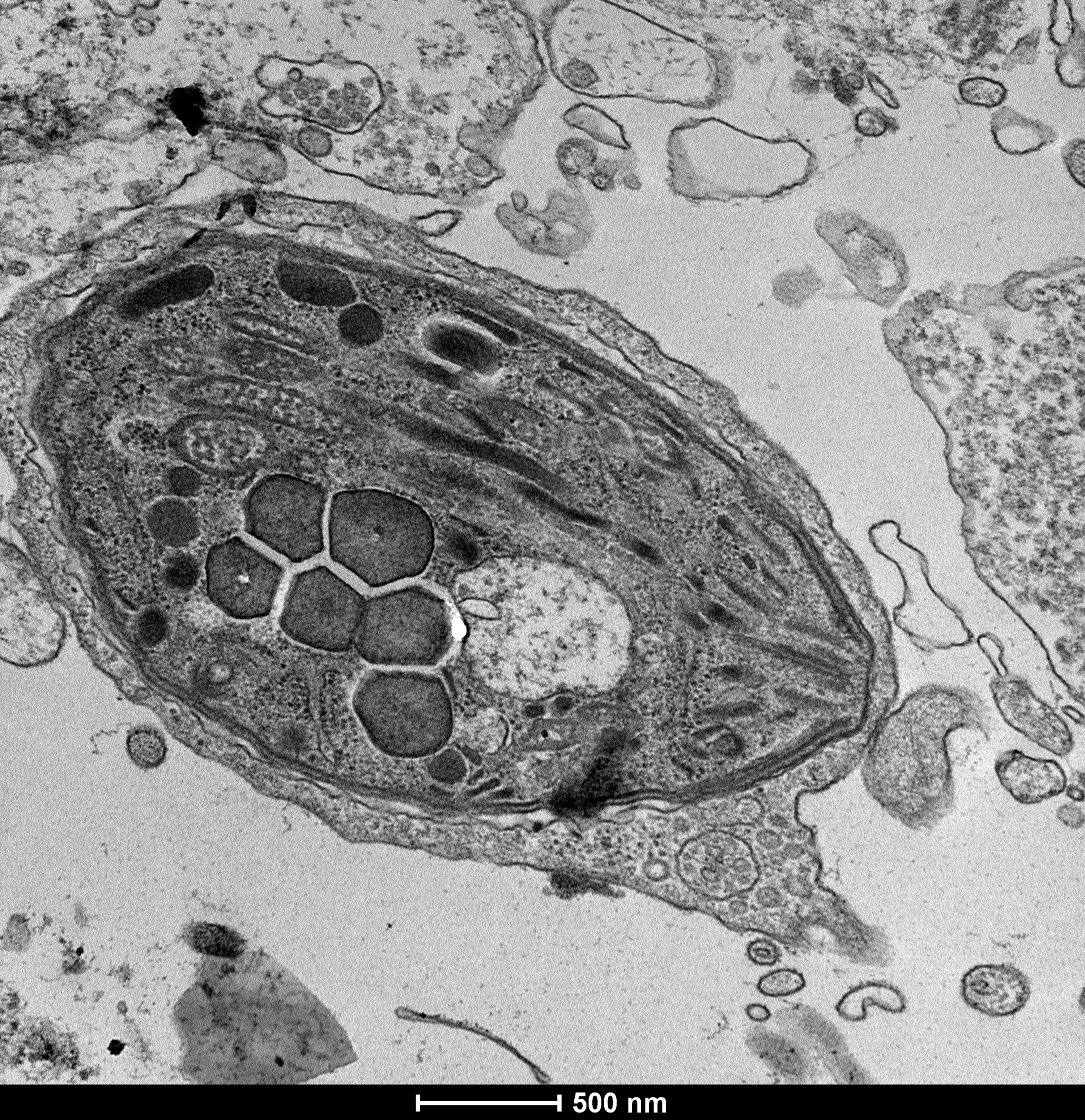

Supplement: Supplementary file 10 — Additional file 10: Figure S10. Bradyzoite. Amylopectin granules (arrow). [file 13071_2020_4445_MOESM10_ESM.tif]

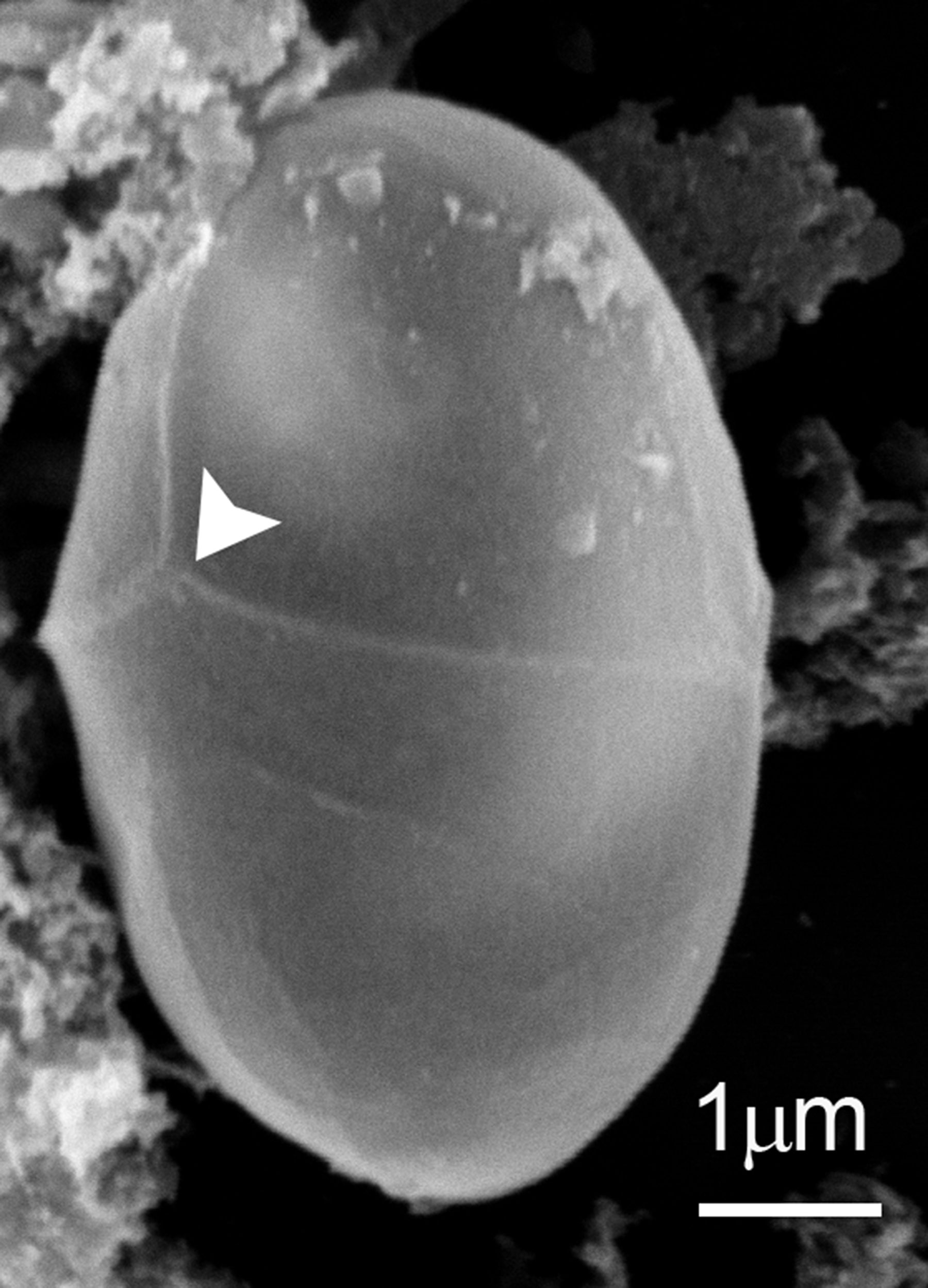

Supplement: Supplementary file 11 — Additional file 11: Figure S11. Sporocyst suture of curved plates (arrowheads). [file 13071_2020_4445_MOESM11_ESM.tif]

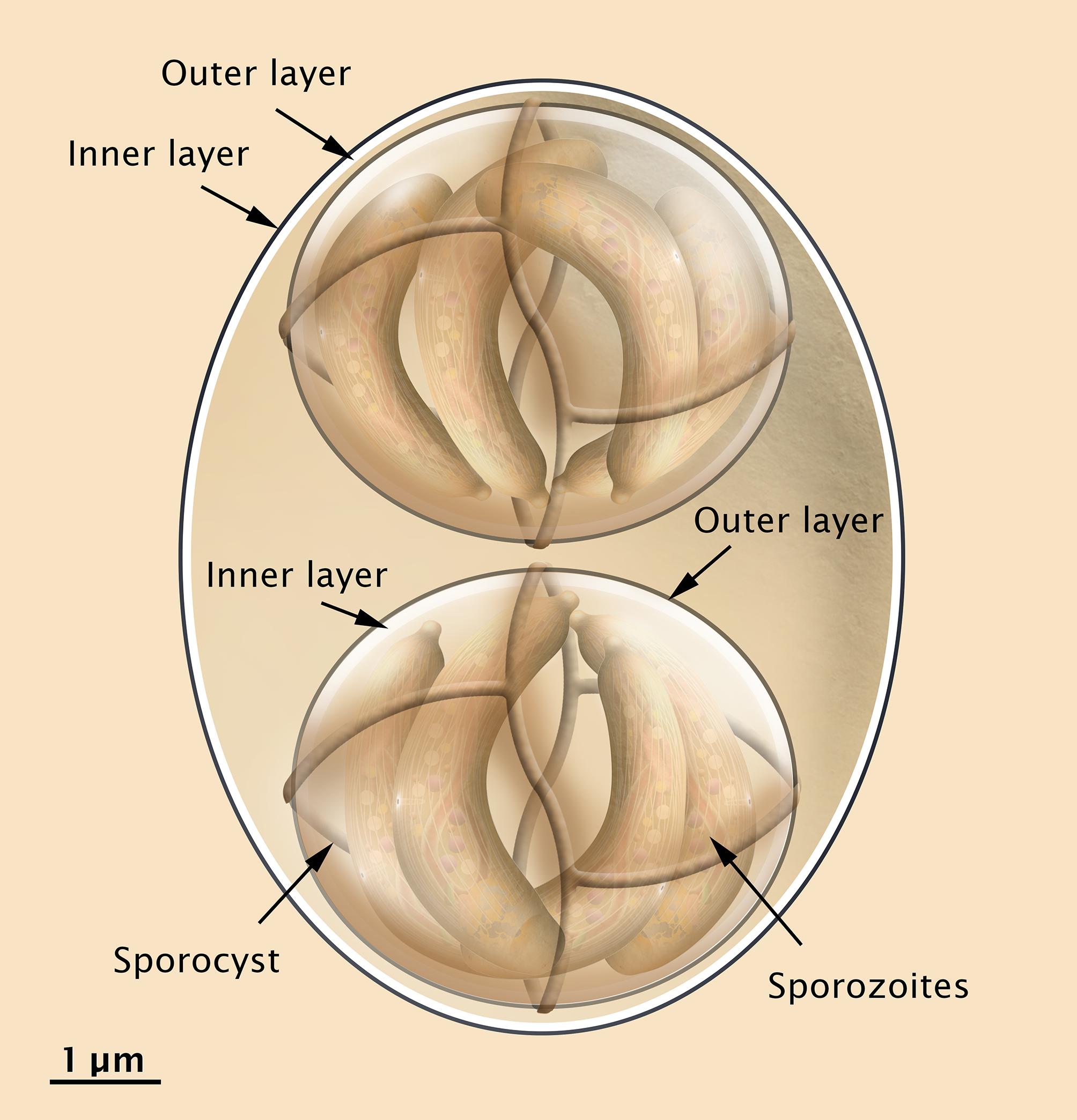

Supplement: Supplementary file 12 — Additional file 12: Figure S12. 3D scheme of a sporulated oocyst containing two sporocysts with 4 sporozoites each. [file 13071_2020_4445_MOESM12_ESM.tif]

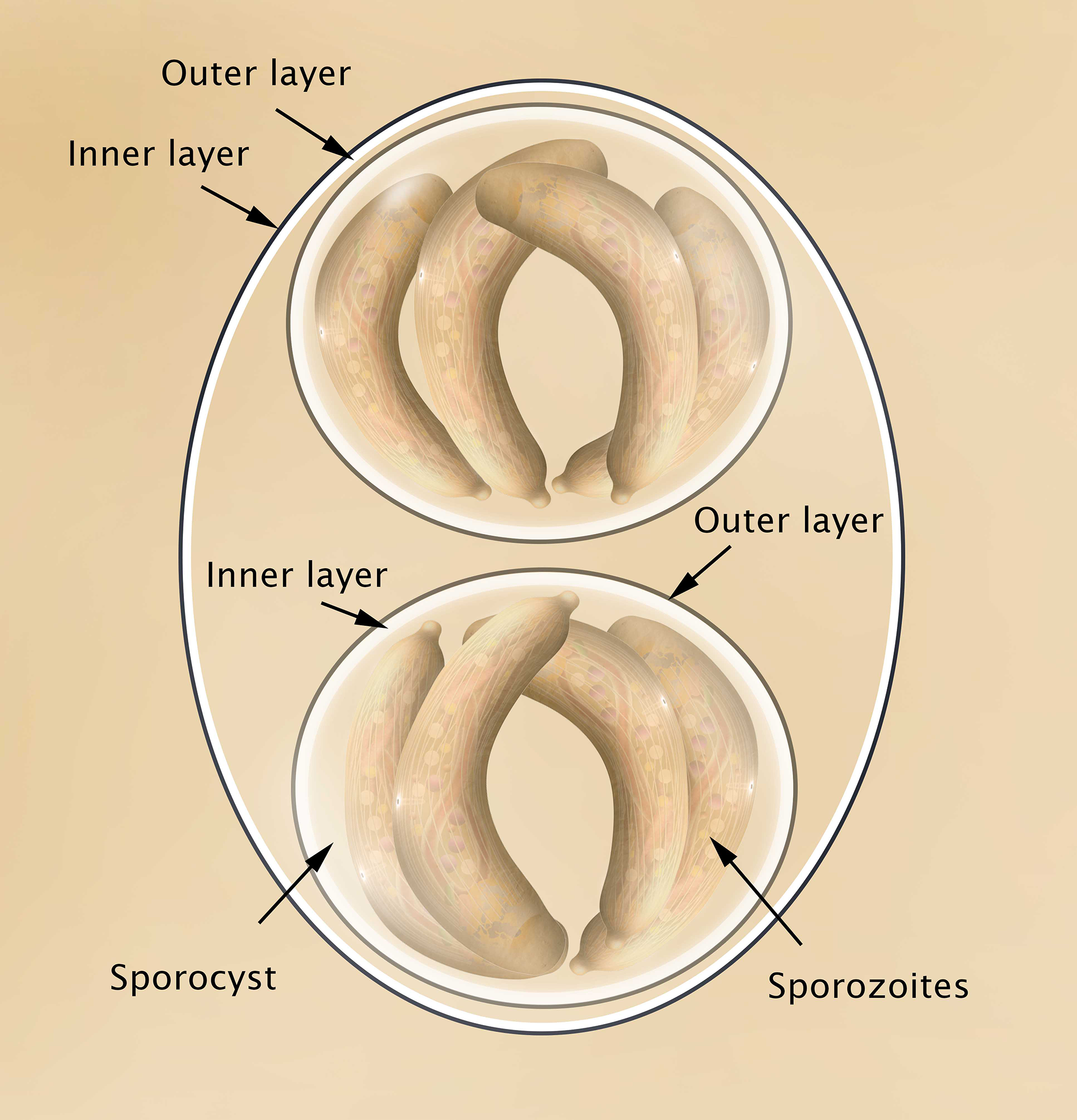

Supplement: Supplementary file 13 — Additional file 13: Figure S13. Section view of a sporulated oocyst. [file 13071_2020_4445_MOESM13_ESM.tif]

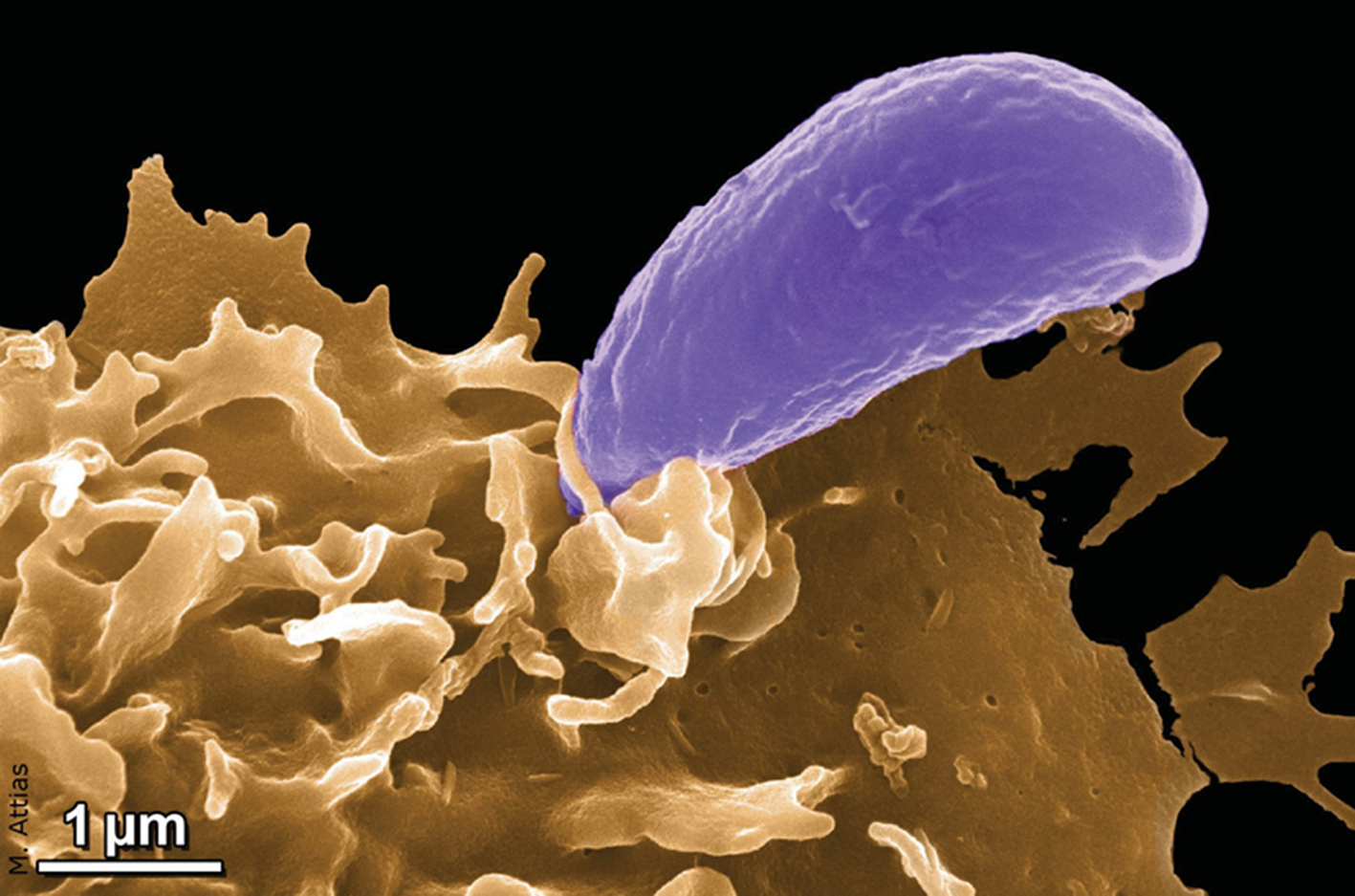

Supplement: Supplementary file 14 — Additional file 14: Figure S14. Tachyzoite (purple) adhered to a lymphocyte (beige) [17]. [file 13071_2020_4445_MOESM14_ESM.tif]

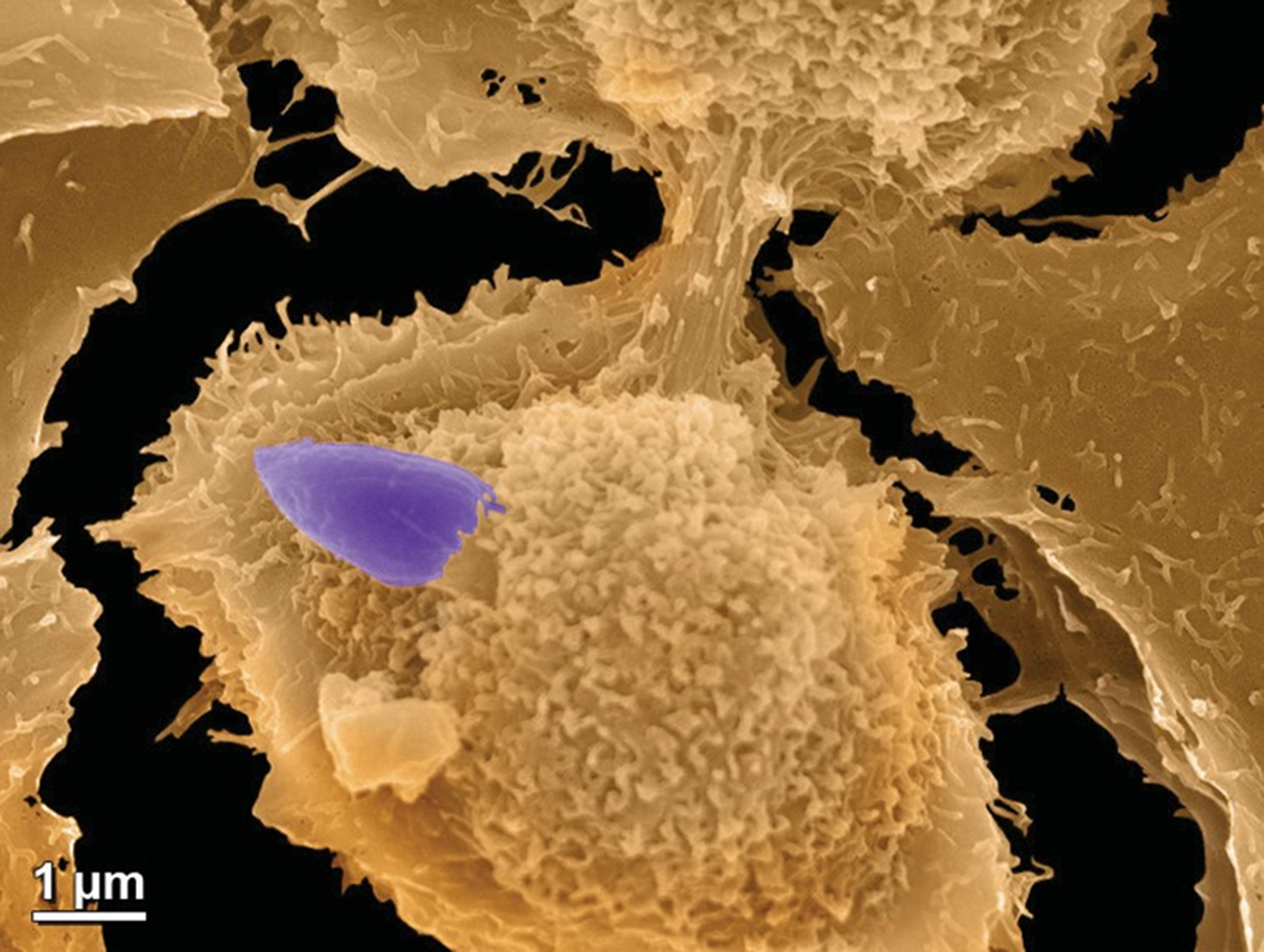

Supplement: Supplementary file 15 — Additional file 15: Figure S15. Tachyzoite (purple) invading a macrophage (beige). [file 13071_2020_4445_MOESM15_ESM.tif]

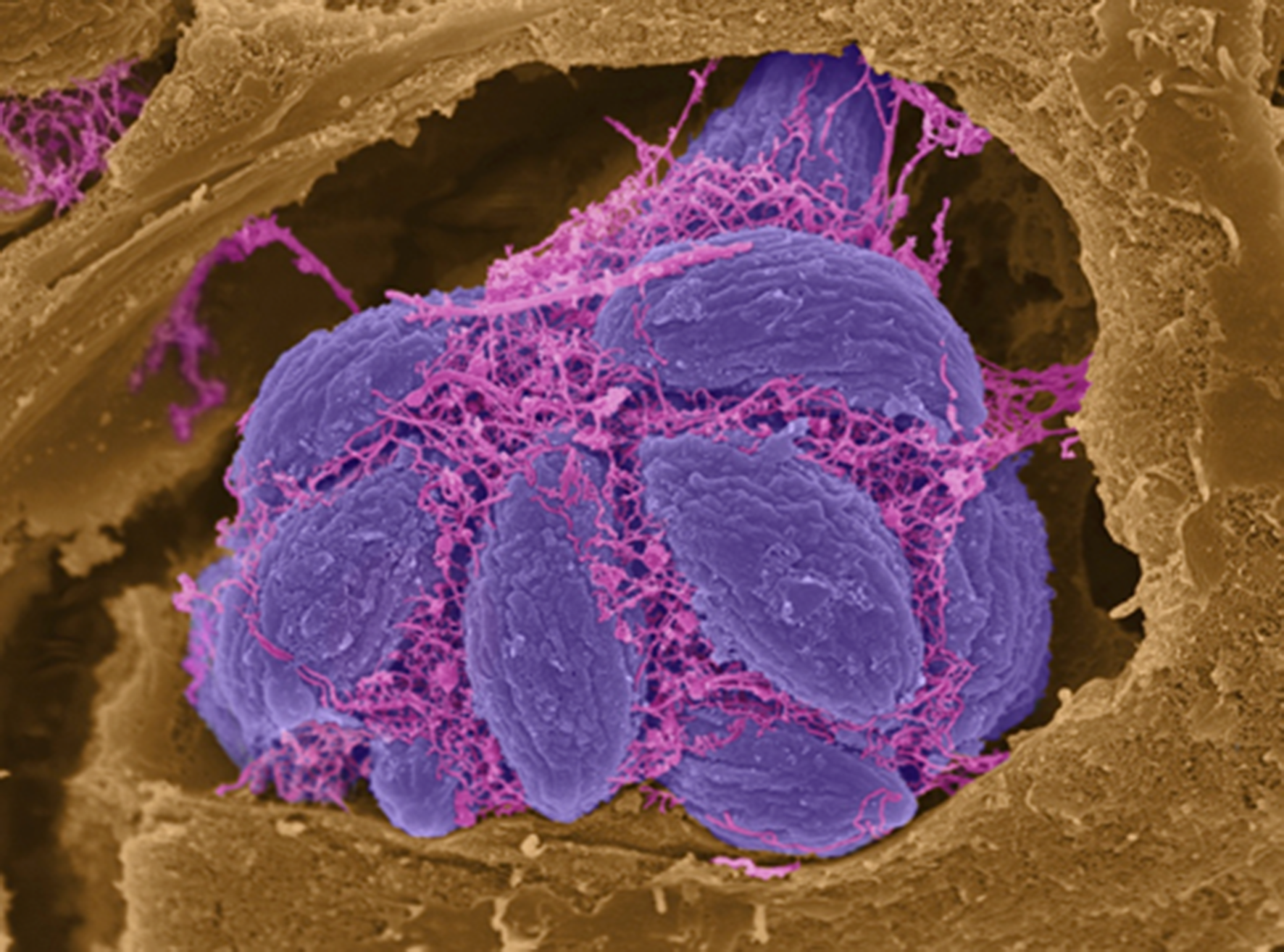

Supplement: Supplementary file 16 — Additional file 16: Figure S16. Parasitophorous vacuole: rosette of tachyzoites (purple), filamentous network (pink). Host cell (beige) [file 13071_2020_4445_MOESM16_ESM.tif]

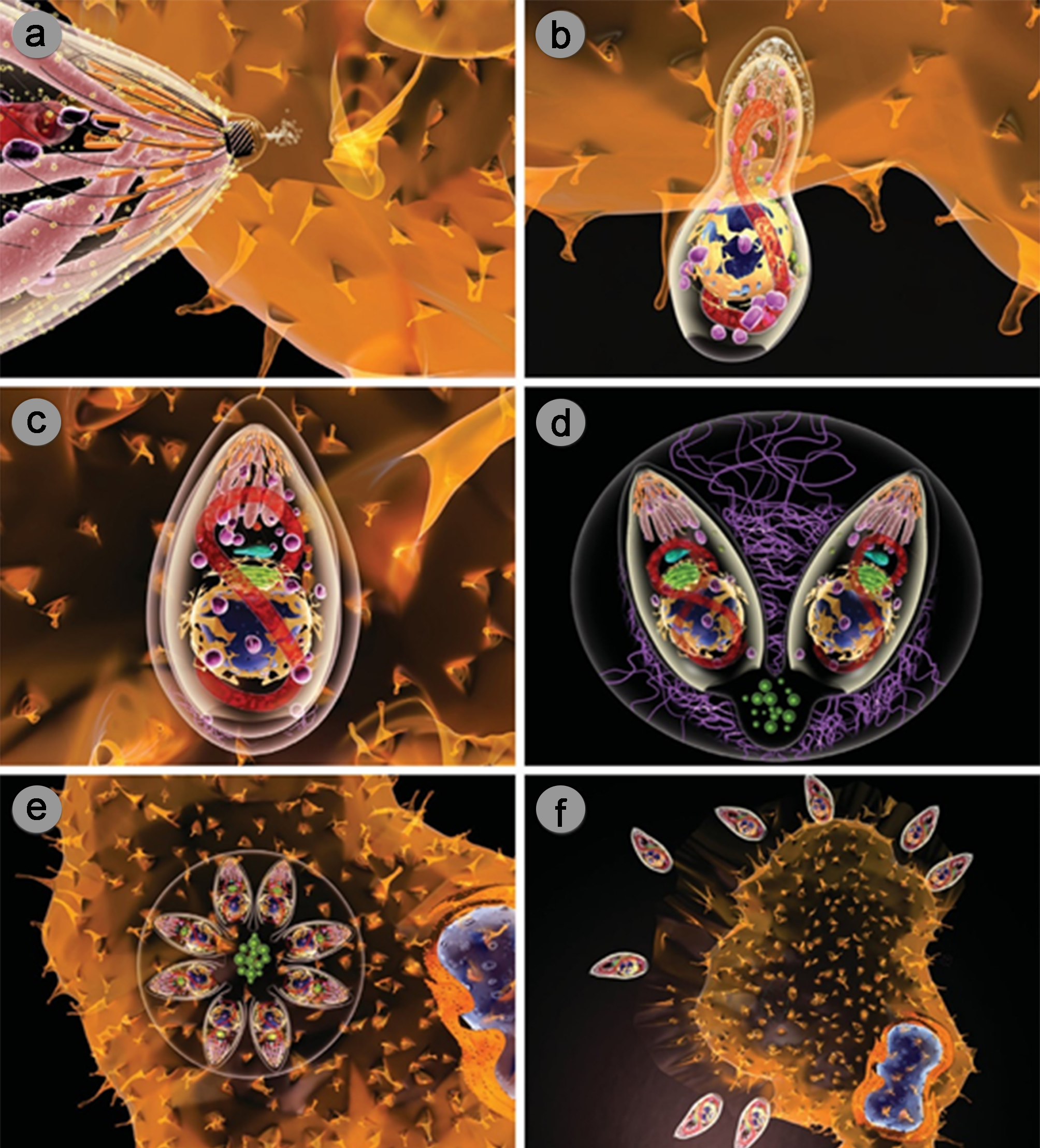

Supplement: Supplementary file 17 — Additional file 17: Figure S17. Sequence of intracellular cycle. a Adhesion, secretion of ropthries. b Moving junction: T. gondii assumes an hourglass shape. c Secretion of dense granules inside the parasitophorous vacuole. d Division, formation of the intravacuolar network, accumulation of acidocalcisomes (green) in the residual body. e Rosette of parasites. f Individualization and egress of parasites. [file 13071_2020_4445_MOESM17_ESM.tif]

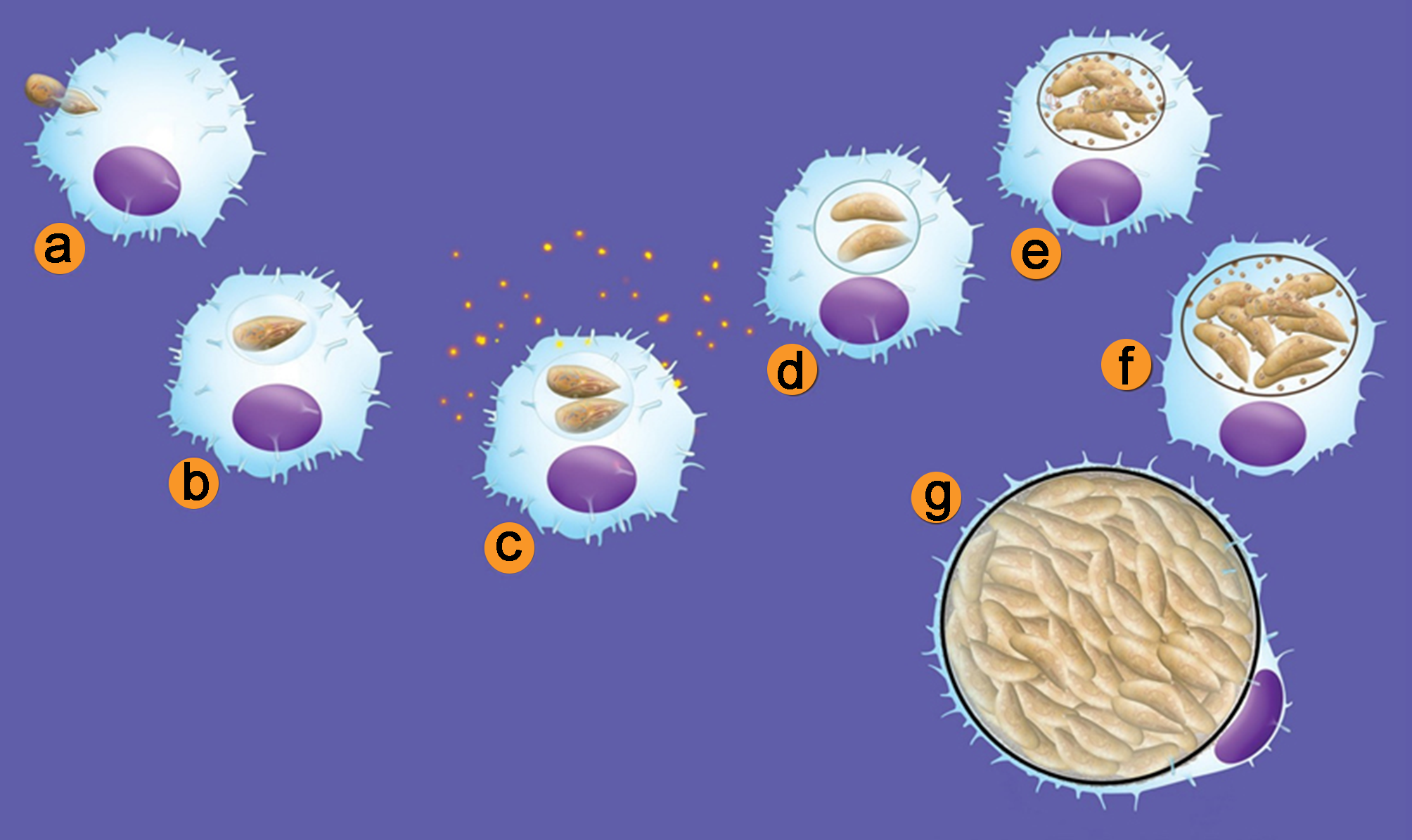

Supplement: Supplementary file 18 — Additional file 18: Figure S18. Cystogenesis. a Invasion. b establishment of parasitophorous vacuole. c Division. d Bradyzoite secretion. e, f Cyst wall thickens, bradyzoites continue to divide. g The cyst inside the host cell. [file 13071_2020_4445_MOESM18_ESM.tif]

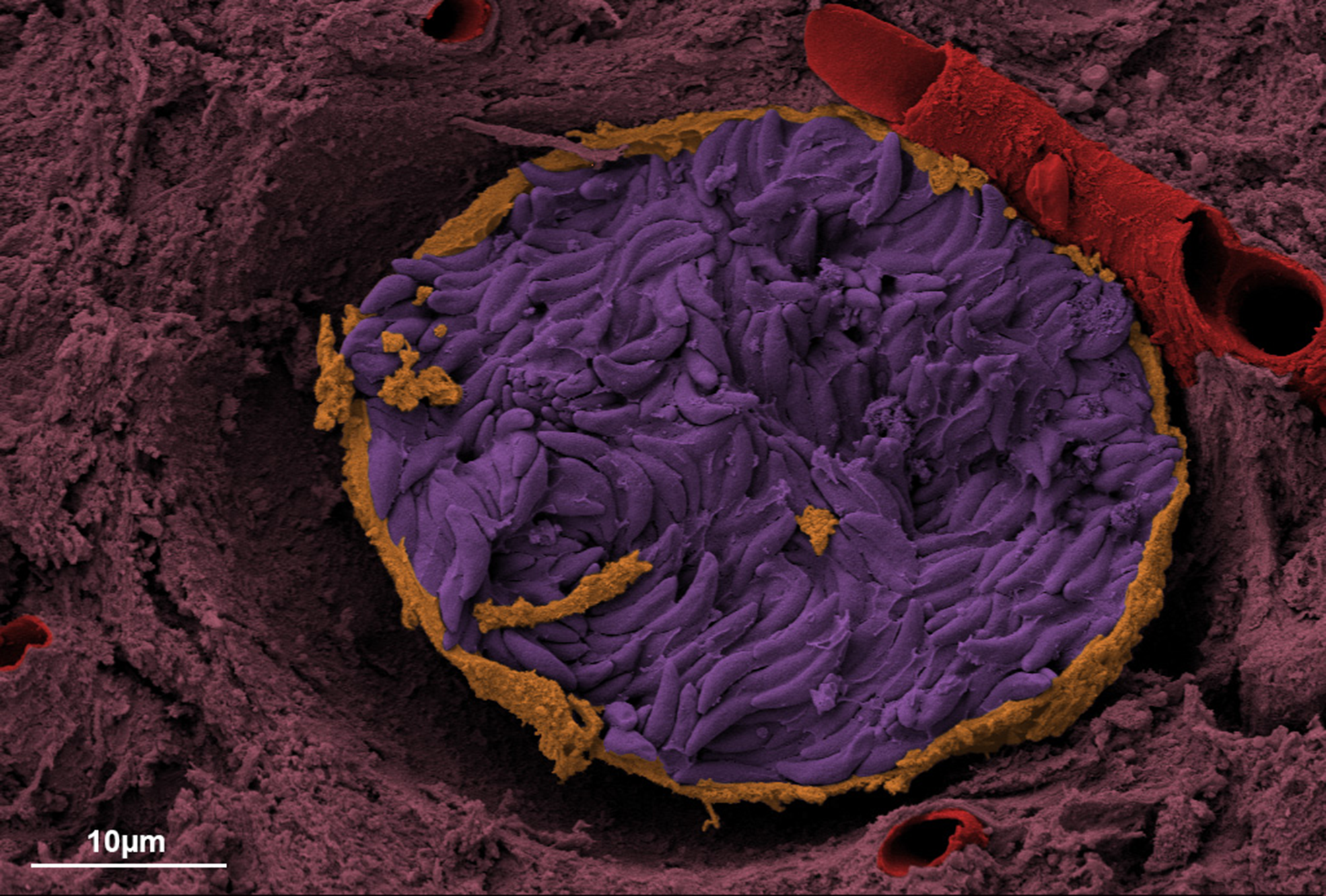

Supplement: Supplementary file 19 — Additional file 19: Figure S19. Tissue cyst in the brain of a mouse. Bradyzoites (purple) surrounded by a thick cyst wall (yellow). Blood vessel (red). [file 13071_2020_4445_MOESM19_ESM.tif]

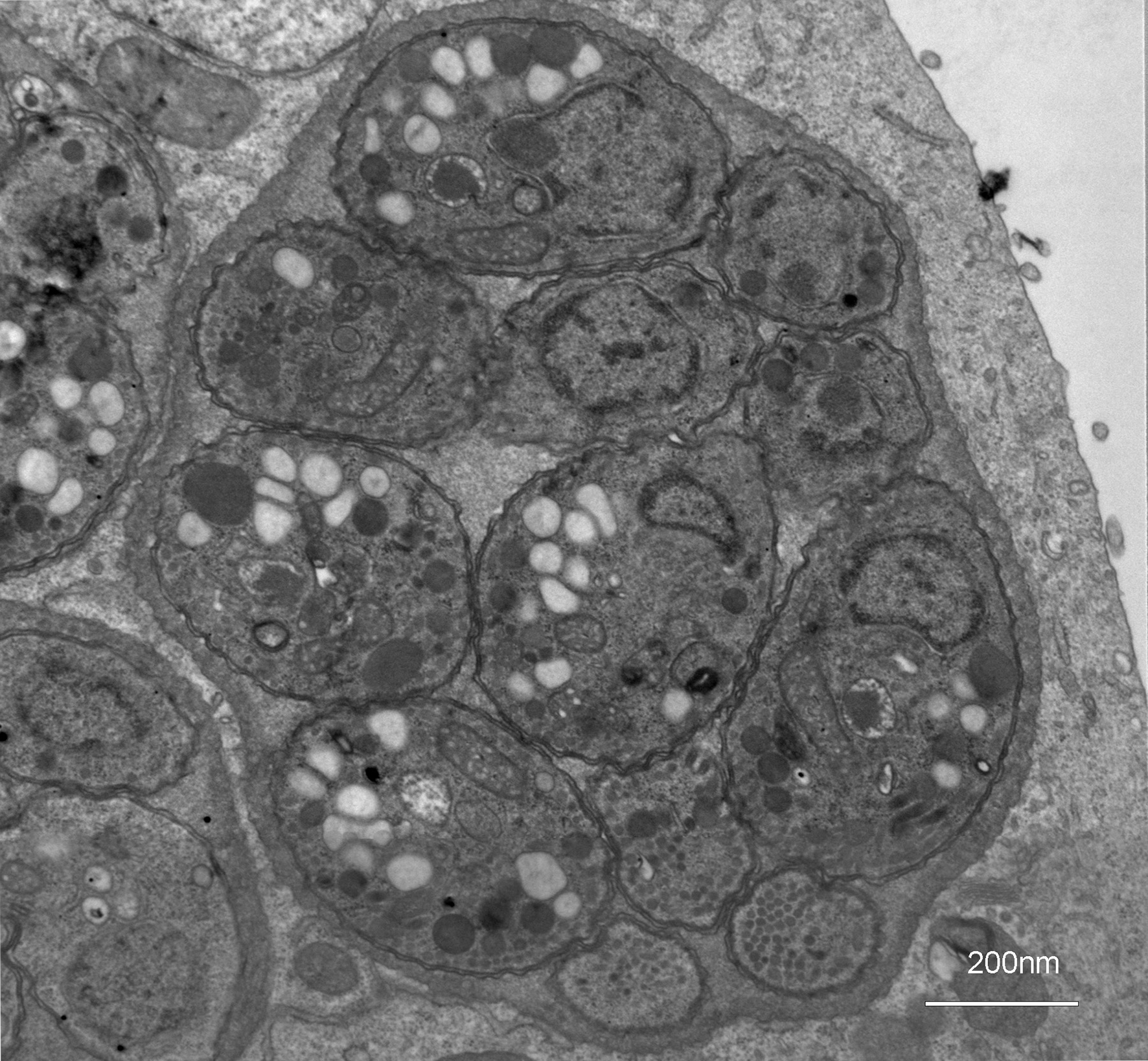

Supplement: Supplementary file 20 — Additional file 20: Figure S20. Tissue cyst. A cystwall (arrowhead). Amylopectin granules (asterisk), granular matrix (black star). [file 13071_2020_4445_MOESM20_ESM.tif]
