# Supplementary material for: The life-cycle of Toxoplasma gondii reviewed using animations
Source: Parasit Vectors. 2020 Nov 23;13:588. doi: 10.1186/s13071-020-04445-z (PMC7686686; doi:10.1186/s13071-020-04445-z)
Supplement: Supplementary file 23 — Additional file 23. Slide show of T. gondii biological cycle, developmental stages and main organelles. [file 13071_2020_4445_MOESM23_ESM.pptx]

## Slide 1
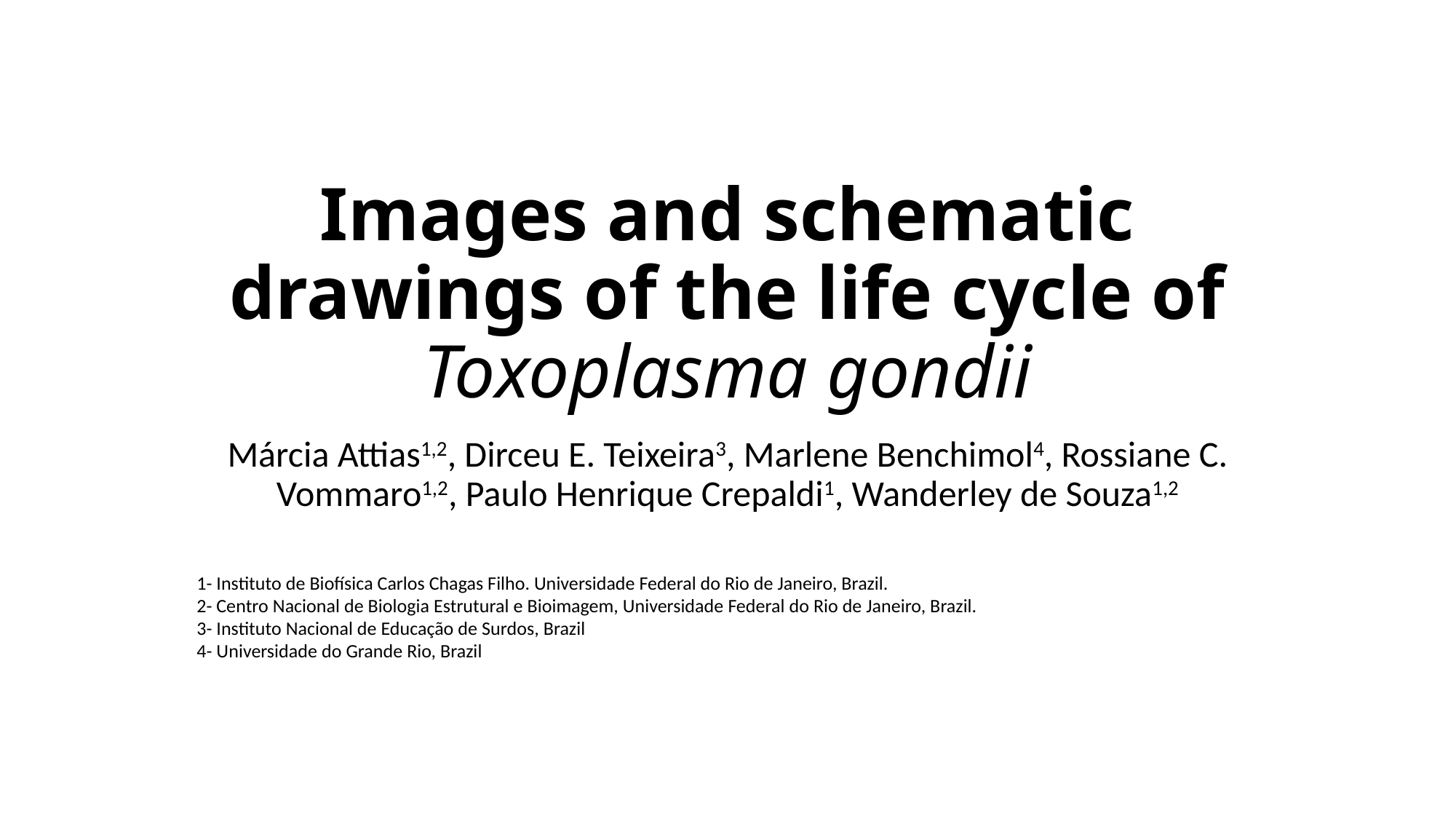

# Images and schematic drawings of the life cycle of Toxoplasma gondii
Márcia Attias1,2, Dirceu E. Teixeira3, Marlene Benchimol4, Rossiane C. Vommaro1,2, Paulo Henrique Crepaldi1, Wanderley de Souza1,2
1- Instituto de Biofísica Carlos Chagas Filho. Universidade Federal do Rio de Janeiro, Brazil.
2- Centro Nacional de Biologia Estrutural e Bioimagem, Universidade Federal do Rio de Janeiro, Brazil.
3- Instituto Nacional de Educação de Surdos, Brazil
4- Universidade do Grande Rio, Brazil

## Slide 2
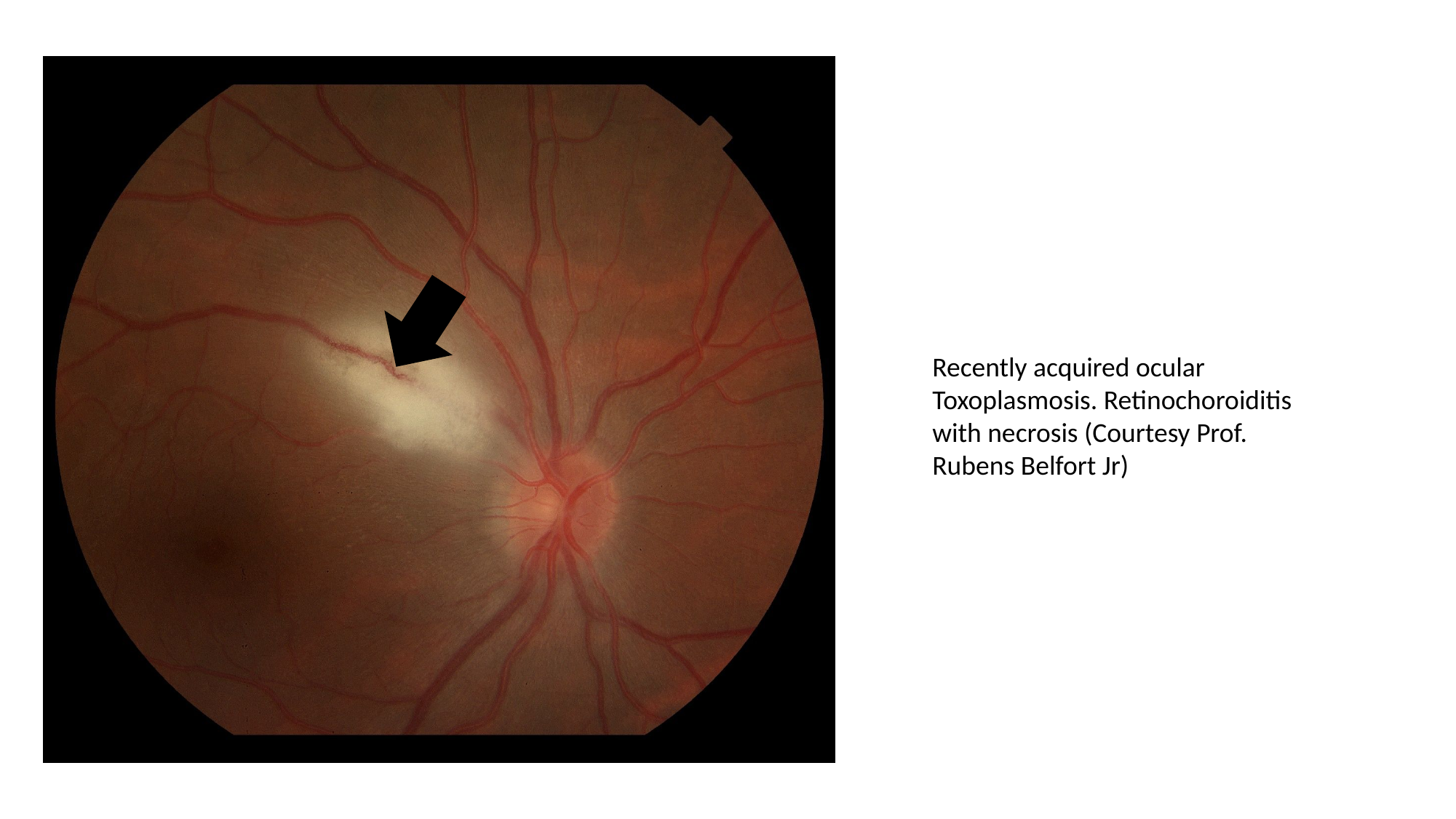

Recently acquired ocular Toxoplasmosis. Retinochoroiditis with necrosis (Courtesy Prof. Rubens Belfort Jr)

## Slide 3
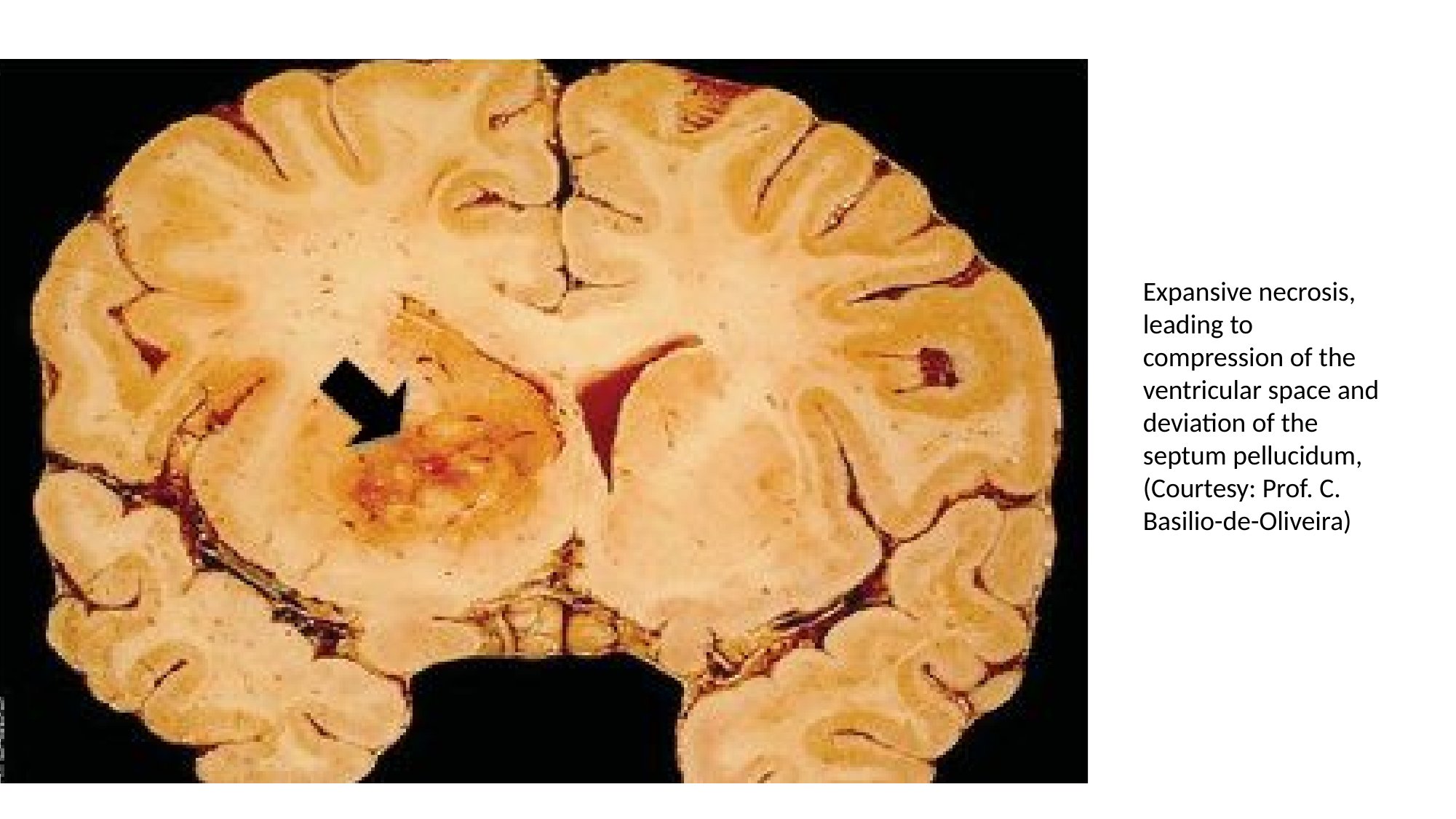

Expansive necrosis, leading to compression of the ventricular space and deviation of the septum pellucidum, (Courtesy: Prof. C. Basilio-de-Oliveira)

## Slide 4
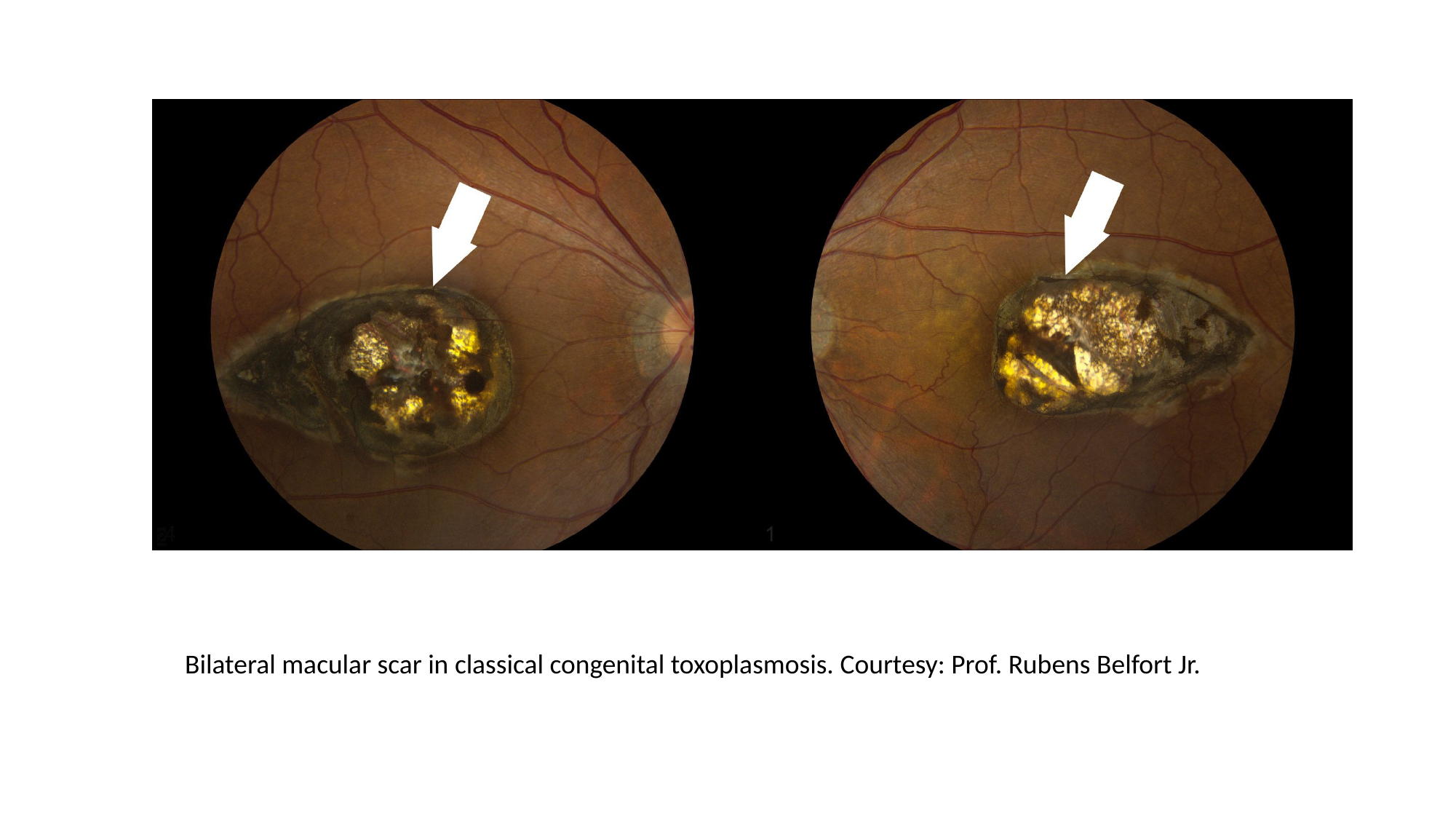

Bilateral macular scar in classical congenital toxoplasmosis. Courtesy: Prof. Rubens Belfort Jr.

## Slide 5
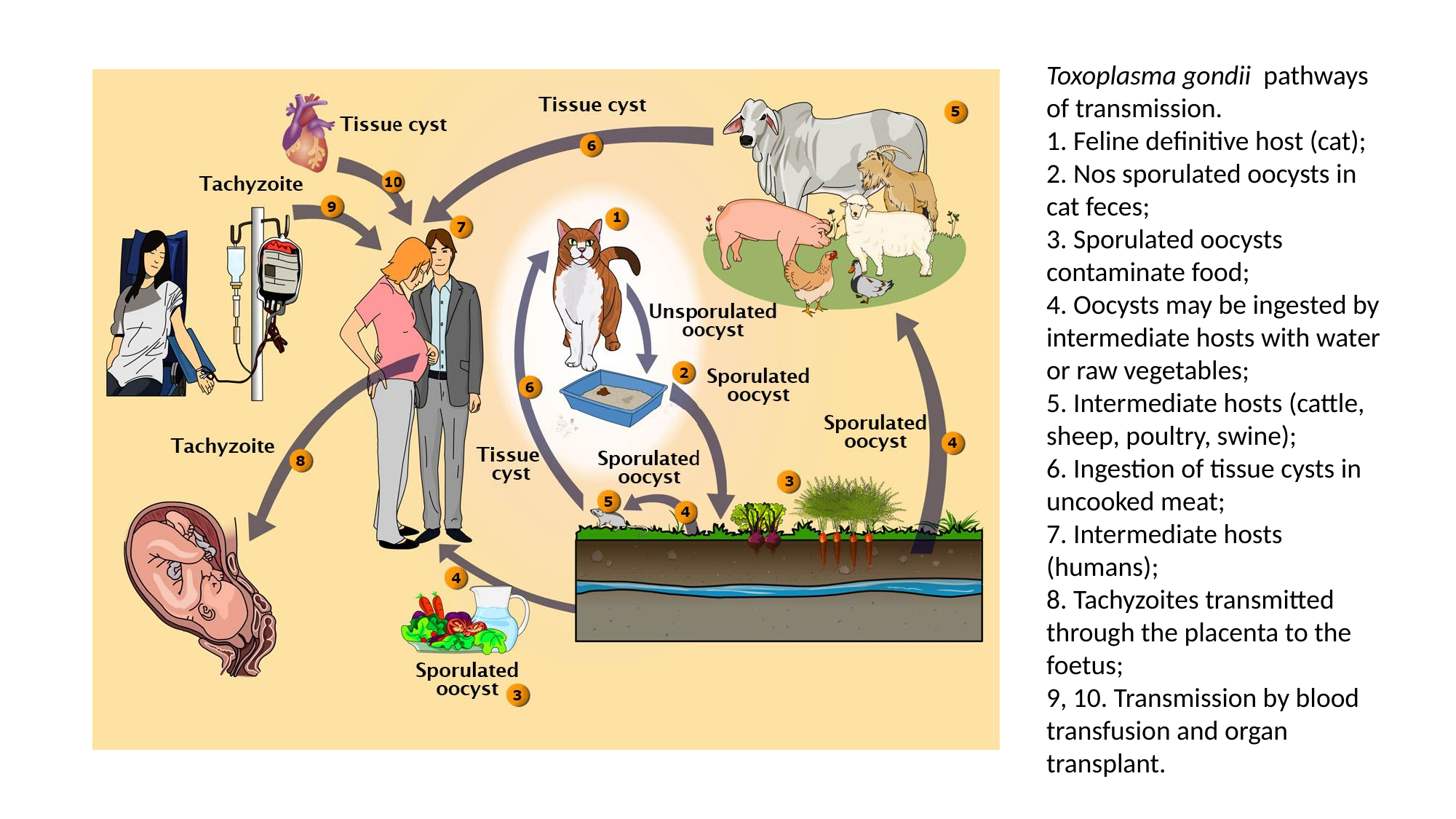

Toxoplasma gondii pathways of transmission.
1. Feline definitive host (cat);
2. Nos sporulated oocysts in cat feces;
3. Sporulated oocysts contaminate food;
4. Oocysts may be ingested by intermediate hosts with water or raw vegetables;
5. Intermediate hosts (cattle, sheep, poultry, swine);
6. Ingestion of tissue cysts in uncooked meat;
7. Intermediate hosts (humans);
8. Tachyzoites transmitted through the placenta to the foetus;
9, 10. Transmission by blood transfusion and organ transplant.

## Slide 6
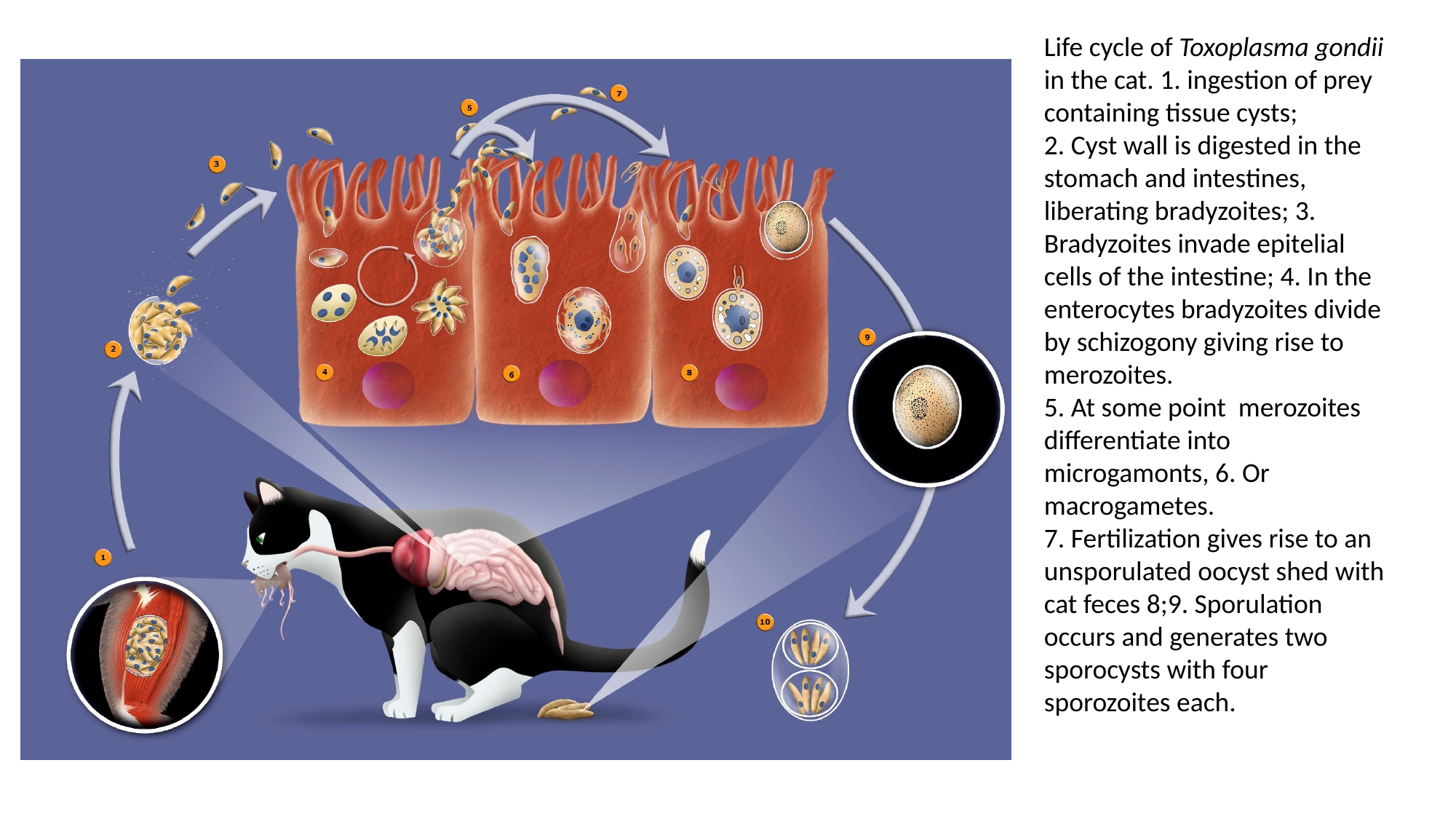

Life cycle of Toxoplasma gondii in the cat. 1. ingestion of prey containing tissue cysts;
2. Cyst wall is digested in the stomach and intestines, liberating bradyzoites; 3. Bradyzoites invade epitelial cells of the intestine; 4. In the enterocytes bradyzoites divide by schizogony giving rise to merozoites.
5. At some point merozoites differentiate into microgamonts, 6. Or macrogametes.
7. Fertilization gives rise to an unsporulated oocyst shed with cat feces 8;9. Sporulation occurs and generates two sporocysts with four sporozoites each.

## Slide 7
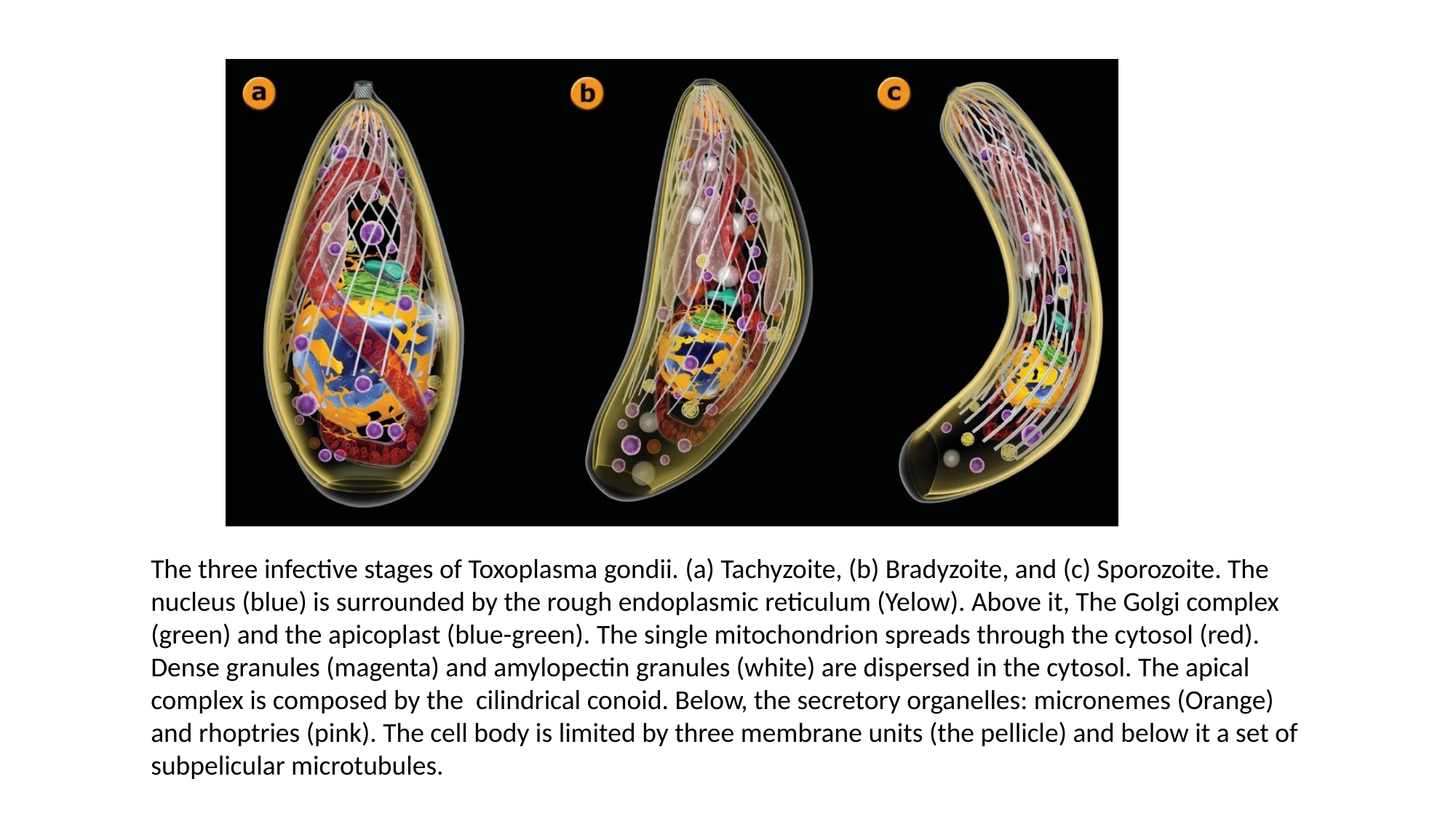

The three infective stages of Toxoplasma gondii. (a) Tachyzoite, (b) Bradyzoite, and (c) Sporozoite. The nucleus (blue) is surrounded by the rough endoplasmic reticulum (Yelow). Above it, The Golgi complex (green) and the apicoplast (blue-green). The single mitochondrion spreads through the cytosol (red). Dense granules (magenta) and amylopectin granules (white) are dispersed in the cytosol. The apical complex is composed by the cilindrical conoid. Below, the secretory organelles: micronemes (Orange) and rhoptries (pink). The cell body is limited by three membrane units (the pellicle) and below it a set of subpelicular microtubules.

## Slide 8
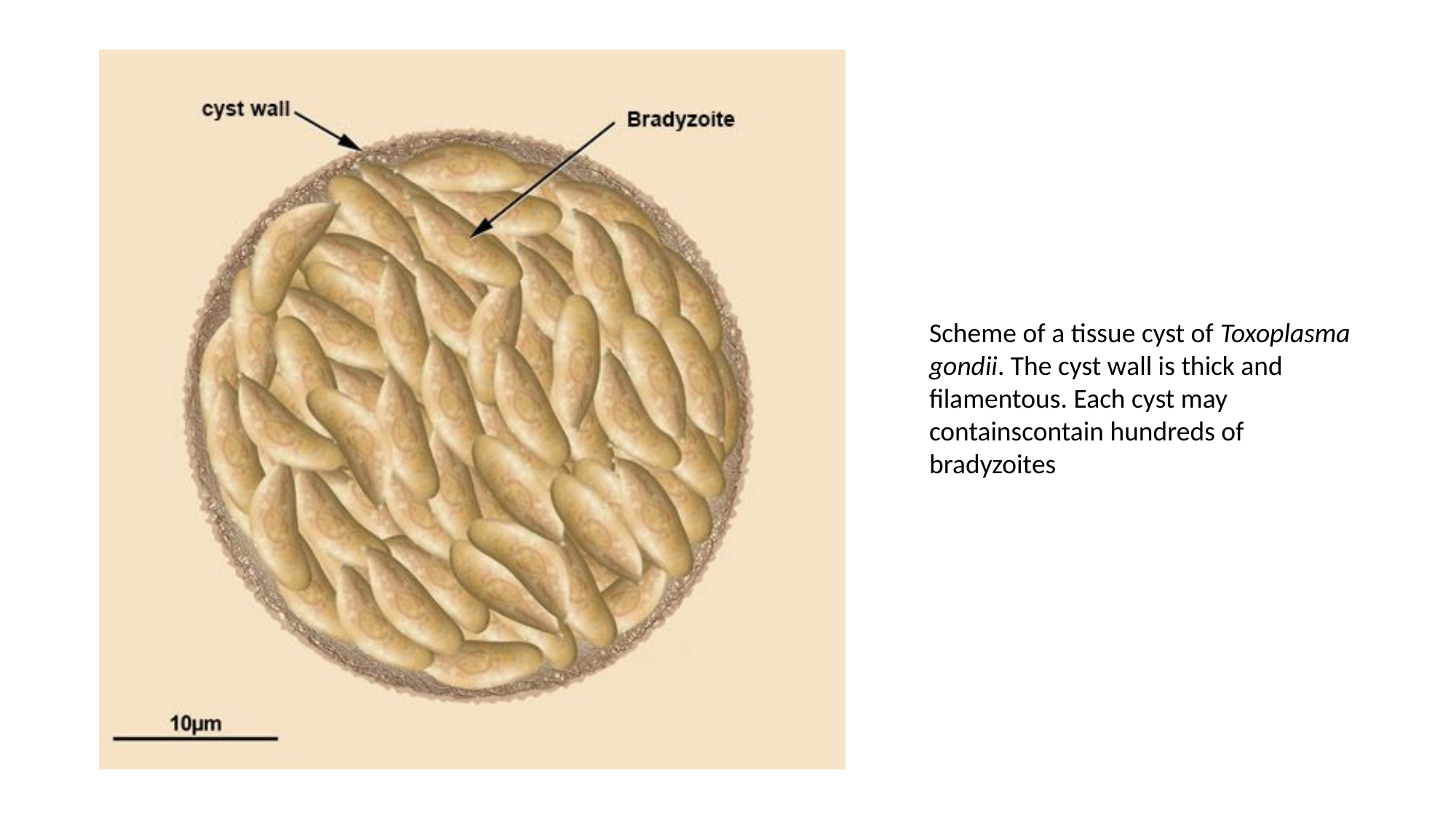

Scheme of a tissue cyst of Toxoplasma gondii. The cyst wall is thick and filamentous. Each cyst may containscontain hundreds of bradyzoites

## Slide 9
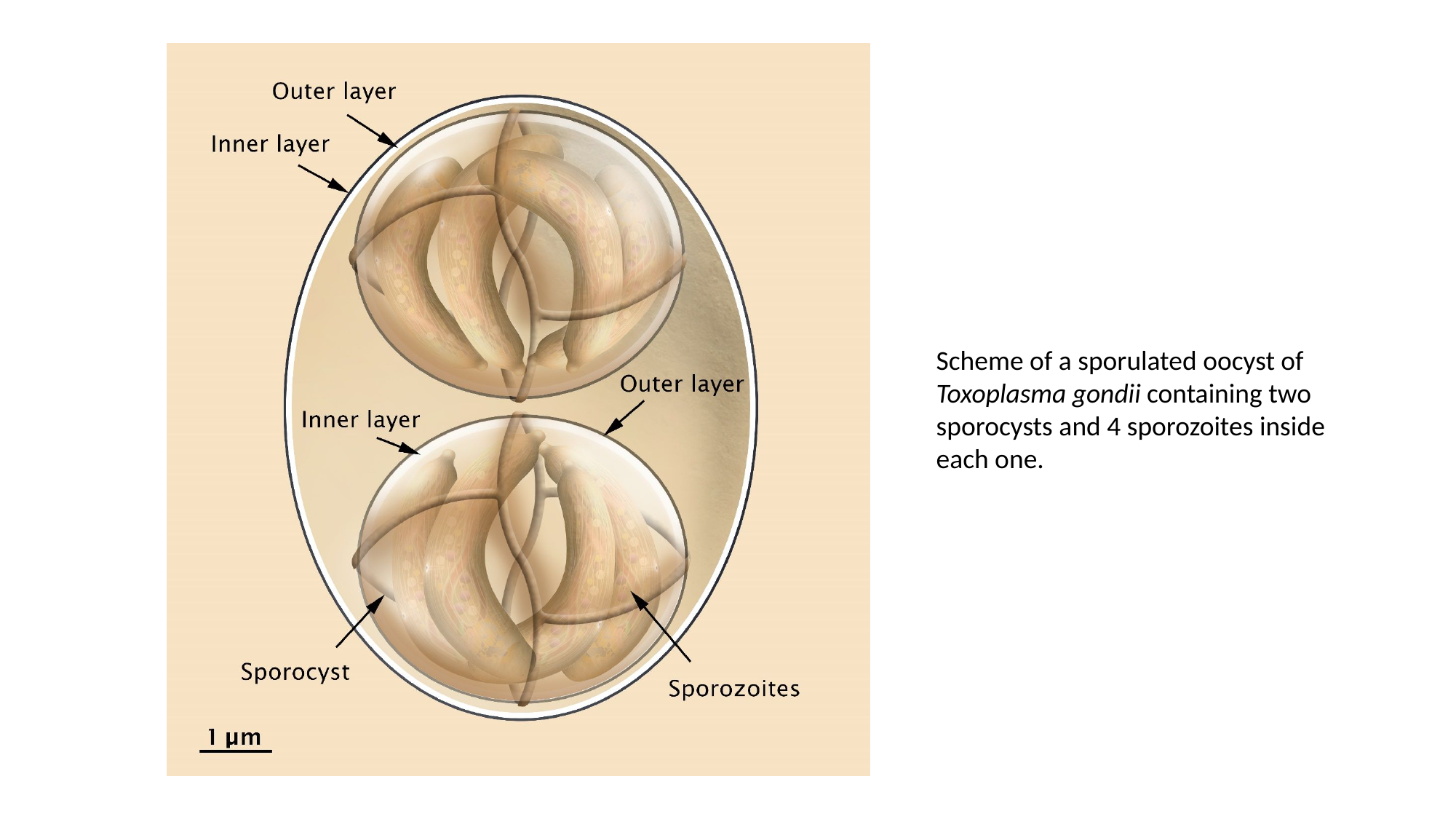

Scheme of a sporulated oocyst of Toxoplasma gondii containing two sporocysts and 4 sporozoites inside each one.

## Slide 10
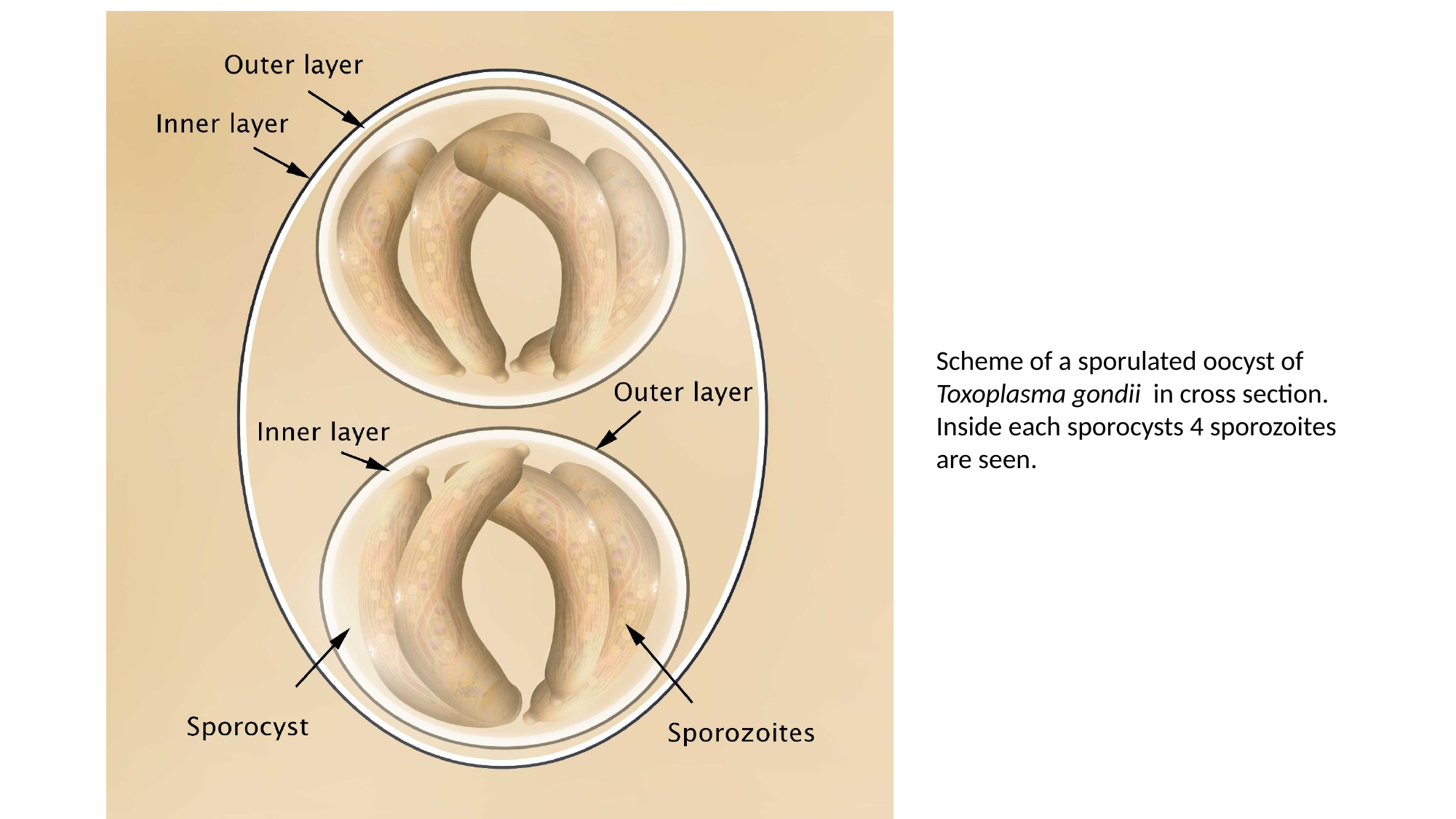

Scheme of a sporulated oocyst of Toxoplasma gondii in cross section. Inside each sporocysts 4 sporozoites are seen.

## Slide 11
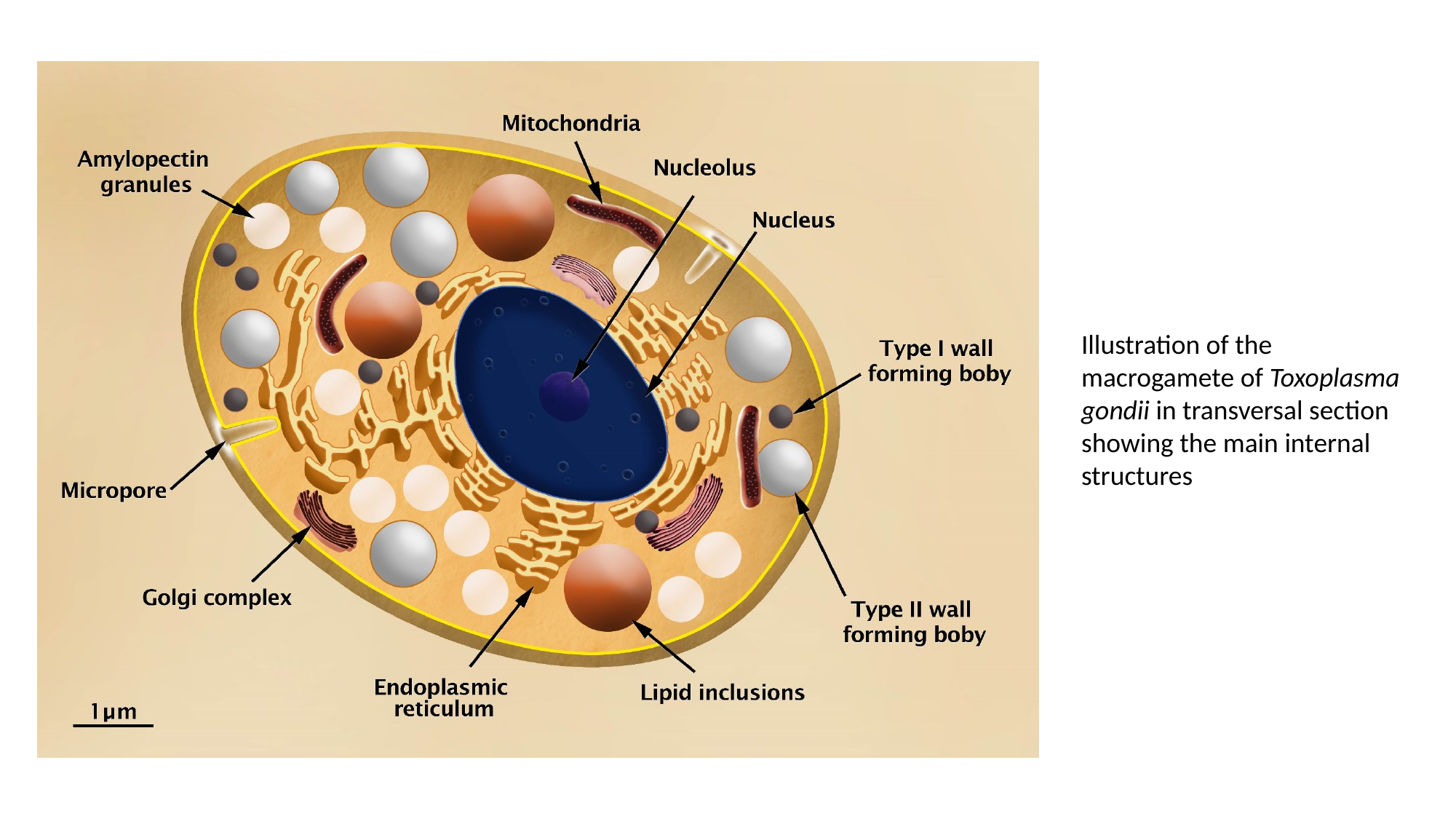

Illustration of the macrogamete of Toxoplasma gondii in transversal section showing the main internal structures

## Slide 12
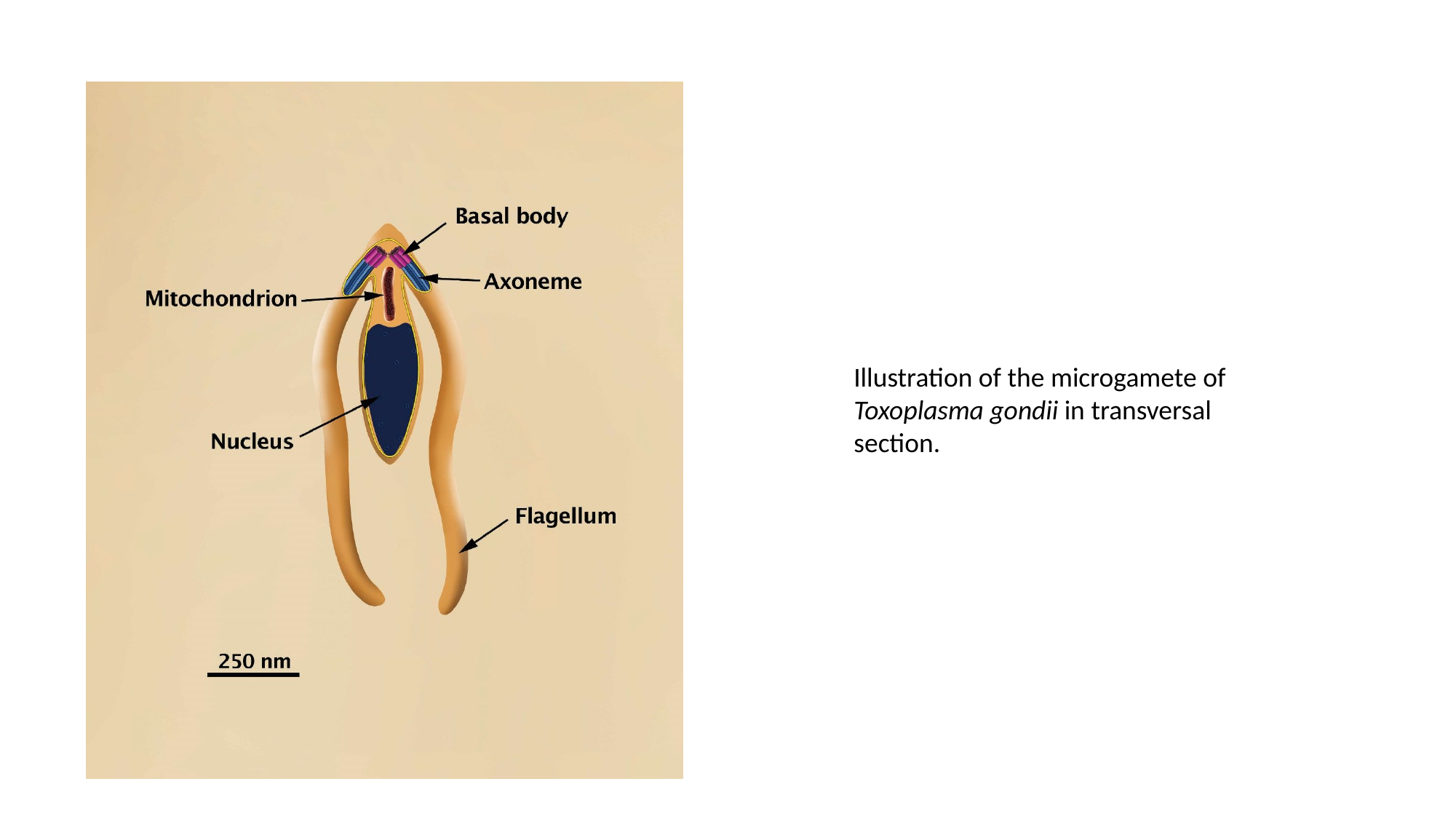

Illustration of the microgamete of Toxoplasma gondii in transversal section.

## Slide 13
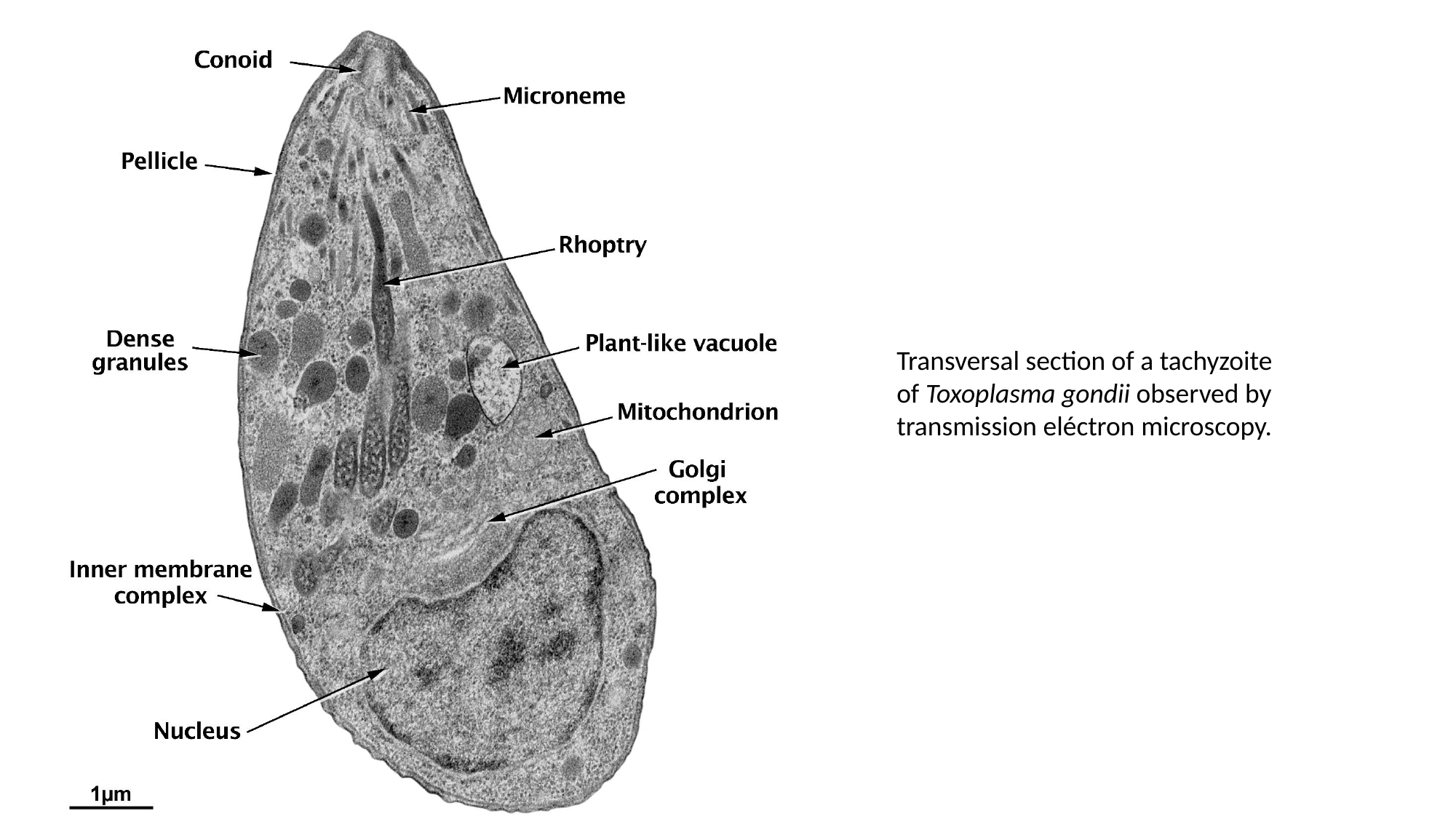

Transversal section of a tachyzoite of Toxoplasma gondii observed by transmission eléctron microscopy.

## Slide 14
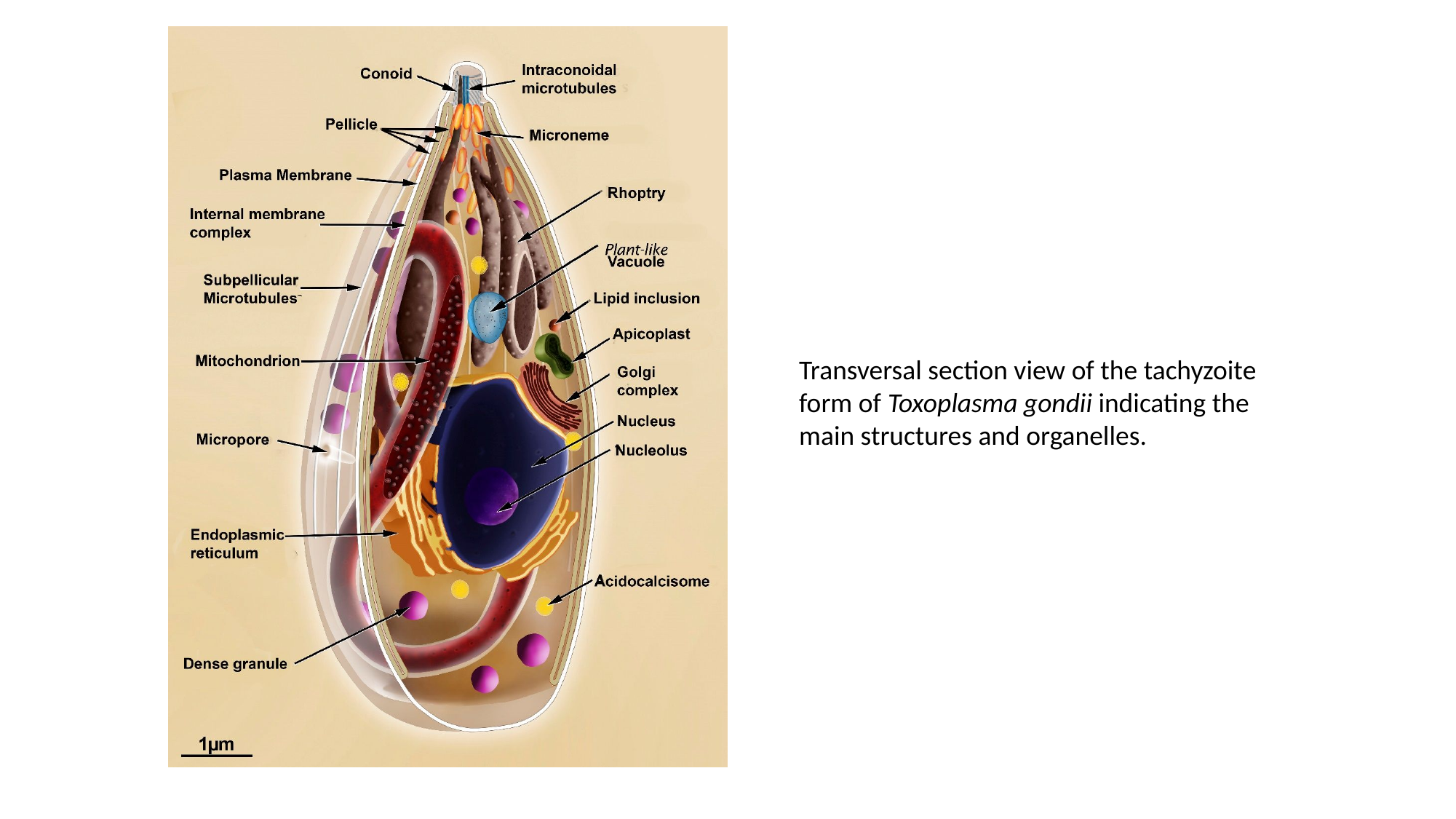

Transversal section view of the tachyzoite form of Toxoplasma gondii indicating the main structures and organelles.

## Slide 15
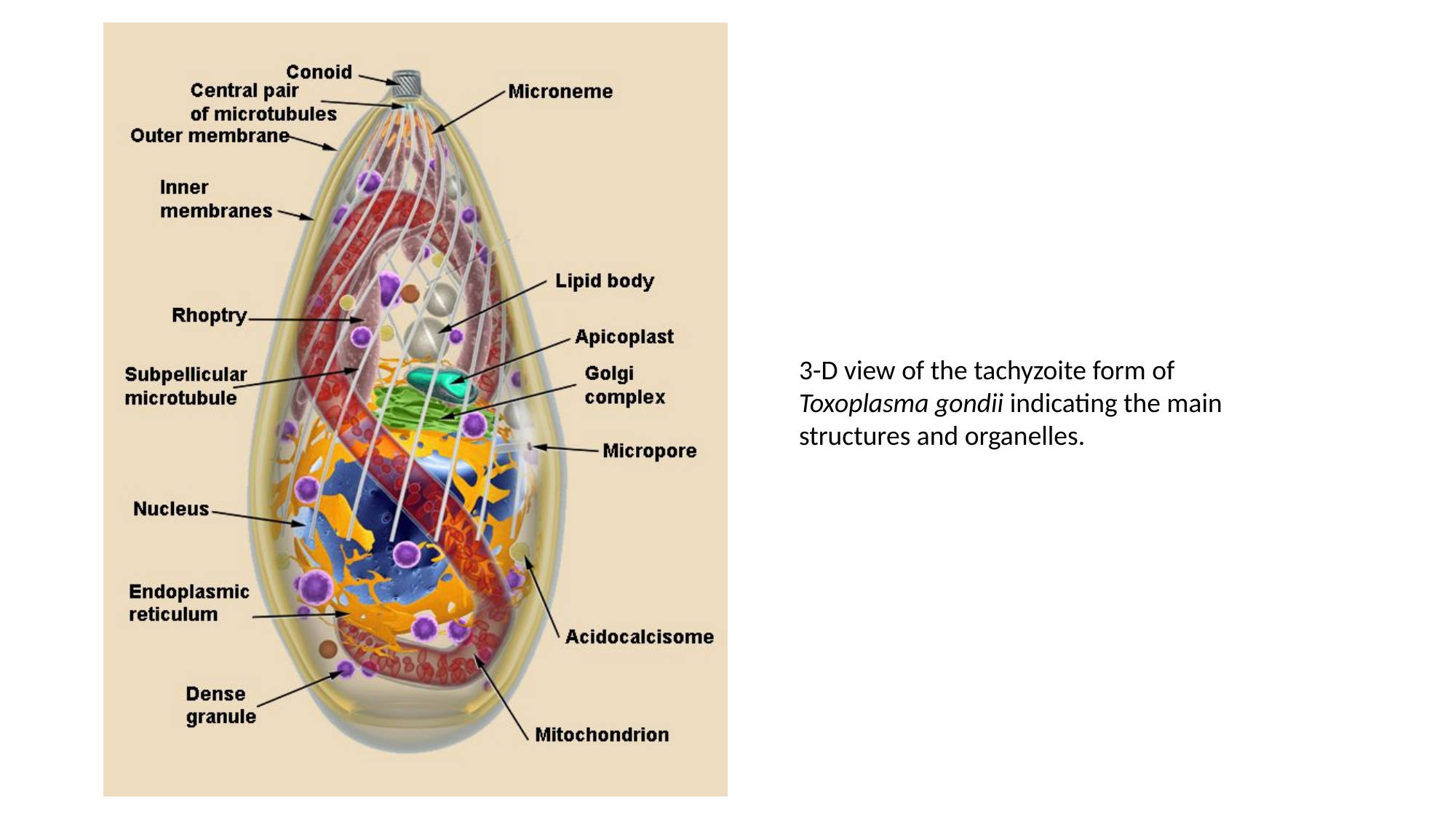

3-D view of the tachyzoite form of Toxoplasma gondii indicating the main structures and organelles.

## Slide 16
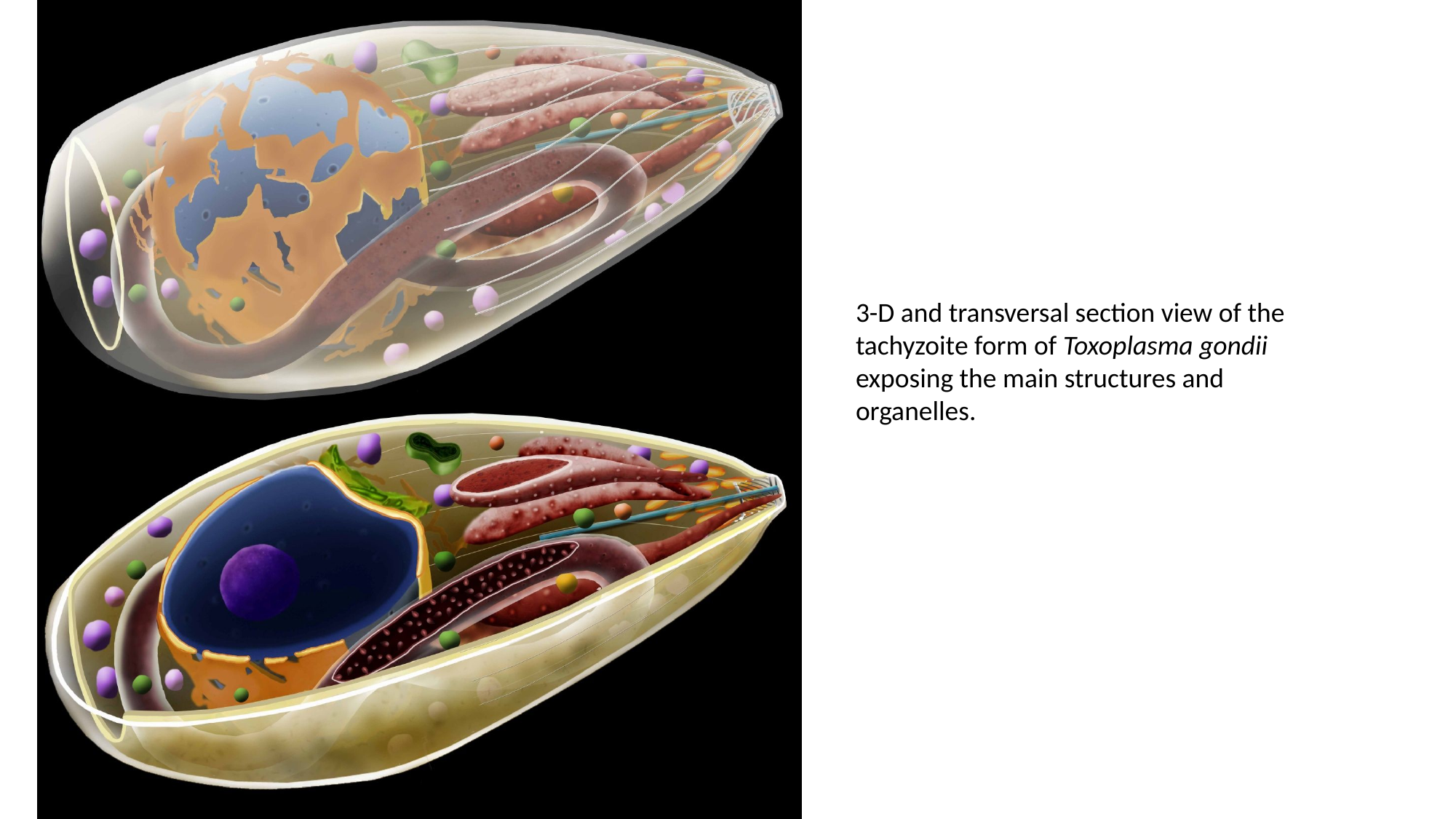

3-D and transversal section view of the tachyzoite form of Toxoplasma gondii exposing the main structures and organelles.

## Slide 17
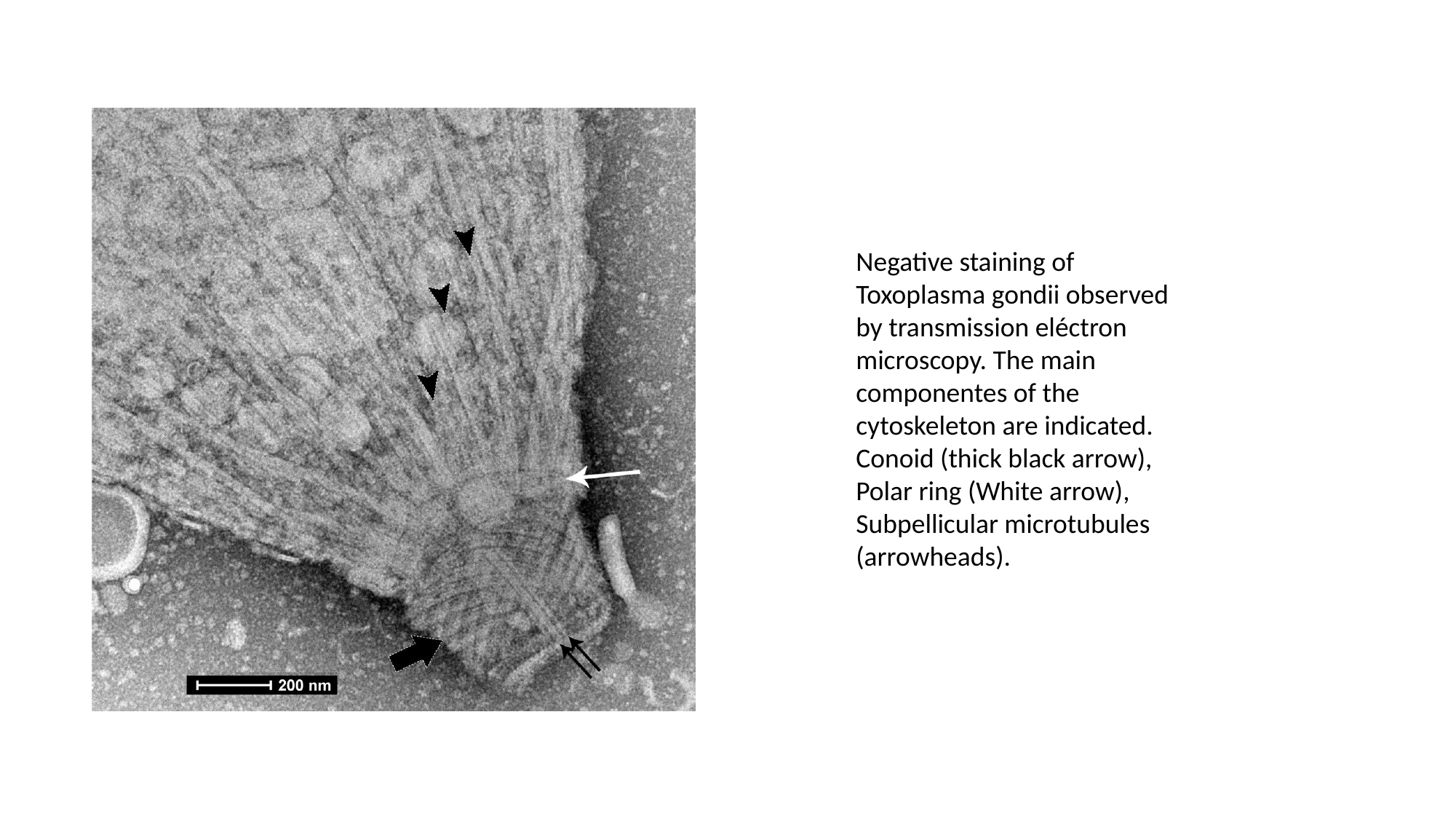

Negative staining of Toxoplasma gondii observed by transmission eléctron microscopy. The main componentes of the cytoskeleton are indicated. Conoid (thick black arrow),
Polar ring (White arrow), Subpellicular microtubules (arrowheads).

## Slide 18
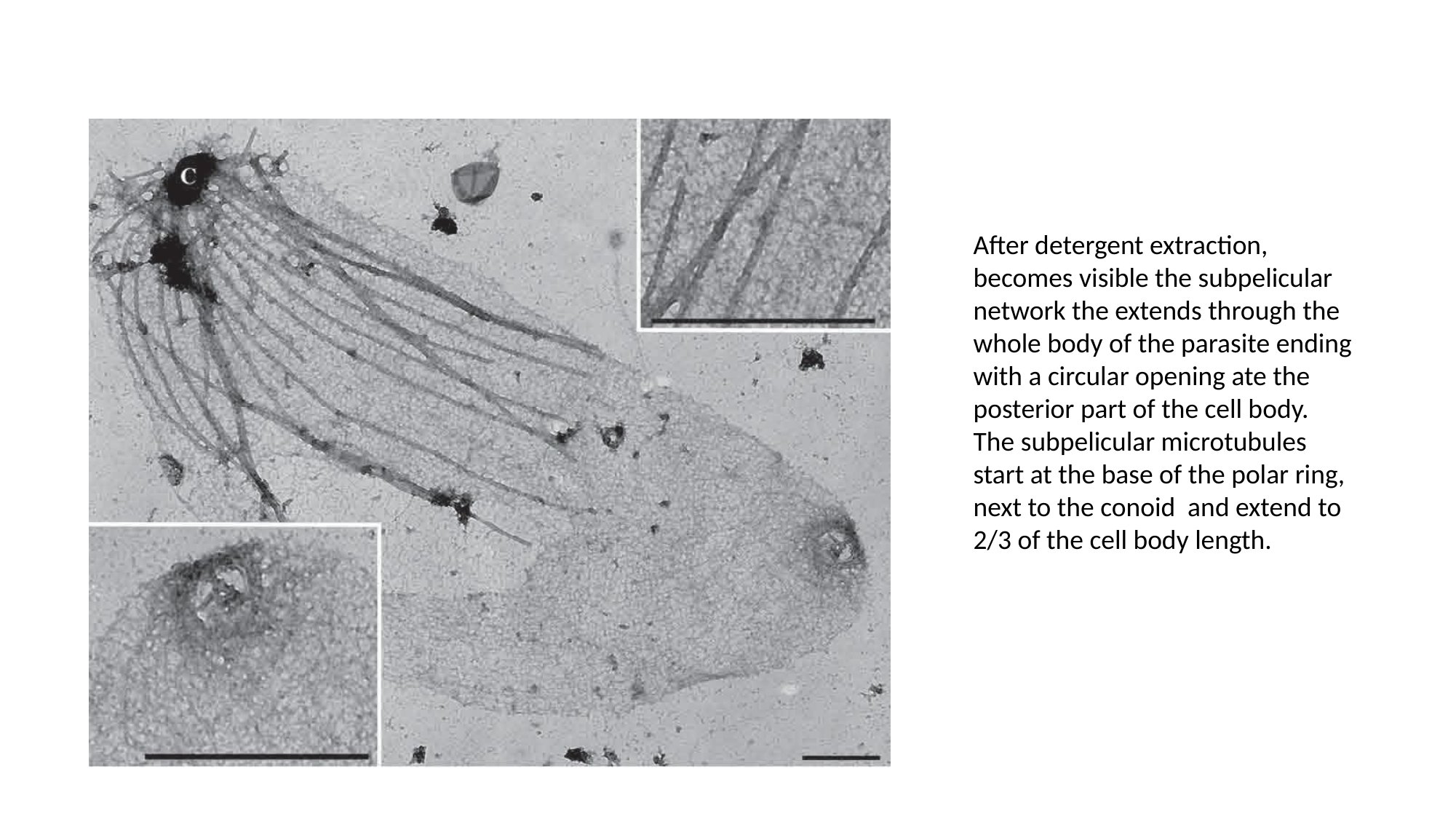

After detergent extraction, becomes visible the subpelicular network the extends through the whole body of the parasite ending with a circular opening ate the posterior part of the cell body. The subpelicular microtubules start at the base of the polar ring, next to the conoid and extend to 2/3 of the cell body length.

## Slide 19
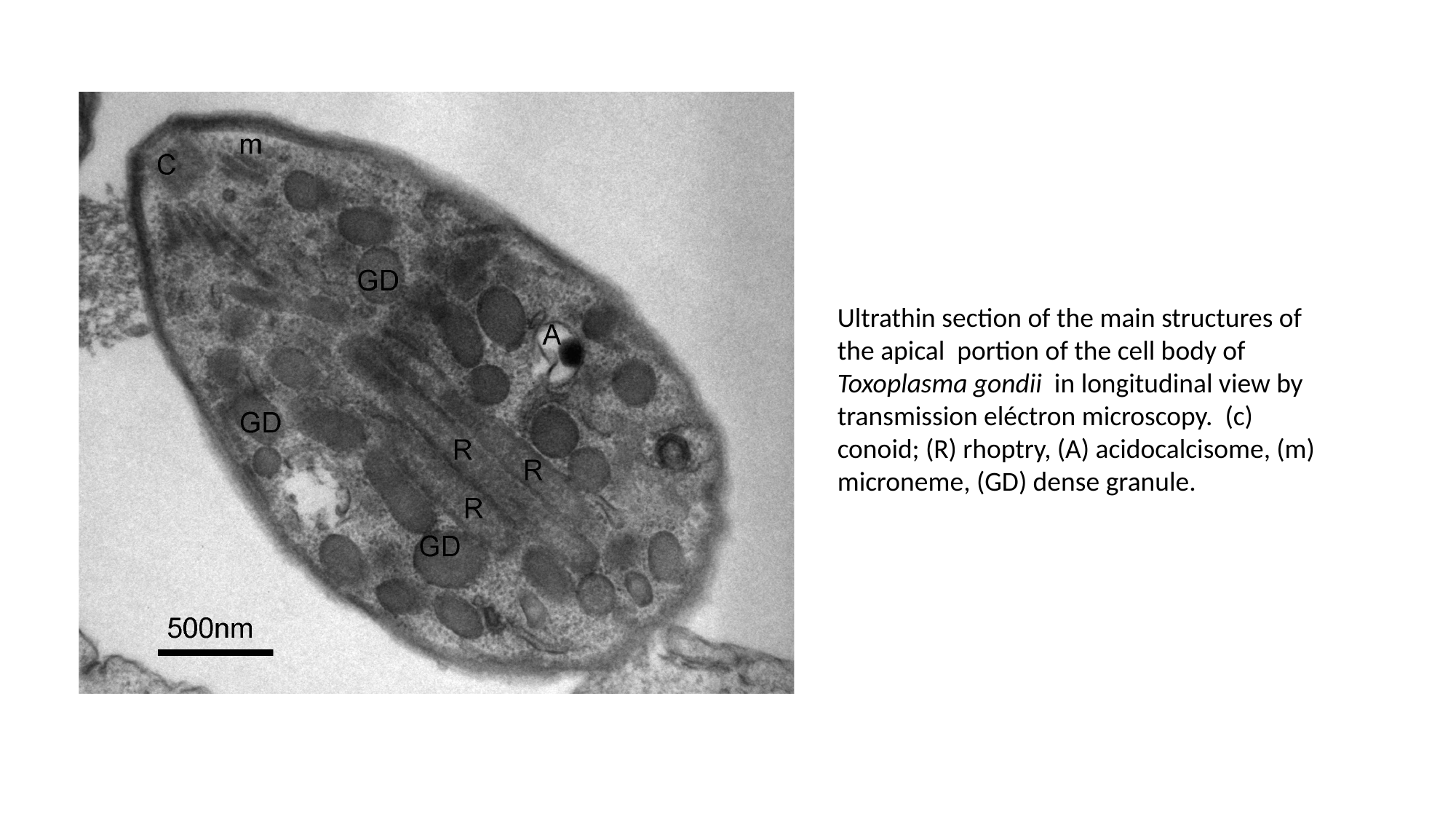

Ultrathin section of the main structures of the apical portion of the cell body of Toxoplasma gondii in longitudinal view by transmission eléctron microscopy. (c) conoid; (R) rhoptry, (A) acidocalcisome, (m) microneme, (GD) dense granule.

## Slide 20
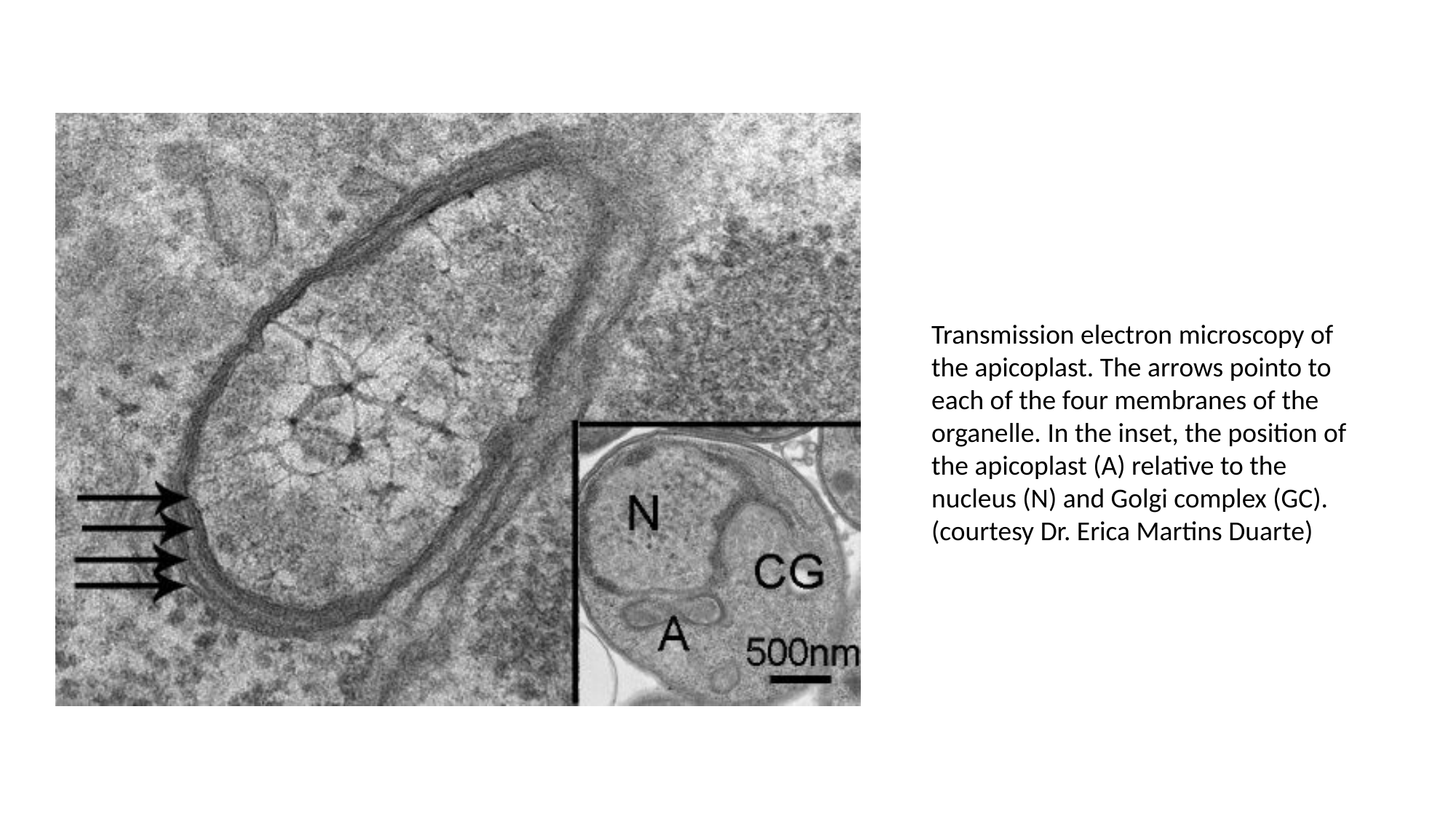

Transmission electron microscopy of the apicoplast. The arrows pointo to each of the four membranes of the organelle. In the inset, the position of the apicoplast (A) relative to the nucleus (N) and Golgi complex (GC). (courtesy Dr. Erica Martins Duarte)

## Slide 21
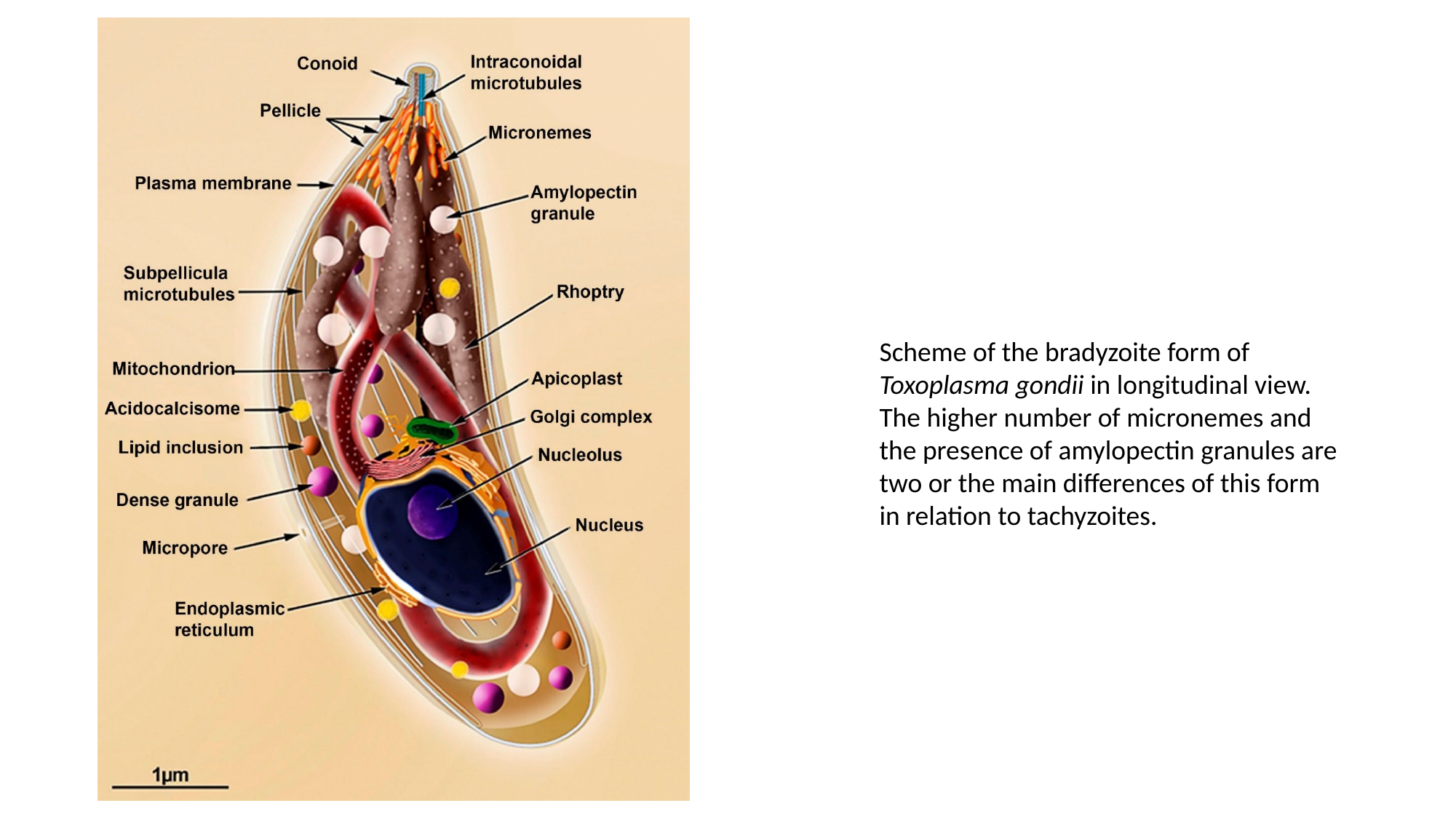

Scheme of the bradyzoite form of Toxoplasma gondii in longitudinal view. The higher number of micronemes and the presence of amylopectin granules are two or the main differences of this form in relation to tachyzoites.

## Slide 22
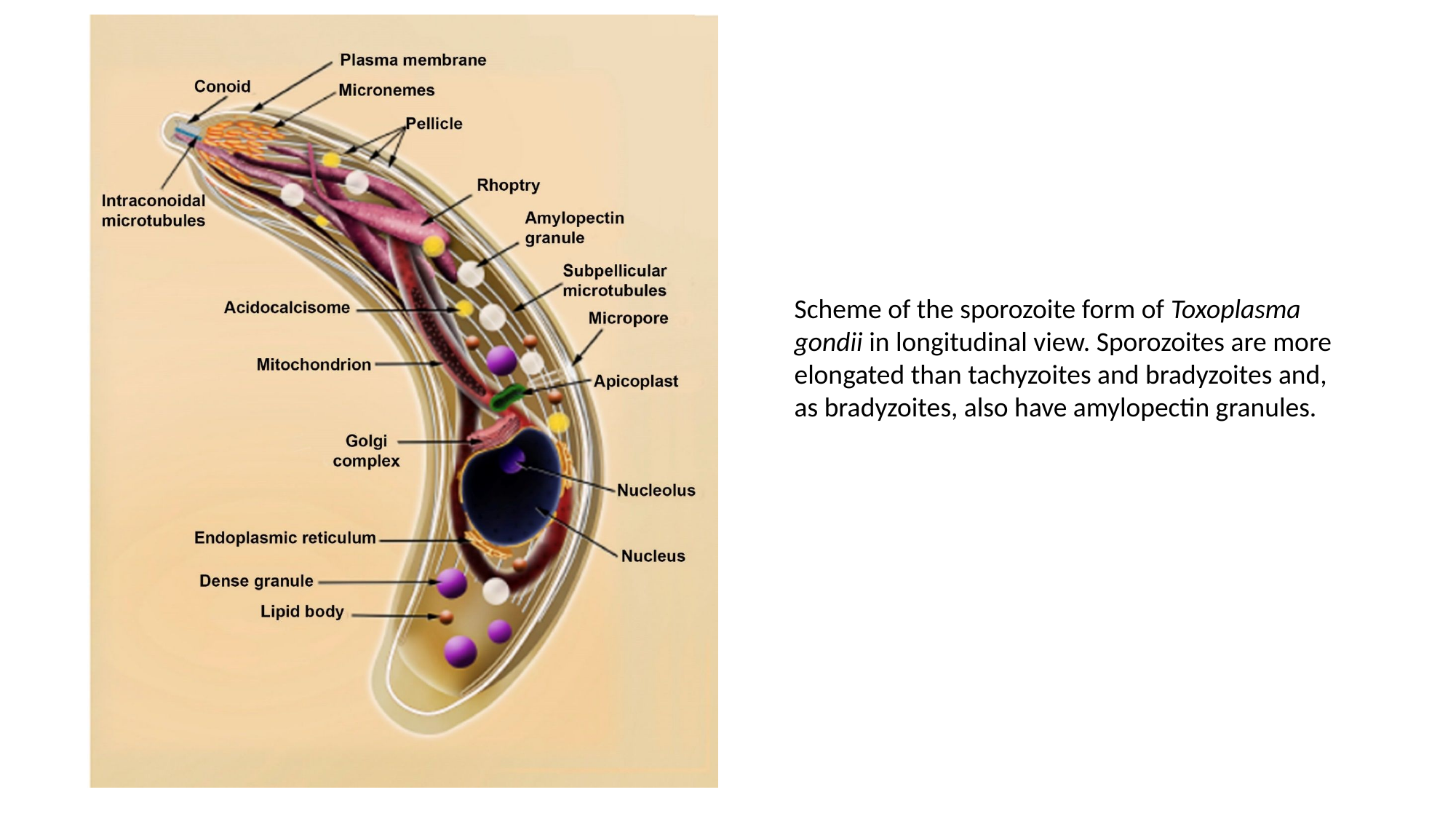

Scheme of the sporozoite form of Toxoplasma gondii in longitudinal view. Sporozoites are more elongated than tachyzoites and bradyzoites and, as bradyzoites, also have amylopectin granules.

## Slide 23
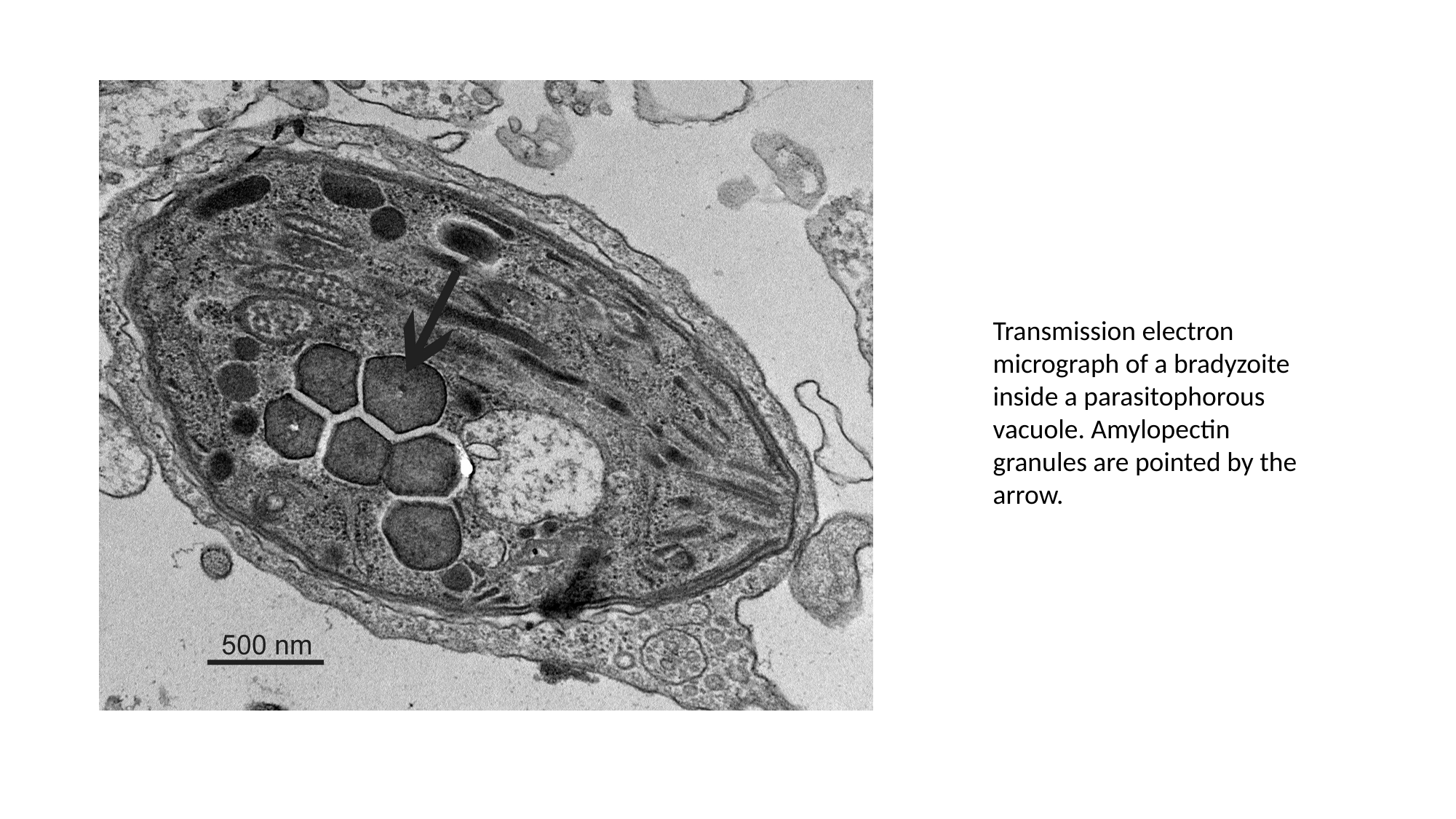

Transmission electron micrograph of a bradyzoite inside a parasitophorous vacuole. Amylopectin granules are pointed by the arrow.

## Slide 24
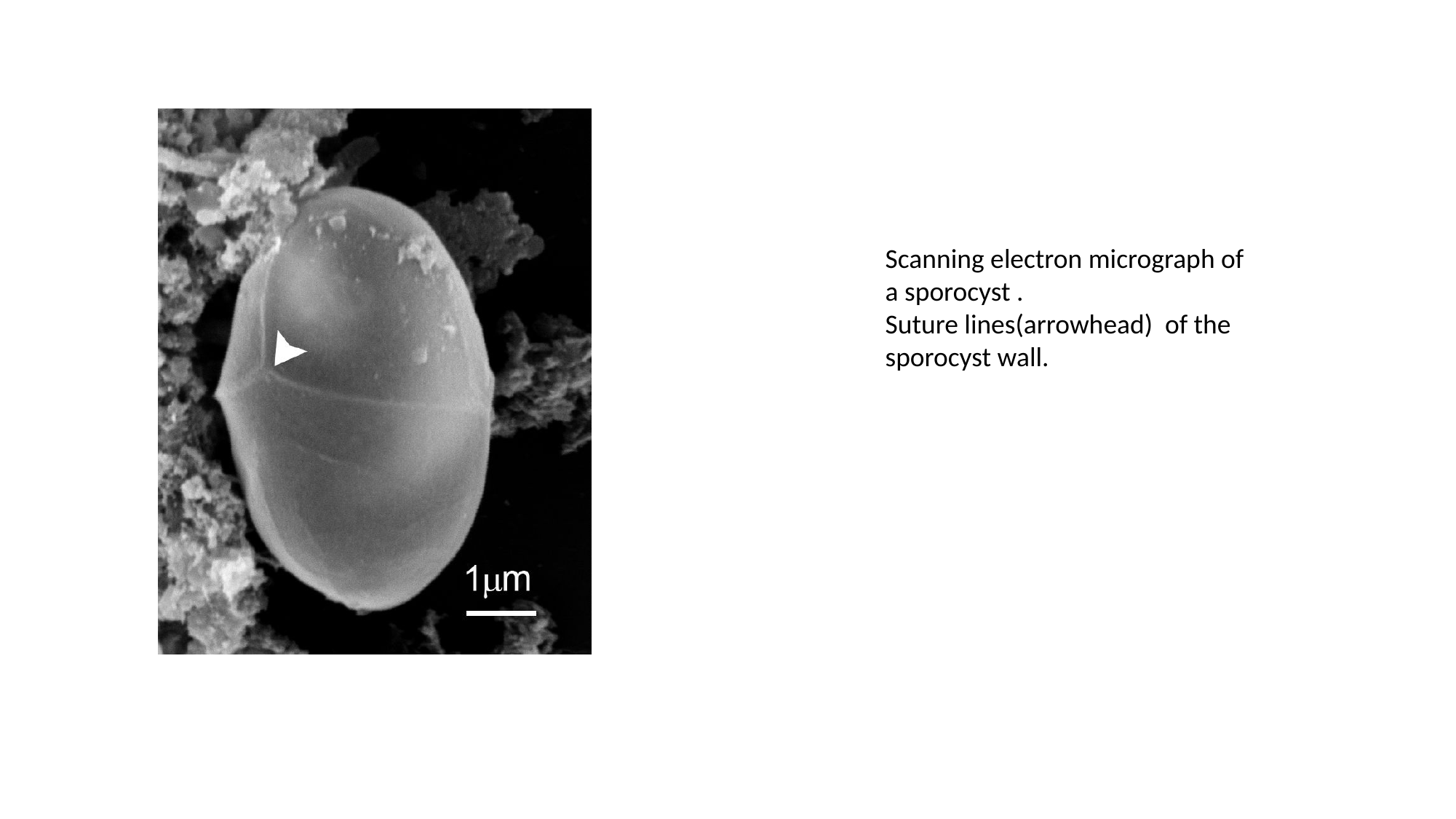

Scanning electron micrograph of a sporocyst .
Suture lines(arrowhead) of the sporocyst wall.

## Slide 25
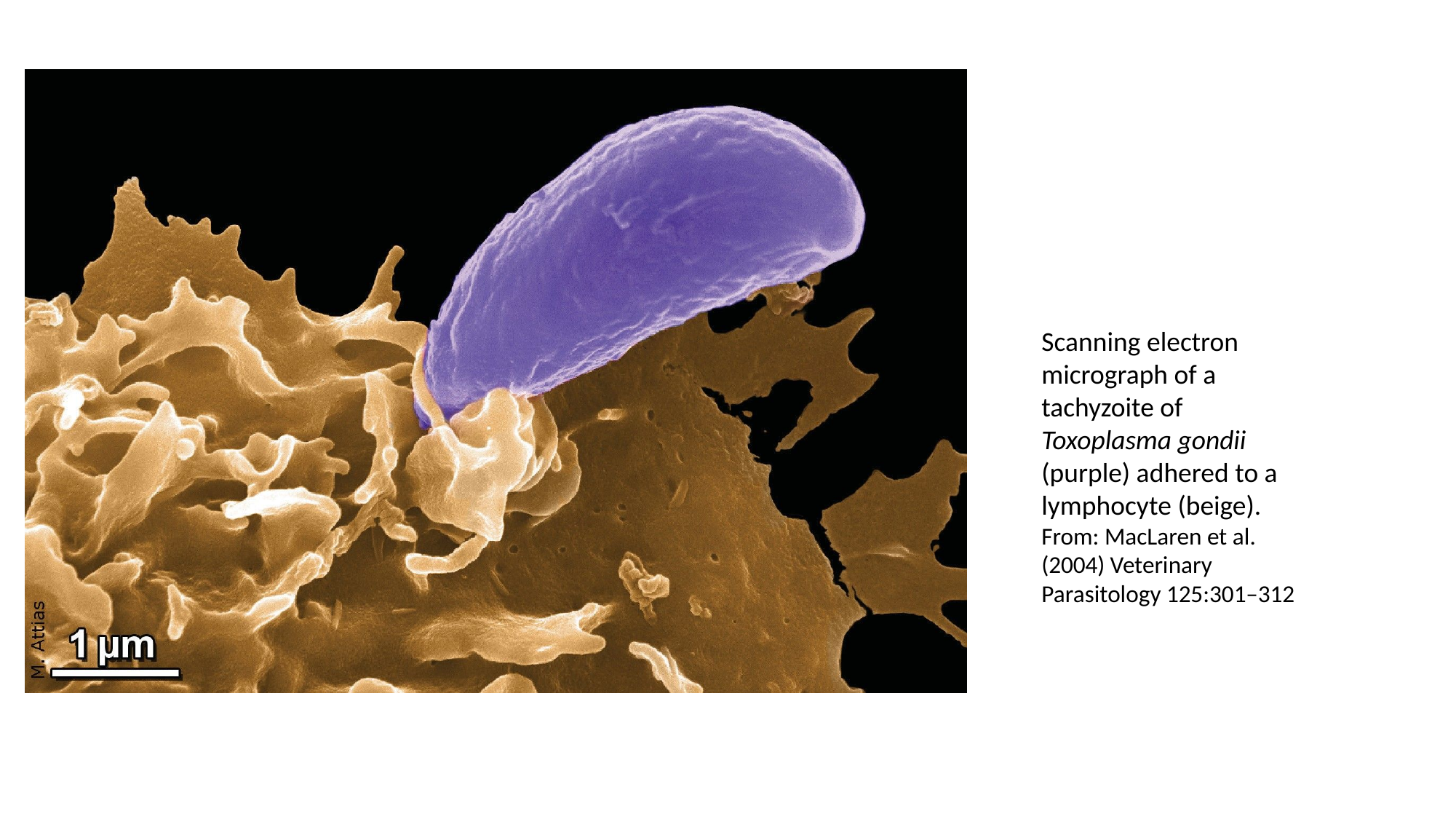

Scanning electron micrograph of a tachyzoite of Toxoplasma gondii (purple) adhered to a lymphocyte (beige).
From: MacLaren et al. (2004) Veterinary Parasitology 125:301–312

## Slide 26
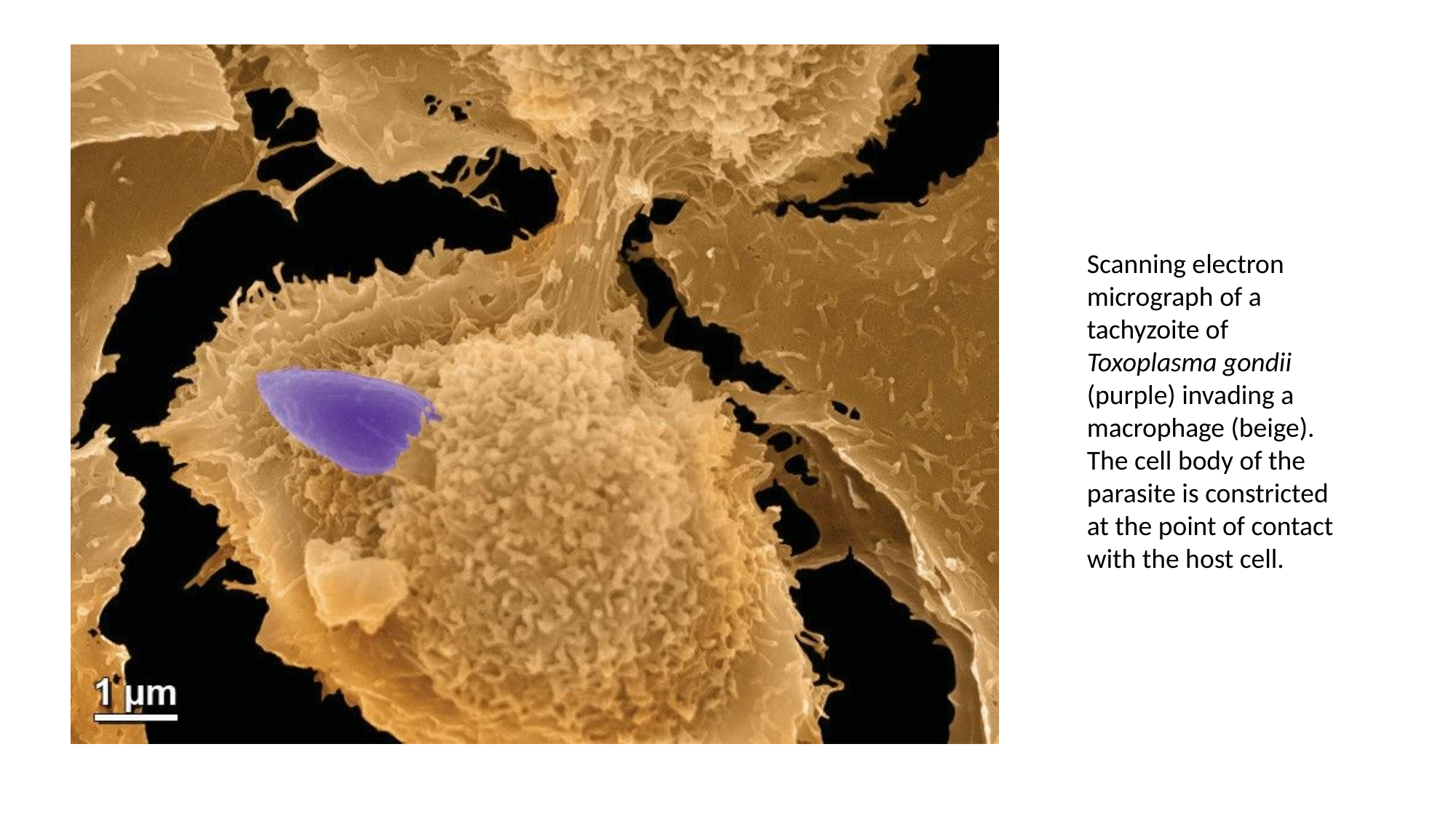

Scanning electron micrograph of a tachyzoite of Toxoplasma gondii (purple) invading a macrophage (beige). The cell body of the parasite is constricted at the point of contact with the host cell.

## Slide 27
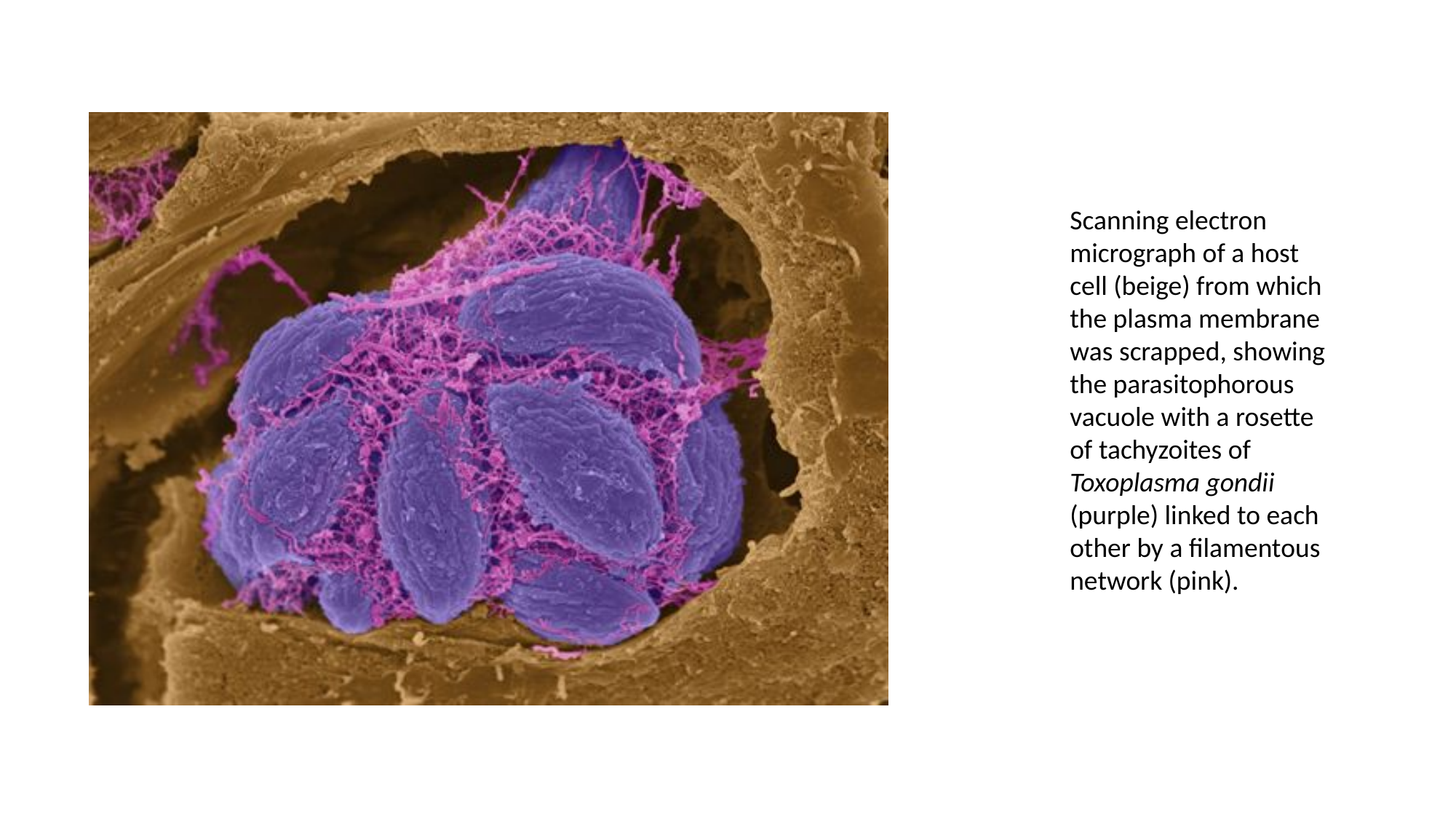

Scanning electron micrograph of a host cell (beige) from which the plasma membrane was scrapped, showing the parasitophorous vacuole with a rosette of tachyzoites of Toxoplasma gondii (purple) linked to each other by a filamentous network (pink).

## Slide 28
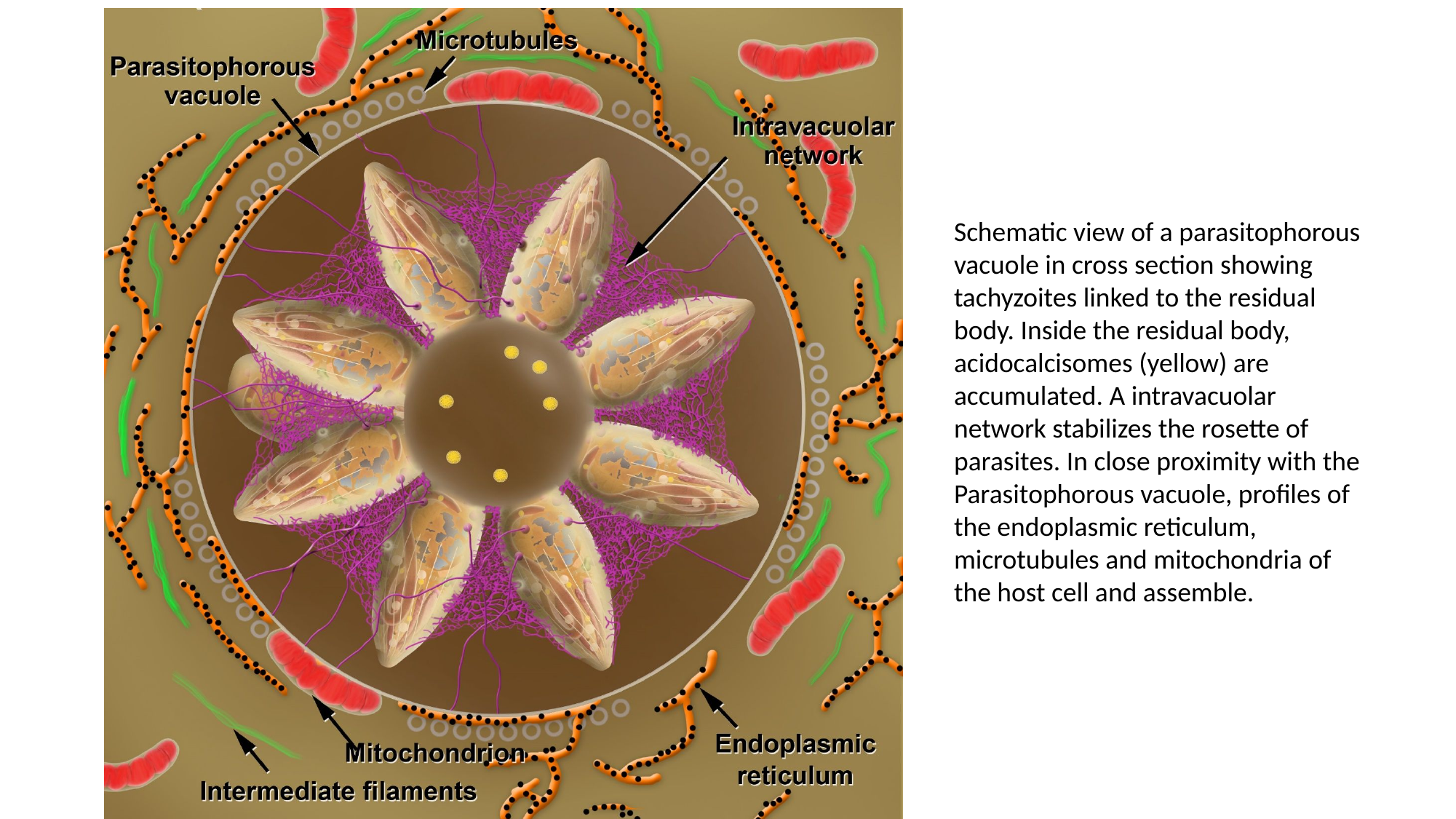

Schematic view of a parasitophorous vacuole in cross section showing tachyzoites linked to the residual body. Inside the residual body, acidocalcisomes (yellow) are accumulated. A intravacuolar network stabilizes the rosette of parasites. In close proximity with the Parasitophorous vacuole, profiles of the endoplasmic reticulum, microtubules and mitochondria of the host cell and assemble.

## Slide 29
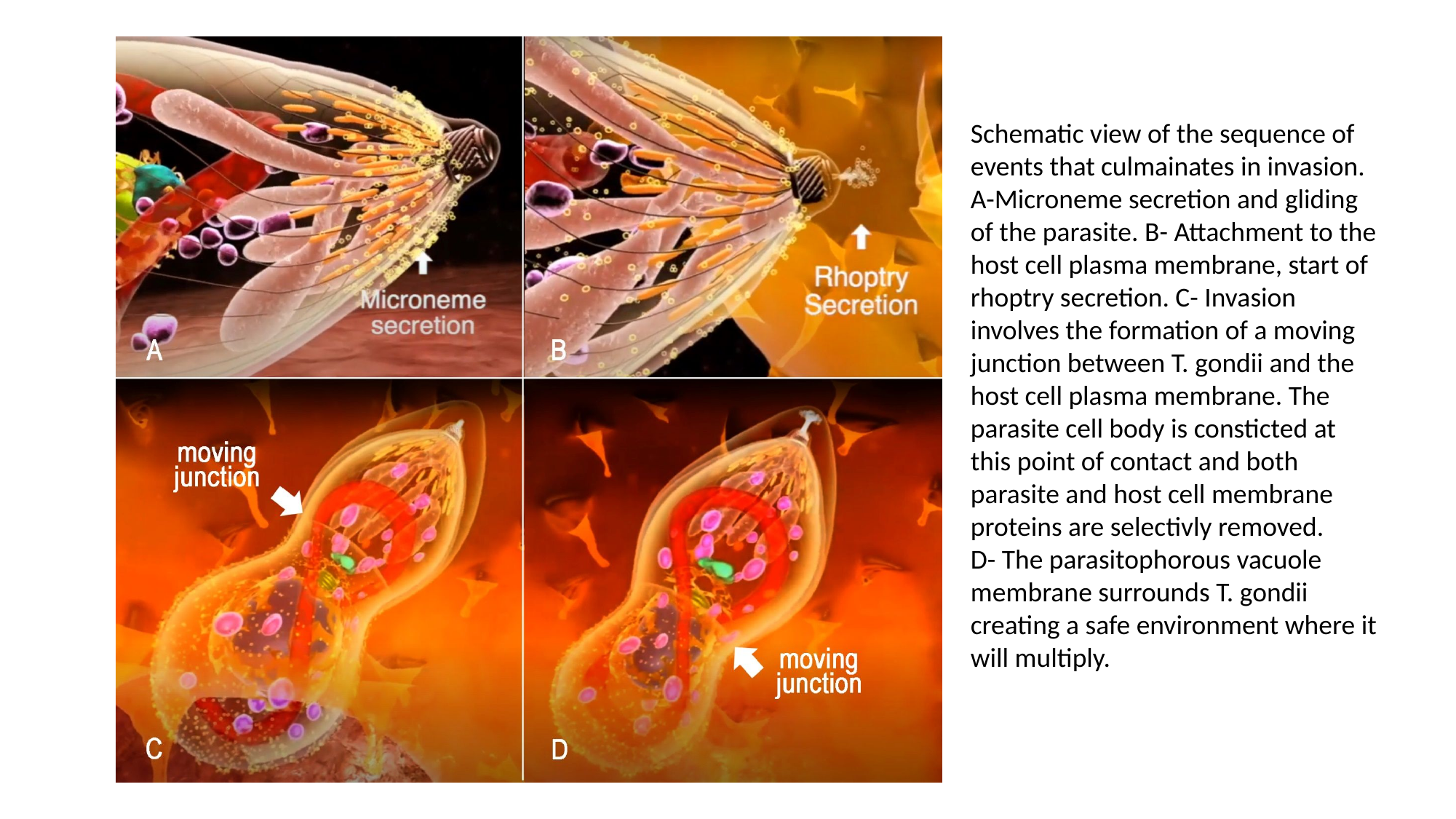

Schematic view of the sequence of events that culmainates in invasion. A-Microneme secretion and gliding of the parasite. B- Attachment to the host cell plasma membrane, start of rhoptry secretion. C- Invasion involves the formation of a moving junction between T. gondii and the host cell plasma membrane. The parasite cell body is consticted at this point of contact and both parasite and host cell membrane proteins are selectivly removed.
D- The parasitophorous vacuole membrane surrounds T. gondii creating a safe environment where it will multiply.

## Slide 30
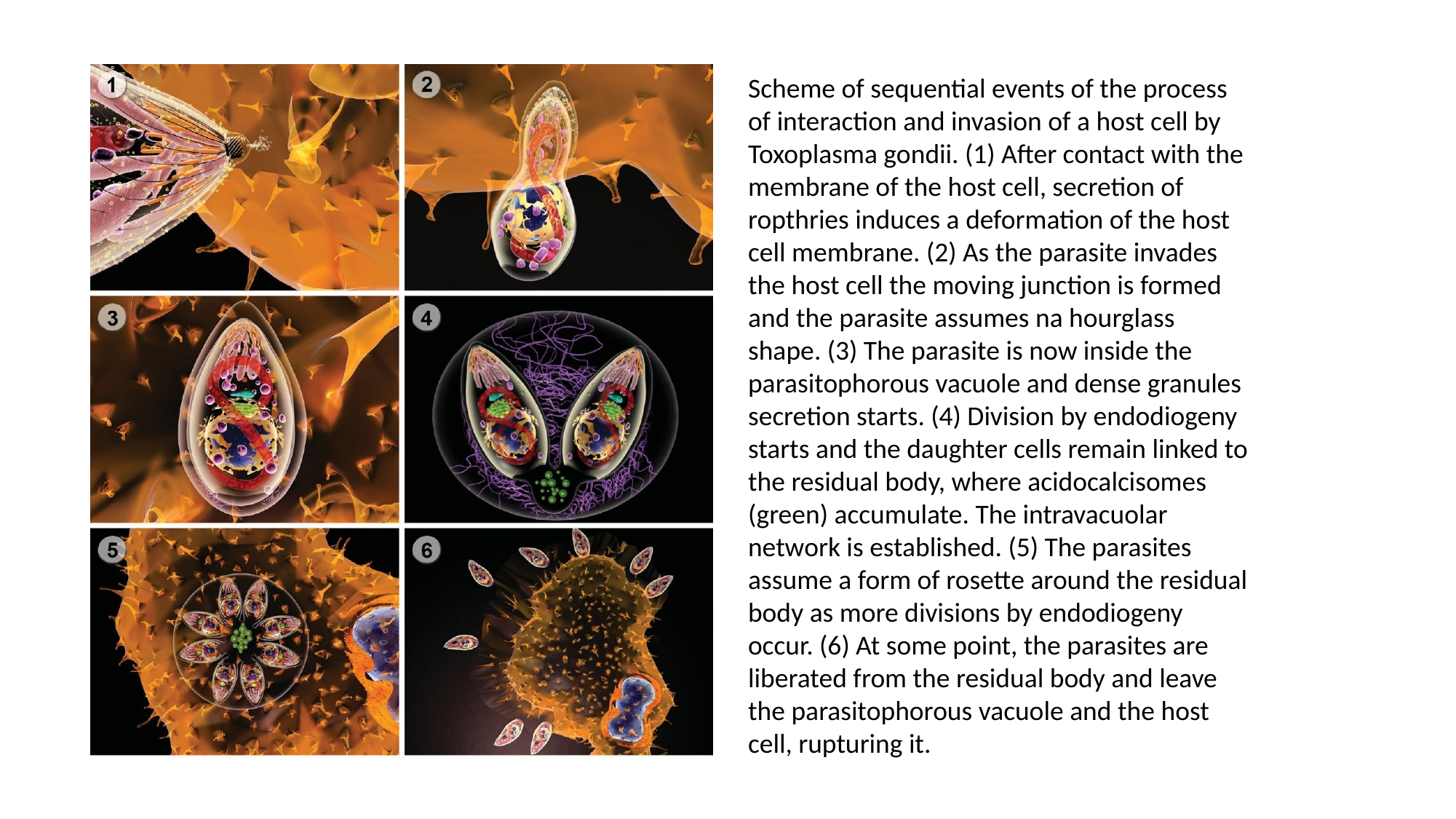

or endodiogeniaApós sucessivos ciclos de divisão
Scheme of sequential events of the process of interaction and invasion of a host cell by Toxoplasma gondii. (1) After contact with the membrane of the host cell, secretion of ropthries induces a deformation of the host cell membrane. (2) As the parasite invades the host cell the moving junction is formed and the parasite assumes na hourglass shape. (3) The parasite is now inside the parasitophorous vacuole and dense granules secretion starts. (4) Division by endodiogeny starts and the daughter cells remain linked to the residual body, where acidocalcisomes (green) accumulate. The intravacuolar network is established. (5) The parasites assume a form of rosette around the residual body as more divisions by endodiogeny  occur. (6) At some point, the parasites are liberated from the residual body and leave the parasitophorous vacuole and the host cell, rupturing it.

## Slide 31
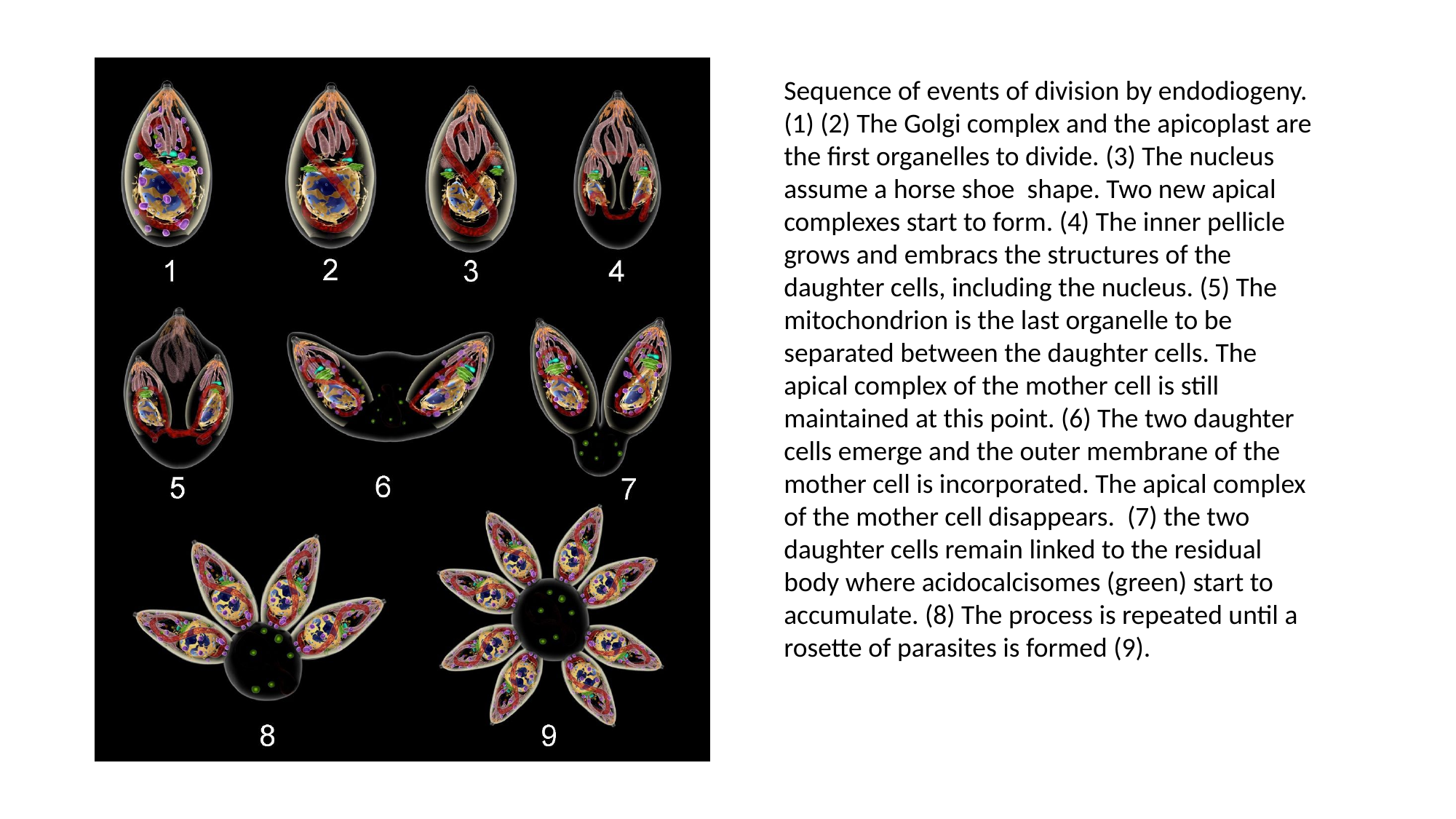

Sequence of events of division by endodiogeny.
(1) (2) The Golgi complex and the apicoplast are the first organelles to divide. (3) The nucleus assume a horse shoe shape. Two new apical complexes start to form. (4) The inner pellicle grows and embracs the structures of the daughter cells, including the nucleus. (5) The mitochondrion is the last organelle to be separated between the daughter cells. The apical complex of the mother cell is still maintained at this point. (6) The two daughter cells emerge and the outer membrane of the mother cell is incorporated. The apical complex of the mother cell disappears. (7) the two daughter cells remain linked to the residual body where acidocalcisomes (green) start to accumulate. (8) The process is repeated until a rosette of parasites is formed (9).

## Slide 32
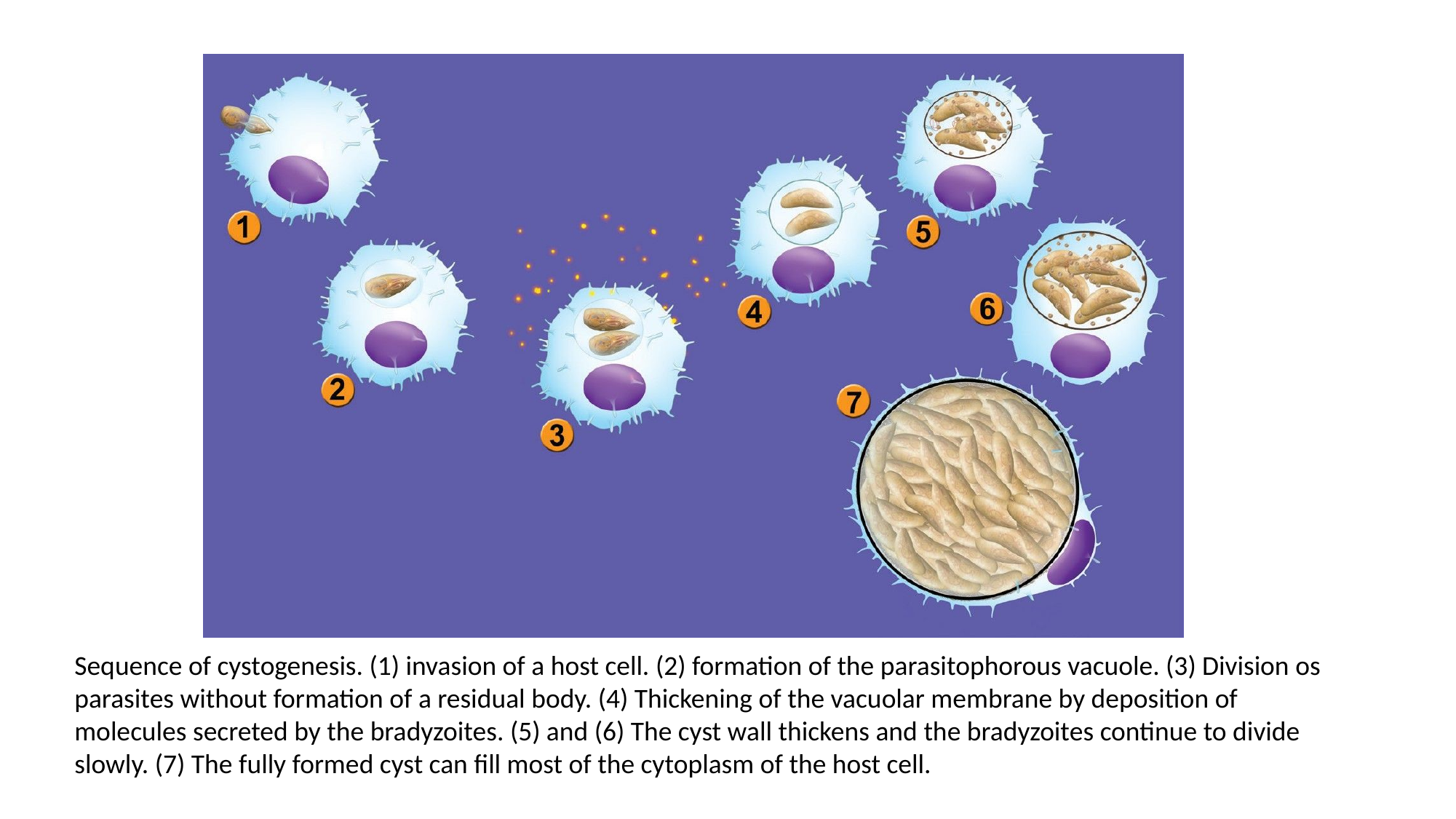

Sequence of cystogenesis. (1) invasion of a host cell. (2) formation of the parasitophorous vacuole. (3) Division os parasites without formation of a residual body. (4) Thickening of the vacuolar membrane by deposition of molecules secreted by the bradyzoites. (5) and (6) The cyst wall thickens and the bradyzoites continue to divide slowly. (7) The fully formed cyst can fill most of the cytoplasm of the host cell.

## Slide 33
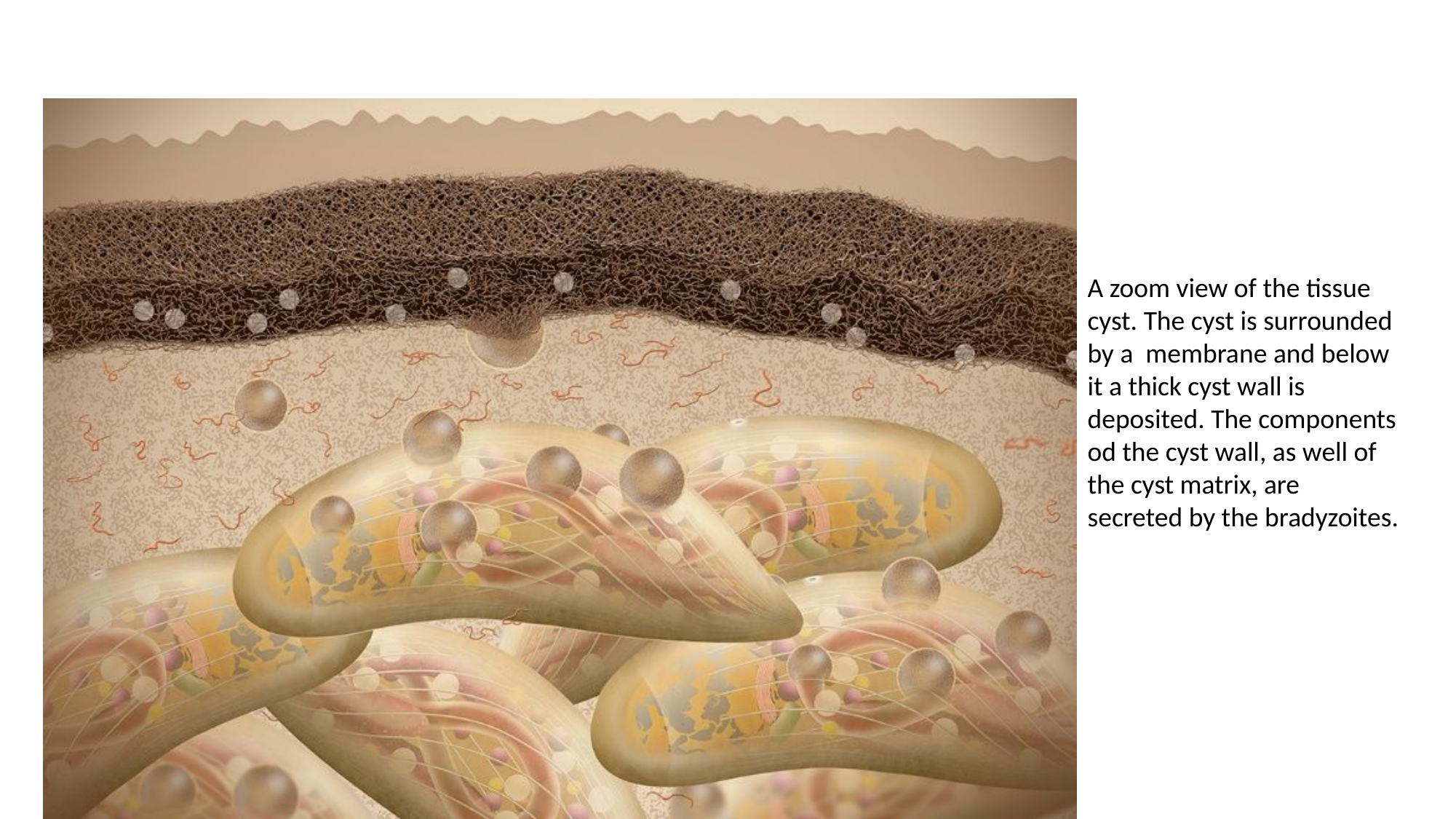

A zoom view of the tissue cyst. The cyst is surrounded by a membrane and below it a thick cyst wall is deposited. The components od the cyst wall, as well of the cyst matrix, are secreted by the bradyzoites.

## Slide 34
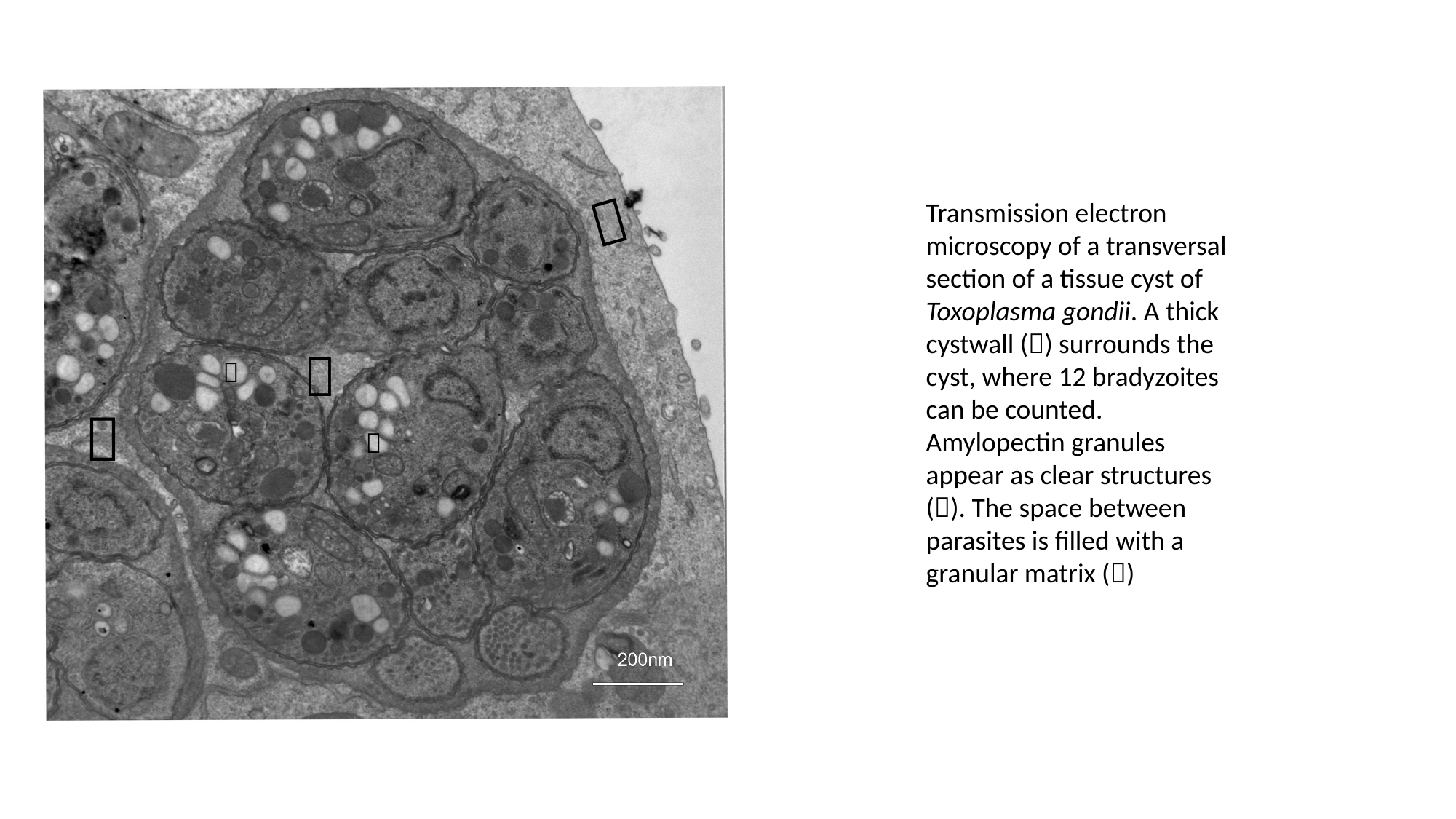


Transmission electron microscopy of a transversal section of a tissue cyst of Toxoplasma gondii. A thick cystwall () surrounds the cyst, where 12 bradyzoites can be counted. Amylopectin granules appear as clear structures (). The space between parasites is filled with a granular matrix ()





## Slide 35
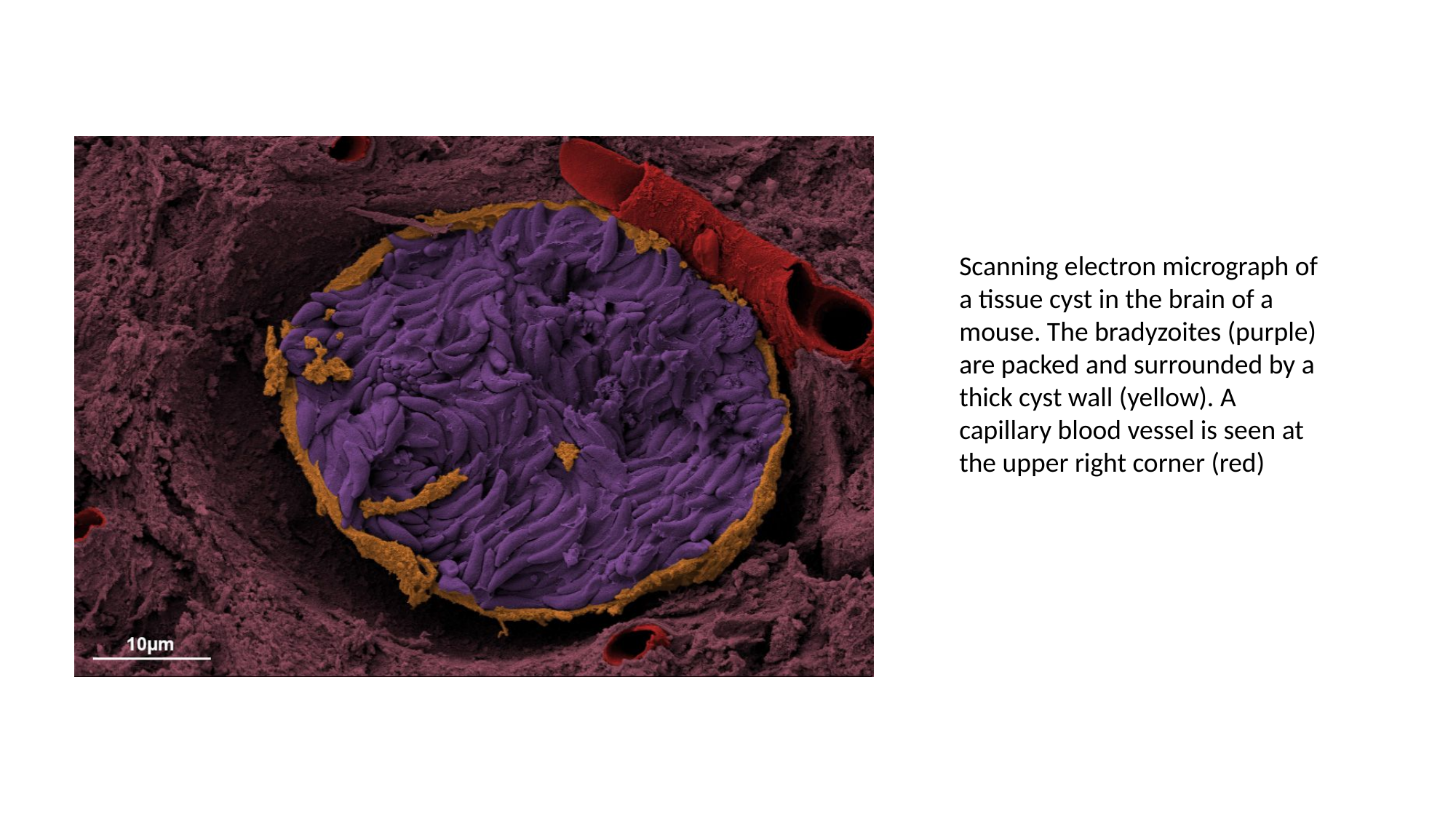

Scanning electron micrograph of a tissue cyst in the brain of a mouse. The bradyzoites (purple) are packed and surrounded by a thick cyst wall (yellow). A capillary blood vessel is seen at the upper right corner (red)
